# Digital public health surveillance: a systematic scoping review

**DOI:** 10.1038/s41746-021-00407-6

**Published:** 2021-03-03

**Authors:** Zahra Shakeri Hossein Abad, Adrienne Kline, Madeena Sultana, Mohammad Noaeen, Elvira Nurmambetova, Filipe Lucini, Majed Al-Jefri, Joon Lee

**Affiliations:** 1 Data Intelligence for Health Lab, Cumming School of Medicine, University of Calgary, Calgary, AB Canada; 2 Department of Community Health Sciences, Cumming School of Medicine, University of Calgary, Calgary, AB Canada; 3 Department of Medicine, Cumming School of Medicine, University of Calgary, Calgary, AB Canada; 4 Department of Electrical and Computer Engineering, Schulich School of Engineering, University of Calgary, Calgary, AB Canada; 5 Department of Critical Care Medicine, Cumming School of Medicine, University of Calgary and Alberta Health Services, Calgary, AB Canada; 6 Department of Cardiac Sciences, Cumming School of Medicine, University of Calgary, Calgary, AB Canada

**Keywords:** Diseases, Public health

## Abstract

The ubiquitous and openly accessible information produced by the public on the Internet has sparked an increasing interest in developing digital public health surveillance (DPHS) systems. We conducted a systematic scoping review in accordance with the PRISMA extension for scoping reviews to consolidate and characterize the existing research on DPHS and identify areas for further research. We used Natural Language Processing and content analysis to define the search strings and searched Global Health, Web of Science, PubMed, and Google Scholar from 2005 to January 2020 for peer-reviewed articles on DPHS, with extensive hand searching. Seven hundred fifty-five articles were included in this review. The studies were from 54 countries and utilized 26 digital platforms to study 208 sub-categories of 49 categories associated with 16 public health surveillance (PHS) themes. Most studies were conducted by researchers from the United States (56%, 426) and dominated by communicable diseases-related topics (25%, 187), followed by behavioural risk factors (17%, 131). While this review discusses the potentials of using Internet-based data as an affordable and instantaneous resource for DPHS, it highlights the paucity of longitudinal studies and the methodological and inherent practical limitations underpinning the successful implementation of a DPHS system. Little work studied Internet users’ demographics when developing DPHS systems, and 39% (291) of studies did not stratify their results by geographic region. A clear methodology by which the results of DPHS can be linked to public health action has yet to be established, as only six (0.8%) studies deployed their system into a PHS context.

## Introduction

Internet technology is now a part of almost everyone’s life. Internet usage among US adults has steadily been increasing from 52% in 2000 to 90% in 2019^1^. Today, 97% of Internet users worldwide are active on social media, and the number of social media accounts per average Internet users has grown from 6.2 in 2015 to around 8 in 2019^2^. The low-cost data stream available on social media and other Internet-based sources is increasingly harnessed by clinicians, patients, and the general public to disseminate insights into disease trends and promote healthy lifestyles and health policies^3,4^. Every minute, people around the world are publicly sharing volumes of personal and communal health information on different digital platforms^5^, such as social media, discussion forums and blogs, and Internet search engines. Digital surveillance data, inspired by the definition of digital epidemiology data by Salathé^6^, is the publicly available user-contributed data not generated with the primary goal of surveillance. This data can provide an inlet to impervious populaces and has become integral to digital public health surveillance (DPHS). Public health surveillance (PHS), as a tool for monitoring and targeting interventions^7^, is the ongoing systematic collection, analysis, and interpretation of data, tightly integrated with the timely dissemination of these data to those who can undertake effective prevention and control activities^8,9^. Apart from the unprecedented volume of digital data, when used appropriately, these online resources can provide an increasingly clear picture of the dynamics and complexities of traditional PHS processes^5,10^. Compared to the data captured through traditional PHS channels, digital resources contain information that can be harnessed to reduce the time to outbreak detection, add more transparency to outbreak information published by the governments, and facilitate public health (PH) responses to emerging diseases and population-related risk factors^10^. These resources can be either used for infodemiology–utilizing digital data for mining, analysis, and information aggregation with the ultimate aim to inform PH and public policy or used for infoveillance– infodemiology methods with the main focus on surveillance^11^. Infodemiology was first formally introduced by Gunther Eysenbach in 2002 to describe the distribution of health information and misinformation on digital platforms^12^ and was later extended to other areas of utilizing digital data for PH research, such as outbreak detection, substance use, and drug utilization^13^.

The interactivity of the Internet and the highly networked, hyperlocal, and contextualized nature of digital data offer an unparalleled opportunity for the public, patients, and health officials alike to communicate and address health issues. Profiling vaccine criticisms^14^, mining patient’s narratives about drug experiences on open-access forums^15^, geospatial tracking of the population during disease outbreaks, providing local and near real-time information to recognition of an outbreak^16,17^, and population-based clustering of behavioural risk factors such as physical inactivity, substance use, and poor diet in large population^18,19^ are examples of realizations of such opportunities.

Effective DPHS requires an understanding of the potentials and pitfalls of digital data for monitoring PH and exploring disease dynamics. Several narrative reviews of the application of digital media in PHS and epidemiology have been published^20–26^. Bernardo et al. reviewed 32 studies published between 2002 and 2011 that utilized search queries and social media data for infectious diseases surveillance^20^. The authors concluded that even though there are challenges associated with the quality of digital data, there have been successful applications of digital disease surveillance since 2006 and their performances in terms of cost, time, and accuracy compare favourably with those of traditional surveillance systems. This was confirmed by a recent scoping review on using web-data for disease surveillance and epidemiology in which Mavragani studied 338 articles from 2009 to 2018 and highlighted the potential of digital surveillance in health informatics research^26^. Newer reviews on this subject have dealt with the popularity of different surveillance domains over time and summarized recent methodological developments mapped to each domain^27,28^. The most recent and extensive digital surveillance review^28^ has pictured a timeline, tracking interest online for PH and solely focused on ethical and validity issues ripe in the digital health monitoring revolution. While the topics covered in our review encapsulate those mentioned, this review will expand on the notion of DPHS by exploring more platforms and a broader context within the PH field. Moreover, a systematic evaluation is absent in the existing reviews, and most encapsulate only certain platforms or diseases/disorders. Therefore, we aimed to provide a comprehensive synthesis of evidence to add to the extant literature filling both of these needs while providing a proportional topic saturation level. Our scoping review also provides details on utilizing digital media in different aspects of PHS. This allows future researchers to identify where the need for future work is ripe and what untapped potentials need more attention in the digital surveillance sphere.

## Results

**Fig. 1:**
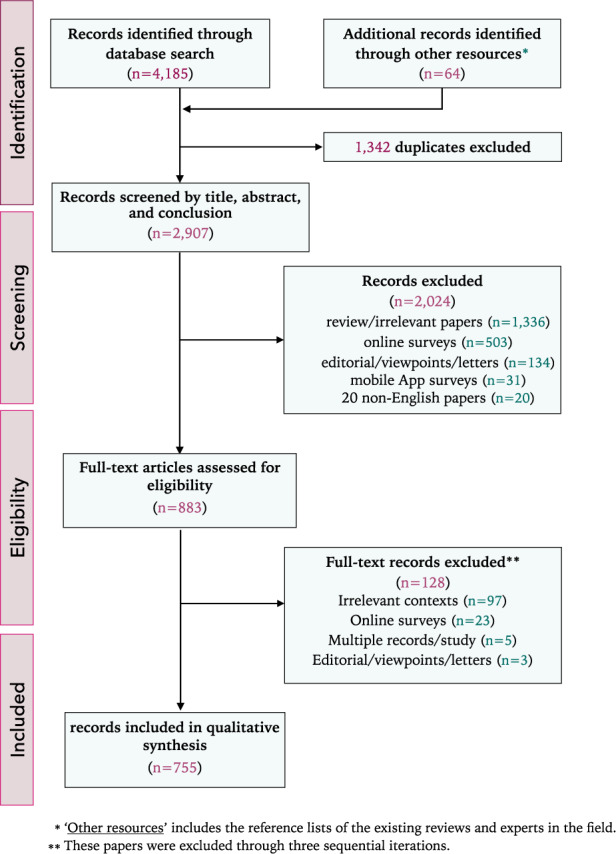
Flow diagram. The overall process of article selection following PRISMA-ScR guideline.

To identify literature on DPHS, we conducted an iterative systematic search with extensive hand searching. Our scoping review was designed, implemented, and reported following the Preferred Reporting Items for Systematic Reviews and Meta Analyses Extension for Scoping Reviews guidelines (PRISMA-ScR)^29^. While there are other well-established guidelines for conducting systematic scoping reviews^30–32^, the detailed reporting guideline, demonstrative examples, and best-practices for large-scale scoping reviews provided by PRISMA-ScR were ideal for our review. The search yielded 4249 articles. Excluding duplicates, we found 2907 studies from which we selected 755 studies of 16 PHS themes, associated with 49 PH categories and 208 sub-categories (Fig. 1). The complete list of included articles is provided in Supplementary Note 5 (a1–a755).

Table 1 lists all PHS themes, their corresponding (sub)categories, and the relevant articles. These themes include behavioural risk factors (BRFs), cancer, chronic disease, communicable diseases, paediatric health, drug utilization, food and nutrition, health practices, health services, environmental hazards, mental health, mortality, vaccine, and urogenital/preconception. Articles that did not coincide with these topics but dealt with PHS were subsumed under the ‘others’ category (e.g., occupational safety). Each paper was contextualized based on the theme it was most closely affiliated with (i.e., BRFs for smoking behaviours and mental health for suicide, depression, bipolar, or eating disorders). More than one context was permitted to capture topics that would fit into two categories (i.e., eating disorders were placed in both the mental health and the chronic disease categories). Many papers harnessed digital data to study the quality of health services; a category was created to reflect this. While those affiliated with health education/campaigns and communication were placed in a communication subgroup and those involving emergency departments, nursing homes, and other health services were grouped in the accessibility and the quality subgroups.

**Table 1:** The hierarchy of public health-related themes studies by the included articles in this review.

Public health themes	Public health category	Public health sub-category
Behavioural risk factors	Smoking^a1^^–^^a53^	E-cigarette/JUUL^a9^^–^^a36^, LCC^a37^^–^^a41^, Hookah^a17^^,^ ^a42^^–^^a45^, Water-pipe^a47^^,^ ^a48^, Heat-not-burn^a49^^,^ ^a50^, E-liquid^a51^^–^^a53^
	Lifestyle^a54^^–^^a89^	Diet^a62–a66^^,^ ^a68^, Physical Activity^a64^^,^ ^a67^^–^^a71^, Weight loss^a72^^,^ ^a73^, Local health^a74^^–^^a82^, Fitness^a83^^,^ ^a84^, Sleep disorders^a85^^–^^a87^, Sexual health^a88^^,^ ^a89^
	Substance use^a90^^–^^a123^^,^ ^a123^^–^^a127^	Alcohol^a91^^–^^a105^, Cannabis/Marijuana^a102^^,^ ^a106^^–^^a123^, Dabbing^a124^^,^ ^a125^, Mephedrone^a126^
	Harassment^a128^^–^^a133^	Sexual^a128^^–^^a130^, (Cyber)bulling^a131^^,^ ^a132^, IPV^a133^
Cancer	Mortality^a134^	Breast^a134^, Lung^a134^
	Prevention^a135^^–^^a141^	Cervical^a135^^,^ ^a136^, Skin^a137^^–^^a140^, Lung^a141^
	Awareness^a142^^–^^a165^	Breast^a145^^–^^a153^^,^ ^a157^, Acute lymphoblastic leukaemia^a154^, Diet^a155^^,^ ^a156^, Smoking^a158^, Prostat^a148^^,^ ^a151^^,^ ^a159^, HNPCC^a160^, Lung^a161^^,^ ^a162^, Cervical^a166^, Skin^a163^, Colorectal^a164^, Genitourinary malignancies^a165^, Ovarian^a150^
	Behavioural measures^a166^^–^^a177^	Throat^a170^, Breast^a175^, Skin^a175^, Melanoma^a175^, Prostat^a175^, Screening^a166^^,^ ^a176^, Pancreatic^a177^
Chronic disease	General^a178^^–^^a193^	Diabetes^a180^^–^^a182^^,^ ^a184^^–^^a189^, Third molar^a190^, Molar incisor hypomineralization (MIH)^a193^
	Musculoskeletal^a194^^–^^a198^	Scoliosis^a194^, Restless leg^a195^, Osteoarthritis^a197^, Gout^a198^
	Eating disorder^a199^^–^^a204^	Obesity^a199^^–^^a201^^,^ ^a203^, Diabetes^a201^^,^ ^a202^
	Cardiovascular^a157^^,^ ^a178^^,^ ^a205^^–^^a211^	Cardiac arrest^a205^, Heart disease^a157^, Oral anticoagulants^a206^, Vasculitis^a207^, Hypertension^a178^^,^ ^a208^, Heartburn^a209^, Venous thrombosis^a210^
	Skin diseases^a212^^–^^a215^	Psoriasis^a213^^,^ ^a214^, Pruritus^a215^
	Lung diseases^a216^^–^^a220^	COPD^a216^^,^ ^a217^, Asthma^a218^^–^^a220^
	Neurological^a142^^,^ ^a221^^–^^a236^	Epilepsy^a222^^–^^a227^, Willis-Ekbom^a228^, Glaucoma^a229^^,^ ^a230^, Multiple sclerosis^a231^^,^ ^a232^, Tinnitues^a233^^,^ ^a234^, ALS^a142^^,^ ^a235^, Fibromyalgia^a236^
	Gastrointestinal^a237^^–^^a239^	Oesophageal^a238^, Crohn’s disease^a239^
	Autoimmune^a240^^–^^a243^	Systemic Lupus Erythematosus (SLE)^a240^^–^^a242^, Rheumatoid arthritis^a243^
Communicable diseases	Outbreaks^a62^^,^ ^a244^^–^^a376^	ILI/Influenza^a62^^,^ ^a245^^–^^a318^, Dengue fever^a301^^,^ ^a319^^–^^a328^, Ebola^a330^^–^^a346^, Zika^a347^^–^^a366^, Avian Influenza^a367^^–^^a369^, Norovirus^a371^^,^ ^a372^, MERS^a373^^,^ ^a374^, Chikungunya^a375^^,^ ^a376^
	Sexually transmitted^a377^^–^^a394^	AIDS^a377^^–^^a379^, HIV^a380^^–^^a389^, HPV^a390^, Syphilis^a392^^–^^a394^
	Infectious diseases^a271^^,^ ^a395^^–^^a433^	Clostridium difficile^a401^, Meningitis^a403^, Measles^a404^^–^^a407^, TBE^a408^, Polio^a410^^,^ ^a411^, Guillain-Barré^a413^, Tuberculosis^a415^, HFMD^a416^, RSV^a417^, Scarlet fever^a418^, Plague^a419^^–^^a421^, Cholera^a434^, West-nile virus^a422^, Pertussis^a271^^,^ ^a423^^–^^a426^, Candida auris^a427^, Lyme^a428^^–^^a430^, Mayaro virus^a431^, Malaria^a432^, Hepatitis^a433^
Paediatric health	Awareness^a322^^,^ ^a435^^–^^a437^	DSFCs^a322^, Paediatric Fever^a435^, SIDS^a436^, Obesity^a437^
	Birth defects^a438^^–^^a440^	Pharmacoepidemiologic^a438^, Intrauterine growth restriction (IUGR)^a440^
	General^a441^^,^ ^a442^	Accident^a441^, Chicken pox^a442^
Drug utilization	Awareness^a443^^–^^a452^	Anabolic-androgenic steroid (AAS)^a448^, Alternative medicine^a449^, Stem-cell therapy^a450^, Codeine^a451^, Antiretroviral^a452^
	Drug safety/side effects^a453^^–^^a464^	Statins^a456^, Illicit Pharmacies^a457^^–^^a459^, Bisphosphonate^a460^, Psyclone^a461^, Zolpidem^a462^, Antimicrobial stewardship^a463^
	Adverse reaction^a277^^,^ ^a465^^–^^a484^	Atorvastatin^a473^, Psychiatric drugs^a475^, Glucocorticoid-related^a480^, HIV^a481^
	Drug abuse^a485^^–^^a503^	Opioid^a485^^–^^a495^, Fentanyl^a496^, Heparinoid^a497^, Recreational^a498^^,^ ^a499^, Adderall^a500^, Antidepressants^a501^, Sea salt^a502^
	Post-marketing^a504^^–^^a506^	Sitagliptin^a504^, Antidepressant^a505^, Opioid^a506^, Loperamide^a503^
Food and nutrition	Food safety^a507^^–^^a517^	(Un)healthy^a509^^–^^a511^^,^ ^a517^^,^ ^a518^, Legislation^a512^, Food poisoning^a513^^,^ ^a514^, Food-borne illnesses^a515^^,^ ^a516^
	General^a519^^–^^a523^	Marketing^a520^^,^ ^a521^^,^ ^a523^, Online recipes^a522^
Health practices	Outcomes^a524^^–^^a527^	Rejuvenation^a524^, Breast reconstruction^a525^, Tanning^a526^^,^ ^a527^
	General^a231^^,^ ^a528^^–^^a537^	Dietary supplements^a530^^,^ ^a531^, Sunburn^a533^, Physical therapy^a534^, Organ donation^a535^, Bariatric surgery^a536^, Plastic surgery^a537^
Health services	Quality assessment^a333^^,^ ^a538^^–^^a549^	Nursing care^a333^^,^ ^a539^^,^ ^a540^, Hospitals^a541^^–^^a543^, Emergency departments^a544^^,^ ^a545^, Dermatologic care^a546^, Surgery^a547^^,^ ^a548^, Radiology^a549^
	Accessibility^a550^^–^^a553^	Emergency departments^a550^^,^ ^a551^, Physical therapy^a553^
	Health communication^a93^^,^ ^a554^^–^^a584^	Awareness^a93^^,^ ^a555^^–^^a567^^,^ ^a571^, Patient support^a568^^–^^a578^, Health reforms^a579^^–^^a581^, Crisis^a582^, Heat alert^a583^, outbreak alert^a584^
Environmental	Pollen counts^a585^^–^^a596^	Seasonal Allergic Rhinitis^a585^^–^^a590^, Epistaxis^a591^, Air pollution^a592^^–^^a595^, Sinusitis^a596^
	Syndromic^a597^^–^^a601^	Heat wave^a597^^–^^a601^
	Water quality^a602^^–^^a605^	Fluoridation^a602^^–^^a604^, Lead^a605^
	Disaster/Crisis^a606^^–^^a608^ Winter	Storm^a606^, Tornado^a607^, Earthquake^a608^
Mental health	General^a62^^,^ ^a609^^–^^a644^	Suicide^a612^^–^^a626^, Post-Traumatic Stress^a628^, Depression^a62^^,^ ^a629^^–^^a637^, Stress^a638^^,^ ^a639^, Bipolar^a640^^,^ ^a641^, Loneliness^a642^^,^ ^a643^, OCD^a644^
	Emotion analysis^a645^^–^^a650^	Disaster/crisis^a646^^–^^a648^, Outbreaks^a649^^,^ ^a650^, Suicide^a651^
	Stigma^a644^^,^ ^a652^^–^^a656^	Suicide^a652^^,^ ^a655^, Anxiety^a653^, Self-harm^a656^
	Neurodevelopmental^a637^^,^ ^a657^^–^^a668^	ADHD^a657^, ASD^a658^, Schizophrenia^a637^^,^ ^a659^^–^^a663^, Dementia^a664^^–^^a667^, Psychotic^a668^
	Eating disorder^a669^^,^ ^a670^	Anorexia nervosa^a669^
Mortality	General^a61^^,^ ^a671^^–^^a675^	Awareness^a671^, Socio-demographics^a672^, Perinatal^a673^, Stroke^a674^, Accident^a675^
	Behavioural factors^a489^^,^ ^a676^^,^ ^a677^	Substance use^a489^^,^ ^a676^, Suicide^a676^, Social activity^a677^
Vaccine	Decision making^a678^^–^^a708^	Paediatric^a688^^–^^a691^, HPV^a692^^–^^a704^, Influenza^a705^, Herper Zoster^a706^, Polio^a707^, Measles^a702^
	Adverse event^a709^^,^ ^a710^	Influenza^a709^, Anxiety-related^a710^
	Coverage^a329^^,^ ^a711^^,^ ^a712^	Influenza^a329^, HPV^a711^^,^ ^a712^
	Awareness^a713^^–^^a722^	HPV^a713^^–^^a718^, Flu^a719^, Rotavirus^a720^, Measles^a721^, Autism^a722^
Urogenital/Preconception	Genital^a62^^,^ ^a723^^–^^a729^	Abortion^a723^, C-section^a725^, Pregnancy^a62^^,^ ^a726^^–^^a728^, Morcellation^a729^
	Renal^a730^^–^^a732^	Kidney stone^a730^^,^ ^a731^, Dialysis^a732^
	Urinary^a733^	Urinary Tract Infection (UTI)^a733^
Others	Toothache^a734^^–^^a737^	Teathing^a737^
	Sexual dysfunction^a738^^,^ ^a739^	Peyronie^a738^, Ejaculatory dysfunction^a739^
	Animal health^a740^^,^ ^a741^	Slaughterhouse^a740^, Marine litter^a741^
	Disease burden^a742^^,^ ^a743^	Skin diseases^a743^
	Occupational safety^a744^^–^^a747^	Chemical Poisoning^a744^, Accidents^a745^, Silicosis^a746^, Injuries^a747^

An article could be linked to only the ‘category’ column if it did not address any sub-categories listed in the sub-category column.

*TBE* tick-borne encephalitis, *DSFC* delayed subaponeurotic fluid collections, *ADHD* attention deficit hyperactivity disorder, *HNPCC* hereditary non-polyposis colorectal cancer, *HFMD* hand, foot and mouth disease, *RSV* respiratory syncytial virus, *OCD* obsessive compulsive disorder, *IPV* intimate partner violence, *ALS* amyotrophic lateral sclerosis, *SIDS* sudden infant death syndrome.

**Fig. 2:**
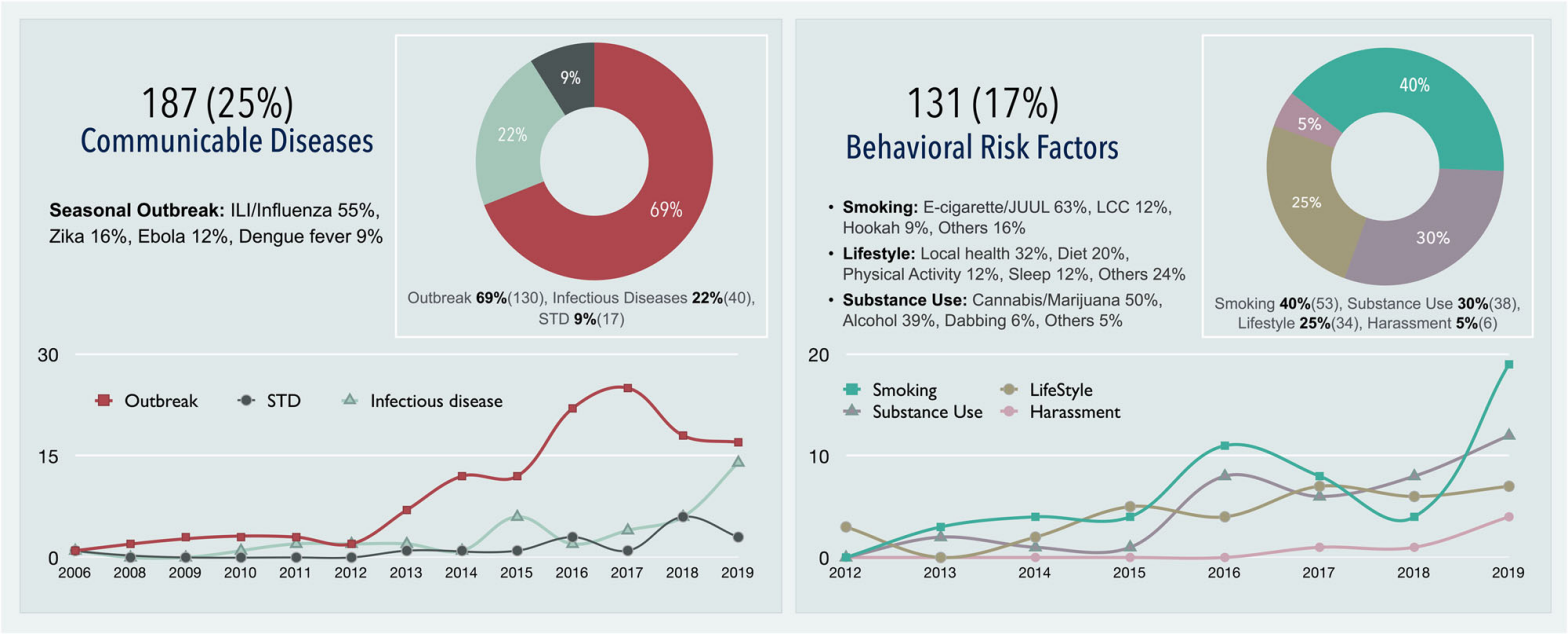
The most frequently addressed PHS themes. The temporal trends of the two most prevalent themes of DPHS systems in the literature.

The surveillance theme with the most number of publications was the ‘communicable disease’ surveillance at 25% (187). The stark rise in the volume of communicable disease publications coincides with the 2016 Zika outbreaks. In 2016, ILI-focused studies were the most common ‘communicable disease’ studies (53%), following a similar distribution to the overall trend of all such studies. In 2017, Zika-focused studies were the most common (36%). Publications in 2017 saw a greater variety of health events studied (Fig. 2).

A large proportion of BRFs studies can be linked to policy changes. The peak of e-cigarette publications in 2016 and 2017 (Fig. 2) may be attributed to growing international concerns in the preceding years as policymakers noticed vaping products marketed towards youth and young adults. A congressional report in the USA^33^ and the WHO FCTC^34^, both in 2014, may have prompted increased research in this area in subsequent years. Similarly, the sudden academic interest in cannabis research in 2016 may result from the rapid legalization and decriminalization of medicinal and recreational cannabis in the preceding years (Fig. 2).

### Countries, affiliations, and surveillance systems

**Fig. 3:**
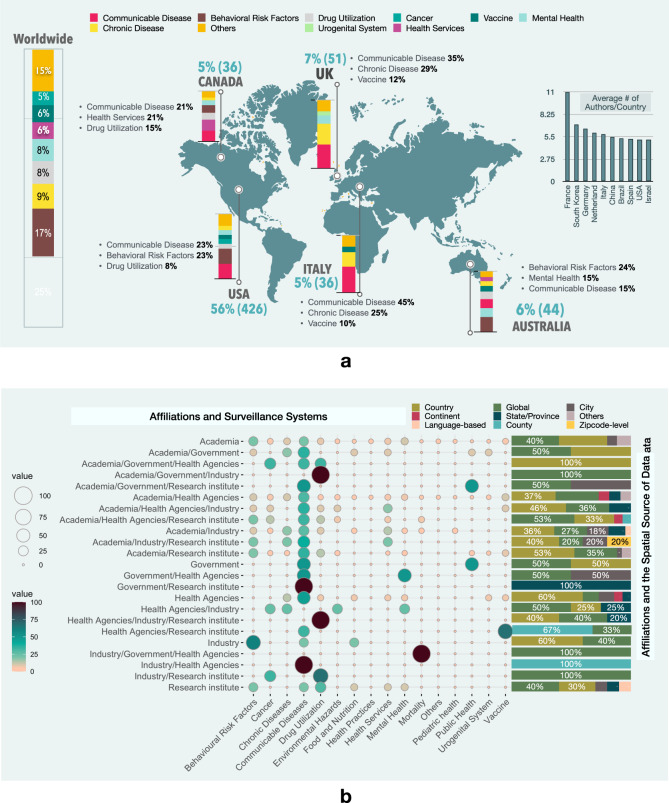
The distribution of studies based on country and affiliation, mapped to different PHS themes. **a** Top five countries and PHS themes. **b** The frequency of different combinations of affiliations, PHS themes and the average number of authors per country.

A total of 79% (593) of the studies included in this review were published by researchers from the USA (426), UK (51), Australia (44), Canada (36), and Italy (36). The most common surveillance theme researched among these countries include communicable diseases, BRFs, chronic disease, drug utilization, and mental health (Fig. 3a).

More than 94% (707) of the studies involved authors affiliated with academia, from which 460 studies are only academia affiliated. Only 3% (23) of studies have an author affiliated with governments, with ten of them studied communicable diseases, and three studied the general aspects of PH (Fig. 3b). None of these studies investigated the vaccine, environmental hazards, or health practices surveillance systems. The studies utilized datasets with no geographic focus (36%, 268) are dominated by BRFs, communicable, and chronic diseases. The majority of studies with geographically focused datasets used country-level data, and only 0.7% used ZIP-code level datasets. The studies in this category are dominated by communicable diseases, BRFs, and health services surveillance systems (Fig. 3b).

### Social media platforms and surveillance systems

**Fig. 4:**
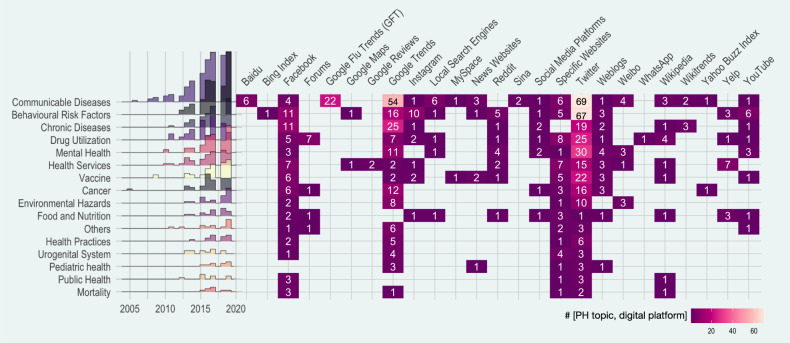
The temporal trend of surveillance domains associated with a cross-tabulation of surveillance domains and social media platforms (darker shades represent smaller values). Surveillance systems that utilized more than one platform were assigned to multiple, and the maximum allowed being five. Studies that investigated more than five platforms are mapped to the ‘Social Media Platform’ column.

Starting from 2005, the three most common digital platforms studied were, in descending order, Twitter, Google Trends, and Facebook. Their numbers increasing sharply from less than three studies per year in 2009 until reaching 78, 49, and 13 studies, respectively, in 2019. Google (Flu) Trends (GT and GFT) are utilized by 41% (76) of publications on communicable diseases, among which 57% (43) of studies aimed to predict outbreaks and seasonal diseases. From 69 studies that utilized Twitter to study communicable diseases, 32% (22) mined tweets for outbreak prediction. Facebook, Instagram, and YouTube were mainly utilized to study BRFs, focusing on smoking, substance use, and lifestyle. Fifty percent of studies that used Yelp investigated topics related to ‘health services’, while this number for Facebook, YouTube, Instagram, and GT is less than 2% (Fig. 4). Almost half of the studies on ‘mental health’ used Twitter data, and 11 studies used GT to observe the seasonal patterns of internet search volume in a wide range of mental health terms. More details about the digital platforms used by the included studies are presented in Supplementary Note 3.

### Methods—data collection duration

**Fig. 5:**
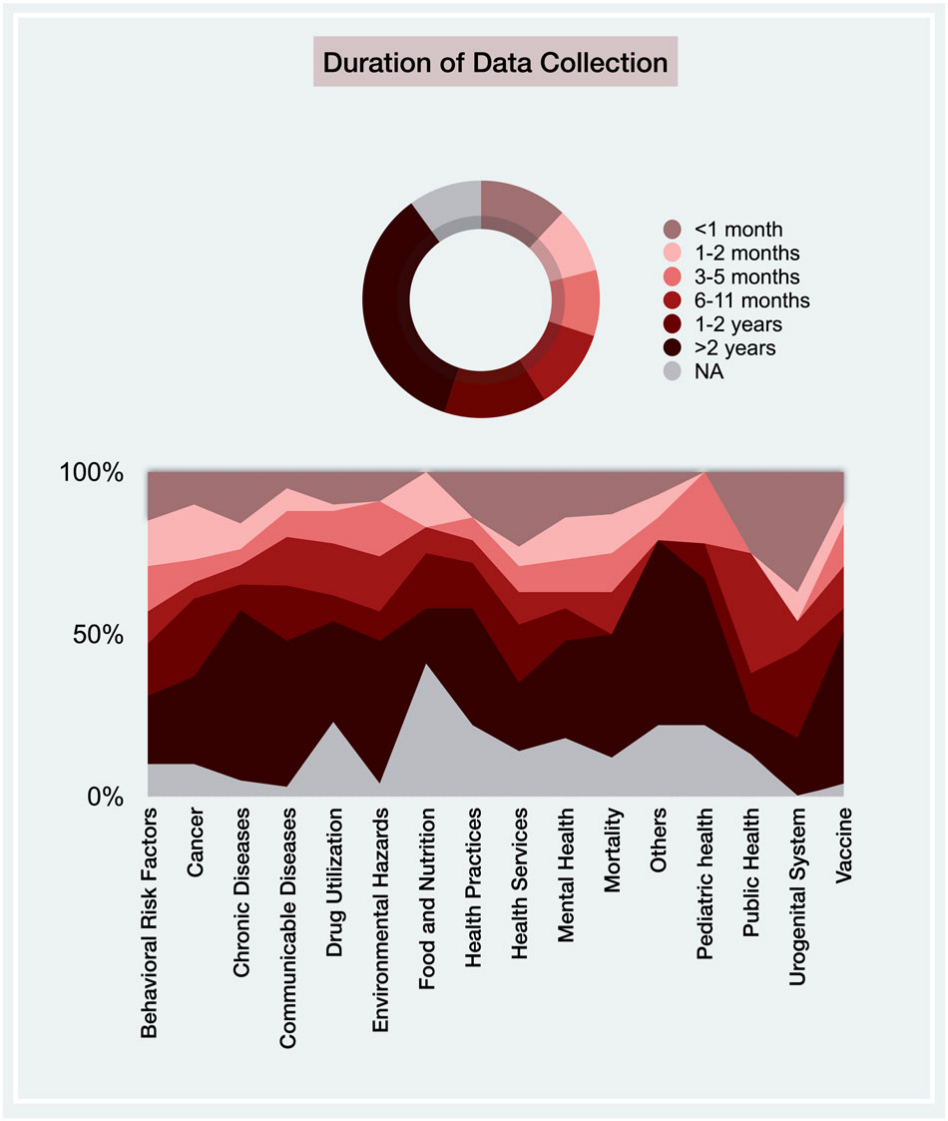
Data collection duration. The differences in data collection duration across included studies and the proportion of articles within each time frame across all surveillance systems.

There was a wide variability in data collection duration (Fig. 5). Overall, 36% (268) of the included studies had a duration of more than 2 years, 14% of such studies had a duration of 1–2 years, and 40% of studies had a duration of less than 1 year, with a greater proportion covering less than 6 months. All surveillance themes followed similar distributions, with some notable exceptions: 53% of chronic disease publications had a duration greater than 2 years, while this number for communicable diseases and BRFs themes is 44% and 21%, respectively. Notably, urogenital publications had the shortest duration of data collection, with 34% lasting less than 1 month. Indeed, from Table 1, the associated PH categories (i.e., genital, renal, and urinary) are events with a typically short onset and duration. Moreover, 98% (740) of studies implemented their analysis based on secondary data—the longitudinal data that are sometimes collected months or years after the event occurred^35^. Thus, surveillance systems that are developed based on secondary data analysis are more useful for long-term rather than short-term interventions^35^.

### Methods—objectives, data analysis, and findings

**Fig. 6:**
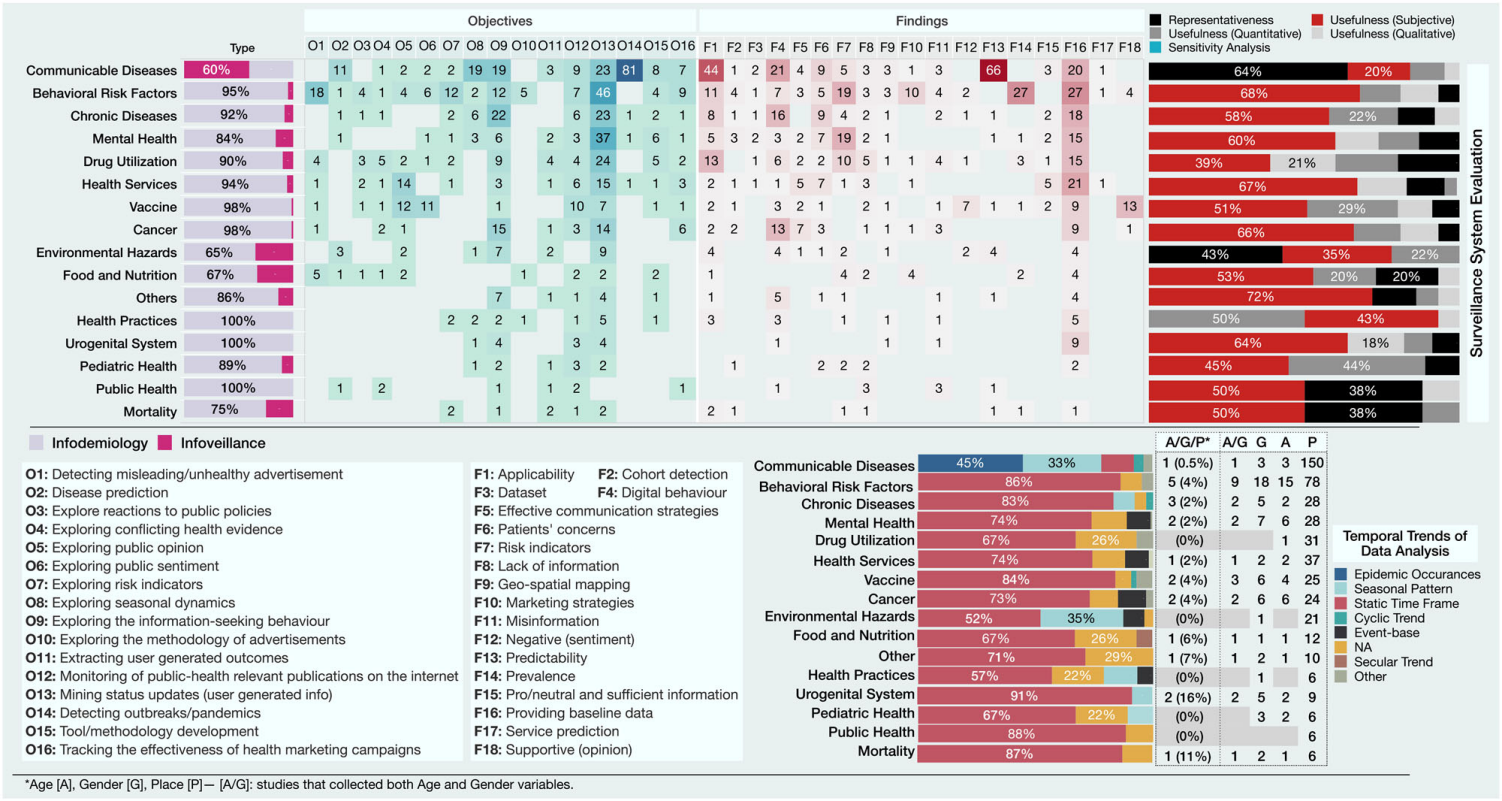
The top charts illustrate the mapping between PHS topics and objectives [O], and findings [F] of their corresponding studies, the frequency of infoveillance/infodemiology studies for each topic, and the techniques used by the included publications to evaluate the effectiveness of their proposed approach in addressing the key objectives of a surveillance system. The bottom charts represent the temporal trends of data analysis used by the included studies and the frequency of articles that identified each of the age/gender/place in their datasets.

We classified the studies based on their overall data collection and analysis methodology (Fig. 6). Studies with the main focus on mining, analysis, and information aggregation to inform PH and public policy were placed in the infodemiology category (77%). Studies that emphasized surveillance were classified as infoveillance (23%)^11^. Not surprisingly, 112 (60%) of publications on communicable diseases are infoveillance studies. This could be because of the great potentials of the existing digital data such as search queries and access logs to explore the public’s digital behaviour and detect epidemic outbreaks. The main objectives of infodemiology publications were to mine user’s status updates (O13, 32%), and the most common finding was providing baseline data (F16, 23%). Conversely, the infoveillance studies were dominated by the ones that showed the predictability (F13, 28%) and applicability (F1, 22%) of digital data for outbreak detection (O14, 31%).

#### Objectives and findings

From the manual content analysis of the objectives and findings of the included studies, eighteen distinct strands of investigations emerged. ‘Providing baseline information’ on risk patterns and trends in the occurrence of various health events (22%, 163), exploring the ‘applicability’ of utilizing web-based platforms in PHS systems (13%, 98), and ‘identifying user’s digital behaviour’ for evaluating the correlation between online activity and incidence and temporal trends of risk factors (11%, 84) are the top three (Fig. 6).

Detecting unhealthy advertisements (O1) is the second most frequent objective associated with BRFs publications, with 89% (16) of them related to smoking (69%: e-cigarette/JUUL and LCC). Seventy five percent (12) of these publications showed the prevalence of advertising smoking behaviour (F14), and 19% (3) explored the marketing strategies used by smoking vendors (F10). This implies the utilization of digital resources as marketing platforms for different smoking brands, which may carry major PH risks (Fig. 6). Exploring public opinion (O5) and sentiment (O6) towards immunization are the most common objectives in the publications on vaccine surveillance (48%, 23). These objectives are mainly mapped to supportive attitudes (F18) and negative sentiments (F12), respectively. These findings imply the need to design and implement appropriate educational information tailored to different social media platforms, with the main focus on the users who are at risk of excessive exposure to anti-vaccine information. For example, men are far more likely to express a negative opinion about HPV immunization than women^a695^, or users who are more often exposed to negative opinions about HPV vaccines are more likely to post negative messages subsequently^a697^.

Twenty one percent (13) of publications on drug post-marketing/utilization reported on the applicability (F1) of using Internet-based data in exploring drug safety/adverse drug reaction (ADR) (85%), post-marketing (8%), and drug abuse (7%). Interestingly, two studies showed that Twitter might not be a useful platform for this system, as the ADR reports on Twitter usually underrepresent specific drugs and often do not meet the FDA criteria required for reporting an ADR^a468^^,^ ^a476^. This is in line with a recent systematic review that shows the prevalence of ADR reports on social media varies from 0.2% to 8% of all postings^36^. Sixty three percent (19) of mental health studies reported risk indicators (F7), from which 73% (14) were related to self-harm or suicide attempts. Applying linguistic analysis methods^a652^, exploring time-varying features related to suicide risk factors^a625^, mapping digital behaviour of different age groups to these indicators^a610^^,^ ^a622^, and emotion analysis^a645^ are sample exploratory techniques discussed by the publications in this category. In oncology, exploring the digital behaviour of users (F4) can be used to identify temporal trends of cancer risk factor queries, cancer incidence and mortality, and interests in cancer screening, compared to other information-seeking domains^37^. Thirty eight percent (5) of studies placed in the [Cancer/F4] category used GT^a167^^,^ ^a169^^,^ ^a170^^,^ ^a175^ and Yahoo Buzz Index (YBI)^a168^ to conduct search-based cancer surveillance and 23% (3) mined user-generated content (O13) on Twitter^a161^^,^ ^a171^^,^ ^a173^ to study cancer information-seeking behaviours and the incidence of some types of cancer.

#### Age/gender/place and temporal trends of data analysis

Given the primary purpose of surveillance is the monitoring and assessment of the overall health status of population subgroups^9^, analyzing time, demographics (age, gender), and place is a critical component of any PHS system^35^. Since the rise of Internet-based data usage in PHS, great strides have been made in identifying place, gender, and age from anonymous self-reported information on the Internet. Mining users’ profile information^a37^^,^ ^a199^, content analysis^a132^^,^ ^a162^^,^ ^a727^, population survey^a318^^,^ ^a508^, mapping to local demographic data^a630^, and utilizing third-party tools^a120^^,^ ^a201^ are some sample techniques used by the studies included in this review to explore these variables. However, relatively few studies have systematically incorporated these epidemiologic parameters in their data analysis, despite the value of these indicators in identifying risk groups (Fig. 6). Moreover, it is worth noting that questions of validity, mis-classification of users^38^, and under-counting caused by sampling bias^39^ are challenges that still need to be addressed. The data analysis of 61% (460) of studies reflects the results of a specific time window, which, excluding communicable diseases, is the most common type of temporal analysis in all reported surveillance systems. Conversely, temporal analysis of the ‘epidemic occurrence’ of a disease and ‘seasonal patterns’ have been the commonly used inferential analytic approaches in analyzing communicable diseases data (Fig. 6). Thirty-two percent (242) of studies did not capture any of the age/gender/place variables for their data analysis, with the majority of them coming from the BRFs category.

#### Evaluation of the surveillance system

Seventy-four percent (561) of studies evaluated the usefulness of their proposed DPHS system by drawing a mapping between the system’s objectives and outcomes. Among these, 361 (48% of total) studies were evaluated subjectively, 116 (15%) used quantitative methods such as statistical analysis and machine learning (ML) techniques, and 85 (11%) used surveys/qualitative analysis methods. Twenty-five percent (192) of studies used the ‘representativeness’ approach to explore the extent to which the characteristics of reported events can accurately represent the incidence of actual health events^40^ (Fig. 6). About two-thirds (64%, 120) of the articles on communicable diseases used this approach, followed by studies on environmental hazards (43%, 10). Given that the rate calculation (e.g., seasonal/cyclic incidence of a health event) required for measuring the inclusivity of a system needs an entirely separate data system maintained by an external agency (e.g., Centers for Disease Control and Prevention (CDC) ILI data), utilizing this approach might be more challenging for the other surveillance systems.

#### Data types and analysis methods

**Fig. 7:**
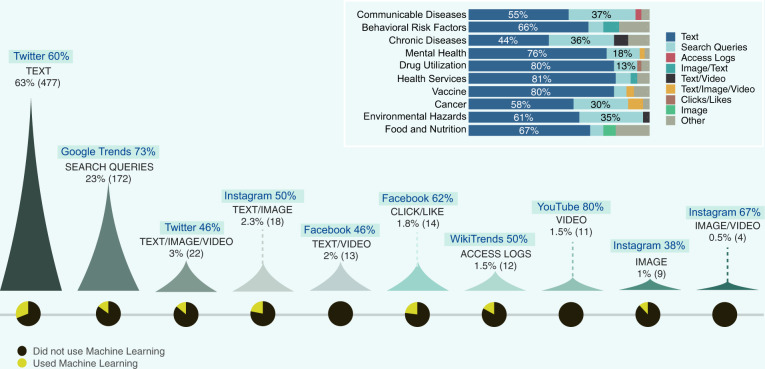
Data types and analysis methods. The mapping between data types used by the included studies and the PHS systems, platforms, and the use of machine learning.

Figure 7 summarizes the frequency of different data types used by the included studies, their mapping to different PHS themes, and the proportion of the studies that applied ML techniques to process each data type. Textual data are the category with the highest number of ML applications (31%), and none of the studies that utilized video data used ML. This meagre rate, of course, reflects the fact that there are several pitfalls to the process of analyzing Internet-based data. ‘Search queries’ is the second most frequent data type. Given its popularity, considerations must be given to the limitations of search query analysis, such as the dynamic changes of health information-seeking behaviour, the uncertainty of information seeker representativeness (e.g., some searches may be generated by bots or news reports), and the limited geographic data that can be gleaned from this data type.

## Discussion

### Key findings

We report a comprehensive scoping review to summarize and synthesize evidence from a large and heterogeneous body of literature studying DPHS. The growing body of evidence of DPHS reflects the chronological availability of new digital platforms and new data mining and ML techniques. Our findings show the huge effect of mass media on the public’s information-seeking behaviour. Exploring these behaviours can help PH officials tailor their messages to address PH interests and improve healthcare delivery.

Digital data can help portray the dynamics of PHS systems and allow PH professionals to pinpoint the general concerns or needs of the public during infectious disease events to create location-specific campaigns. For example, the finding that there is no association between dental caries and toothache-related information-seeking behaviours among South American Google users can reinforce the unfamiliarity of this population about the relationship between dental pain and the final stages of chronic oral diseases^a735^.

Our findings show a higher prevalence of digital surveillance systems for communicable diseases (25%, 187). One possible reason for this is that topics such as seasonal outbreaks and epidemics, sexually transmitted and infectious diseases, can be coalesced in this category, making it a far-reaching one. Another reason may be the ease of using relative search volumes for various outbreak-related and infectious diseases using Google Trends, access logs on other social media platforms, as well as the fear/hype surrounding infectious diseases and different epidemics such as H1N1, Ebola, and Zika. Very few papers dealt with ‘disease burden’ (0.3%) and ‘occupational safety’ (0.5%), which came as a surprise given the excellent availability of Google Trends data.

The surveillance themes studied by each country appear to follow international trends (Fig. 3a). Interestingly, the USA and Australia had a greater proportion of articles studying BRFs, which can be attributed to international differences. For instance, according to the UN World Drug Report (2016), the prevalence of cannabis users in the USA and Australia in 2015 surpassed that of the European average by roughly 4%^41^. Although cannabis remains the most commonly used illicit drug in both countries, Australia has seen a drastic rise in the use of amphetamines and other illicit drugs since 2012. The USA holds the largest market for e-cigarettes. Also, it has the most reported vaping-related illness, particularly in young people. Furthermore, both countries have significantly more overweight or obese people. Recent reports show that 67% of Australian adults and 71% of American adults (over the age of 20) are overweight. Indeed, these factors, combined, may contribute to increased research in smoking, lifestyle habits and illicit substance use, which in turn increases the proportion of behavioural risk factor publications.

While the use of user-generated information on the Internet certainly shows promises, especially from the standpoint of providing an alternative and inexpensive solution to PHS, questions remain regarding the validity and generalizability of social media and Internet data^28^. Given the limited length of data (e.g., a tweet), different language styles between Internet users, and no restriction on their writing style, user-generated content often contains a high amount of noise, making the automatic information extraction and classification of free-text data challenging and time-consuming. Moreover, many concerns have been raised about the correctness and the quality of health-related digital data and the detrimental effects that misinformation can have on PH^42^. This concern with misinformation was also apparent during the 2014 Ebola outbreak^a335^ or the Zika outbreak in 2016^a354^^,^ ^a357^^,^ ^a359^^,^ ^a366^. Table 2 lists the included studies that investigated the spread of inaccurate or incomplete health-related information on the Internet. The number of studies in this category increased from 21 in 2015 to 60 in 2019, with a spike in 2017, comprising 8% of all included studies. Digital misinformation can quickly spread but difficult to refute. As listed in Table 2, the majority of research on PH-related misinformation has focused on communicable diseases, and BRFs surveillance systems and most of the reported misinformation by the included studies have proliferated via Twitter, news websites, and Facebook, respectively. Sixty-seven percent (40) of these studies analyzed textual data, and 18% (11) contained video data. Among the studies without geographic focus, the investigation is dominated by those of drug utilization, chronic diseases, and vaccines, respectively. Interestingly, studies that investigated misinformation in a specific geographical zone mainly focused on BRFs, communicable diseases, and health services surveillance systems. Despite this long-standing effort, there is still a clear need for a valid assessment of the potential for harm associated with digital health misinformation and its relative impact for different surveillance systems.

**Table 2:** Studies that detected inaccurate or incomplete information in the context of DPHS, mapped to various PHS themes/categories and digital media platforms. [FB]: Facebook, [NW]: News Websites, [SW]: Specific Websites, [YA]: Yahoo Answers, [WA]: WhatsApp, and [YT]: YouTube.

Surveillance System (n)	Subgroup	FB	Forums	GT	NW	Reddit	SW	Twitter	WA	Weblogs	Weibo	Wikipedia	YA	YT
Public health(10)	General	a748					a749	a750, a751				a752		
	Disease comparison							a753						
	Dental	a754			a754	a754		a754						
Behavioural risk factors(17)	Smoking and genetic	a46						a46						a46
	E-cigarette	a27					a9, a27	a27						a27
	Alcohol	a103					a93	a91, a103		a93				
	Cannabis							a107, a119						a117
Cancer(4)	Breast							a153						
	Diet						a155							
	Awareness	a165						a165						
Drug utilization(8)	General								a755					
	ADR		a478					a468						
	Psyclone						a461							
	Awareness											a444, a445		
	Alternative medicine											a449		
	Stem-cell therapy						a450							
Paediatric health(3)	DSFCs									a322				
	IUGR						a440			a440				
Chronic diseases(5)	Obesity							a203						
	COPD			a216										
	Heart disease							a157						
	Hypertension	a208												
	Scoliosis	a194												
Communicable diseases(12)	Zika	a354, a359					a357	a366	a359					
	Avian influenza										a369			
	Food-borne illnesses						a516							
	Clostridium difficile													a401
	HPV						a390						a390	
	Ebola										a335			
	Lyme						a430							
Reproductive health(2)	C-section						a725							
	Pregnancy						a727							
Health communication(5)	Knee arthroscopy											a563		
	Suicide						a562							
	Tinnitus	a233						a233						a233
Mental health(2)	ADHD													a657
	Psychotic							a668						
Vaccine(4)	HPV						a713, a714	a697						
	Decision making							a683						
Environmental(4)	Water fluoridation						a602	a602				a602		a602
Food and nutrition(1)	General											a519		
Health practices(2)	Rejuvenation		a524				a524							
Mortality(3)	Awareness	a671						a671						a671
Occupational safety(1)	Brain injury							a747						

### Limitations of the included studies

First, we found that 61% (460) of studies conducted cross-sectional analysis (Fig. 6), and thus they were unable to evaluate the longitudinal or temporal dynamics of their findings. These findings might change over time, and longitudinal analysis would be needed before being utilized by PH decision-makers. Ten percent (75) of studies did not even report the time scale of their analysis and only reported the analysis results. Even if the temporal analysis is unrevealing, the usefulness of a PHS system needs to be assessed periodically to ensure that it is serving a useful PH function^35^.

**Fig. 8:**
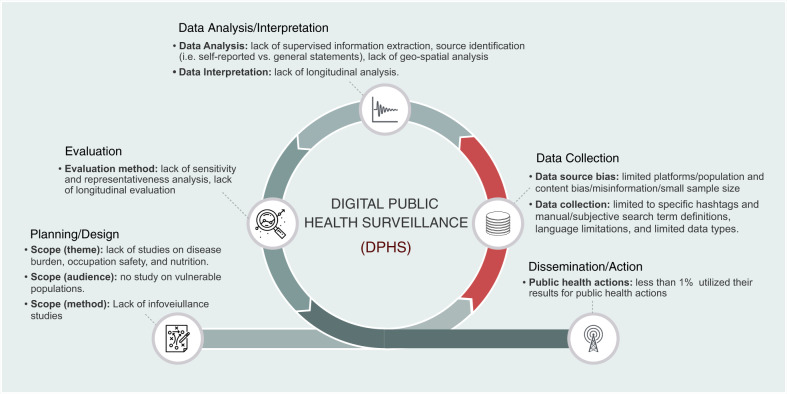
The overall iterative process of a public health surveillance system. The coloured phase in red highlights the key difference between traditional and digital public health surveillance. The summary of current limitations of research on DPHS discussed throughout this review, is mapped to and listed below each activity of the process.

Second, the majority of the studies that utilized digital data for PHS (77%, 581) had an exploratory nature and attempted to gather information and data to inform PH officials about the potential of DPHS in different areas of PHS (Table 1). Among these studies, 28% (165) provided baseline data (F16 in Fig. 6), 17% (98) investigated the applicability and feasibility of digital data for PHS (F1), and 28% (163) studied users’ digital behaviour and their concerns and opinions about different aspects of PH (F4, F6, F12, and F18). While these studies provide some valuable information on the potential of DPHS, they represent only the first three steps of a PHS process (i.e., planning&design, data collection, and data analysis, Fig. 8) and are limited in real-world evaluation (i.e., sensitivity and representativeness analysis) and system deployment.

Third, around 40% (299) of studies were limited by sample size and scope, as they used labour-intensive methods such as manual coding and qualitative analysis. The majority of the 219 studies that applied NLP methods used rule-based and lexical matching techniques such as topic modelling, sentiment analysis, and language modelling. These methods can only extract abstract themes at a high level, and the subjectivity in the interpretation of their results might limit the generalizability and the accuracy of the findings of these studies.

Fourth, the content bias is another limitation of the included studies in our review. User-generated content on the Internet is highly biased as it reflects information that people are comfortable having revealed and may not represent the real spectrum of their feelings/experiences. In addition to this, our study’s results show that among the 554 studies that used text, image, or video data types, only 20% (111) took into account whether their findings were associated with the user’s personal experience (i.e., self-reported) or not. Thus, there is a clear need for studies capable of determining and mitigating content biases that affect the formation and adoption of digital data for PHS.

Fifth, the final link in the surveillance chain is the timely dissemination of the system’s findings to the general public or PH officials for action. Of the articles included in this review, only six (0.8%) linked their results for public health action. While there is a clear need for rigorous methodologies by which the results of DPHS systems can be converted into usable information, vigilance is still needed regarding the efficacy and safety of these findings to forgo the unintended consequences of these results on PH decisions.

Sixth, while the anonymity of Internet users enables individuals with discreditable stigma to reap the benefits of supportive communication on digital media^43,44^, the difficulty of ascertaining demographics poses several unresolved questions regarding the inherent population biases of Internet users with different cultural background or socioeconomic status. Demographics for most digital platforms are not nationally representative and skewed toward younger age groups and users with higher levels of education^45,46^. We found that no studies assessed digital media utilization for vulnerable populations (e.g., low-income, older adults, or people with a disability) who are underpresented on different digital platforms. Studies on detecting social bots are scarce. Considering the radically increasing rate of childhood obesity with the subsequent adolescent onset of nutrition-related chronic conditions such as diabetes and cardiovascular diseases^47,48^, which could be due to the massive exposure of adults and children to unhealthy food and beverages through product placements and promotional advertisements on different digital platforms^49–51^, this topic is vastly underreported by the research on DPHS.

Seventh, among the 379 studies that utilized Twitter, Facebook, and Instagram, 41% (156) confined their analysis to content that was attributed with specific hashtag(s). These studies represent a biased population of users, and they may have skewed the data by excluding contents relevant to the health event under study. Furthermore, from the full-text of the 581 studies that did not use hashtags, we manually extracted the methodologies they employed to query the Internet or filter their collected data and found that the majority (71%, 411) used only their subjective opinion and 10% (57) used the existing literature to define their search keywords. Trend analysis (i.e., Google correlates) and ontology-based keyword extraction were used by 6% (37) and 5%(29) of the studies, respectively. Only 1% (7) of studies used automatic algorithms such as ML, NLP, or lexical analysis to extract context-sensitive keywords. Considering the rapid changes in web search behaviours, the uncertainty regarding the representativeness of pre-defined keywords, and the highly context-sensitive nature of health-related events, keyword querying alone might not be suitable in DPHS^a634^.

Eighth, furthering the population bias of the social media data, 82% (619) of studies analyzed only one platform, potentially leading to false positives. For example, Twitter content on poliomyelitis differs significantly from other English-language media content^a410^. Eighty five percent (638) of studies are limited to English-language content. Given that some of the addressed health-related issues by the included studies may be prevalent in countries other than the USA and countries with large English-speaking populations, the language bias can limit the conclusions to English-speaking populations. For example, the largest burden of cervical cancer is in non-English-speaking countries such as countries in Africa, Asia, and South America^a135^, while only English-tweets were reviewed to study this topic.

Ninth, although the health outcomes of different PHS systems are highly location-dependent and might vary based on local healthcare policies^52^, the results of 36% (274) of the studies reported in this review were not segmented by geographic location, thus limiting the conclusiveness of their results. For example, while search engine data may be a useful tool to study the temporal dynamics of the pollen seasons in Ukraine and China^a587^^,^ ^a595^, the agreement between search queries and pollen concentrations in France is usually poor^a588^. Similarly, in studies that investigated drug abuse in the context of varying policies, digital data were shown to be a valuable indicator of drug-related communications^a114^^–^^a116^^,^ ^a123^. However, this limitation is inherent in some of the digital platforms such as Yelp, Reddit, and WikiTrends as they do not make the location of the poster or visitor readily available. More details about the challenges of using specific digital platforms for different PHS topics are presented in Supplementary Note 4.

### DPHS and its challenges

Despite the improvements enabled by digital technologies, the overall process of PHS research has remained constant and contains five main systematic and iterative activities^9,53^. Figure 8 illustrates the overall process of DPHS and summarizes the limitations of existing research on DPHS discussed earlier by mapping them to different activities of this process. During the course of this review, we found that the main differences between traditional and DPHS lie in how and for what purposes the data are generated and utilized (highlighted in Fig. 8). Following the definition of digital surveillance data used to define the scope of this review, a DPHS system uses digital data voluntarily generated by the public, regardless of the main objectives of the task at hand. Digital data generated through online surveys or polls with a pre-defined surveillance goal or digital content that is not publicly available cannot be considered digital surveillance data. This methodological difference between traditional and digital PHS systems helps explain the challenges mapped to different DPHS activities (listed in Fig. 8). Data source bias (e.g., limited platforms and content/population bias), data collection limitations (e.g., subjective filtering), challenging data analysis due to the complexities of unstructured digital data, and lack of sensitivity analysis for evaluating DPHS systems due to the limitations of mapping digital data to national and real-world data are some of the key challenges that still need to be addressed in future work.

### Limitations of the scoping review

This study has some limitations. First, the terminology in the context of DPHS is not yet established in a consistent way, and our search strings may not have captured all the existing evidence. To mitigate this, in addition to a literature review and involving domain experts, we used language modelling and lexical analysis to find the context-sensitive terms that present the field. Second, papers excluded based on our criteria may yet prove relevant to DPHS, despite decisions made by three reviewers. Finally, although we have tried to discuss some of the most important findings in the literature through intuitive and detailed visualization techniques, it is impossible in a limited space to detail all the aspects of the studies utilized digital media for PHS. The supplementary dashboard we present alongside this study presents more interactive results. However, we believe that a more broadly based review of each of the surveillance systems presented in this paper provides necessary contexts for DPHS.

## Methods

### Search strategy and selection criteria

For this scoping review, we searched Global Health, Web of Science, and PubMed for articles published in English, up to January 2020. For each search string, we also searched the first ten pages of Google Scholar that displayed 20 results per page to ensure we had included all highly cited articles relevant to the scope of our review. To define the search strings for automated search, we used literature review, manual content analysis, and Natural Language Processing (NLP), including language modelling (i.e., the probability of a given sequence of words in a document) and lexical association analysis (i.e., the co-occurrence of words), to explore the context-sensitive terms relating to DPHS (Supplementary Note 1.1 and Supplementary Table 1). The reference lists of the included articles were also screened for additional relevant studies not identified during the automatic search. To assess the performance of the developed search strategy, the sensitivity of more than 200 search strings were tested using a quasi gold standard^54^ set of 80 articles. These articles were selected manually from studies published in four public health journals from 2017 to 2018 (Supplementary Note 1.2 and Supplementary Table 2).

We included all studies published in English and investigated digital data to implement a surveillance system directly (infoveillance) or mined, analyzed, and aggregated information from digital resources to inform PH and public policy for PHS purposes (infodemiology). Digital data in this paper, regardless of its type, refer to the publicly available user-contributed content on the Internet that was not generated with the main purpose of supporting PHS^25^. Digital data sources can be categorized into social networking sites (e.g., Facebook, Twitter); Internet search data (e.g., Google (Flu) Trends); collaborative websites (e.g., Wikipedia); content sharing websites (e.g., YouTube, news websites); and blogs and forums (e.g., Reddit, Yelp)^55^. Thus, we excluded all PHS studies that actively collected data by conducting online surveys, digital polls, and interviews. Moreover, articles that used digital data for personal surveillance (i.e., monitoring potentially exposed individuals to detect early symptoms^35^) were excluded from this review. We also excluded studies that utilized digital data for purposes other than PHS. For example, studies that reported on leveraging the social structures of digital platforms for health education and research recruitment, or studies that only contributed to developing new ML techniques for PHS were not eligible for inclusion. Full details of the inclusion/exclusion criteria are listed in Supplementary Note 1.4.

The titles and abstracts of the articles identified by the search strategy were manually screened by three reviewers independently for eligibility according to the inclusion and exclusion criteria. Disagreements about eligibility were settled by discussion among the three reviewers. One reviewer manually assessed the full text of included publication and identified additional papers that did not meet the eligibility requirements.

### Data analysis

A data extraction form was developed and independently piloted on 50 publications by three reviewers. Seven reviewers extracted data from the included articles and two reviewers manually reviewed all fields of the data extraction form and resolved discrepancies by reviewing the full text of the included studies. The following data were extracted from the included papers: authors’ affiliation, number of authors, year of publication, country of authors, country of data collection, platform(s) under study, surveillance theme and (sub) category, objective and findings, the temporal trend of data analysis, surveillance type, age/gender/place mapped to the data, the language of data, analysis methods (i.e., quantitative, qualitative, machine learning), data type (e.g., text, image, video, and search query), duration/start of data collection, evaluation methods, and the methodology of using digital resources for PHS.

To summarize the extracted data from the included articles, we used a descriptive-analytical method to extract contextual and process-oriented information from each study^56^. A qualitative analysis was also conducted using NVivo 10^57^, a software programme for qualitative analysis, to chart the descriptive results and findings of the included studies. We tabulated a hierarchy of digital surveillance systems reported by the included studies and used narrative visualizations to report the findings of this review. We also developed an interactive visual dashboard (available at https://rpubs.com/zshakeri/dphs_dashboard) to provide insights into the findings with a multidimensional and more granular conceptual structure that is difficult to articulate in text alone. More details about the dashboard are provided in Supplementary Note 2.

As the primary purpose of this study was to perform scientific paper profiling on internet-based user-generated data in the PHS context, we did not critically appraise the methodological quality of the included studies. However, we will comment on the methodological limitations that could have affected their results and implications.

## Supplementary information

Supplementary Information

## Data Availability

All data generated or analyzed during this review are included in this article and its supplementary information files.
