# Supplementary material for: Digital public health surveillance: a systematic scoping review
Source: NPJ Digit Med. 2021 Mar 3;4:41. doi: 10.1038/s41746-021-00407-6 (PMC7930261; doi:10.1038/s41746-021-00407-6)
Supplement: Supplementary file 1 — Supplementary Information [file 41746_2021_407_MOESM1_ESM.pdf]

Supplementary Information

Digital public health surveillance: a systematic scoping review

Zahra Shakeri Hossein Abad, Adrienne Kline, Madeena Sultana, Mohammad Noaeen, Elvira Nurmambetova, Filipe Lucini, Majed Al-Jefri, and Joon Lee

Contents

1 Supplementary Note 1: Search Strategy Details **1**
1.1 Search Strings Formation . . . 1
1.1.1 Data collection and preparation . . . 1
1.1.2 Lexical Association and language modelling . . . 2
1.2 QGS . . . 3
1.3 Database Search . . . 4
1.4 Inclusion/Exclusion Criteria . . . 4

2 Supplementary Note 2: Visual Interactive Dashboard **6**
2.1 Chord-Diagram [Authors Collaborations] . . . 6
2.2 Stream Diagram [Surveillance Topics Over Time] . . . 6
2.3 Interactive Tabular Visualization [A Big Picture of the Included Studies] . . . 6
2.4 Expandable Tree [The Hierarchy of Themes/Categories/Sub-Categories] . . . 7

3 Supplementary Note 3: Digital Media Platforms **7**

4 Supplementary Note 4: Applicability Challenges **9**

5 Supplementary References: Included Articles **9**

1 Supplementary Note 1: Search Strategy Details

1.1 Search Strings Formation

To form the search string for automatic database search, we applied the following non-linguistic and linguistic techniques.

Non-linguistic— We conducted a literature review and considered tentative keywords used by the existing reviews that investigated the application of Internet-based data for different aspects of public health surveillance. Also, we drew on our experience of applying social media analysis for public health surveillance and included key terms that we frequently used in our relevant studies.

Linguistic– To enrich our search strings with more context-relevant strings in the context of digital public health surveillance, we implemented the following natural language processing (NLP) techniques.

1.1.1. Data collection and preparation

We imported the `title`, `abstract`, and `discussion/conclusion` of the articles in the QGS set to separate '`.txt`' files and used this corpus as the input to our natural language processing algorithms. Due to the complexity and diversity of natural language content, this data was not immediately ready for analysis. We implemented the following data preparation steps iteratively. Each step performed in the nth and final iteration is described below as well as its evolution throughout our iterations.

Step ❶ Convert text to lowercase: We used this for all three iterations. We removed case sensitivity to ensure we do not analyze a word's capitalization as a separate case than its lower-case counterpart.

Step ❷ Removing numbers and punctuations: We used this for all three iterations. This joined hyphenated words together, rather than separate them.

Step ❸ Removing stop-words: Stop-words are common words that provide no meaning on their own, such as 'the', 'this', and 'could'. In this step, we removed the default set of stop-words in the `tm_map` package for `R`. Moreover, we added a new set of stop words specific to academic publications, such as 'paper', 'research', 'propose', 'section', and 'aim'.

Step ❹ Strip Whitespace: We removed excessive whitespaces such as newlines, double spaces, and tabs.

Step ❺ Stemming: We did this for all three iterations. Stemming is the process of reducing words to their origins by removing suffixes.

The cleaned dataset was used to implement the following methods. The extracted strings using this corpus is presented in Supplementary Figure 1.

1.1.2. Lexical Association and language modelling

The traditional automatic keyword extraction methods (e.g. Term Frequency-Inverse Document Frequency (TF-IDF)[1]) use basic document features such as the frequency of terms and the document length. Using these features, relevant terms can still stay independent of other content-carrying terms in the document, which contributes to overlooking the context surrounding terms when measuring their relevance. This is a weakness shared by all *bag of words* approaches. Thus, to extract the keywords for our automatic database search process, we used `lexical association` between the terms in the cleaned corpus, which quantitatively determine the strength of association between two or more words based on their co-occurrence in a corpus [2]. The intuition behind using *lexical association* is the basic assumption that a context in which a word is used can often influence its meaning [3]. Thus, the words that are highly associated with each other occur together, more often than expected by chance, have a particular function and can be considered lexically associated terms. There is a high chance that these words (together) appear in other contextually relevant documents.

To calculate lexical association (i.e. co-occurrence knowledge), we use statistical language models (LMs), which assign probabilities to sequences of words based on their prior history. Using the *chain rule of probability*, we can decompose the probability of any sequence $w_1^n = (w_1 w_2 ... w_n)$ to:

$$P(w_1 w_2 ... w_n) = \prod_{i=1}^{n} P(w_i | w_1^{i-1}) \tag{1}$$

where $P(w|h)$ assigns a probability to term hw, considering some history h, and word w [4]. An *n-gram* model is a sequence of n words that approximates the probability of each word only to the last $n-1$ words: 'social media' and 'public health' in *'social media analysis for public health surveillance'*, are bi-grams (2-gram). Supplementary Figure 1 shows a directional graph of the bi-grams that we extracted from the QGS corpus. The directions in this graph present the order of the most frequent bi-grams. For

[1] Liu F, Liu F, Liu Y. Automatic keyword extraction for the meeting corpus using a supervised approach and bigram expansion. In 2008 IEEE Spoken Language Technology Workshop 2008 Dec 15 (pp. 181-184). IEEE.
[2] Pecina P. Lexical association measures and collocation extraction. Language resources and evaluation. 2010 Apr 1;44(1-2):137-58.
[3] Harris Z. Mathematical structures of language. Interscience tracts in pure and applied mathematics. 1968.
[4] Jurafsky D. Speech language processing. Pearson Education India; 2000.

example in `google`→`query`, the word `google` comes before the word `query`, when defining the search strings.

Supplementary Table 2 lists the keywords related to each of the surveillance and digital platforms categories and can be formulated as below:

$$\texttt{Health} + t_1 \wedge (t_2 \wedge [w_1 \vee w_2 \vee \ldots \vee w_i]), \text{ where } t_1 \in C_1 \text{ and } t_2, w_i \in C_2.$$

For example, '`surveillance + health + social media`' would be one of the search strings for automatic search.

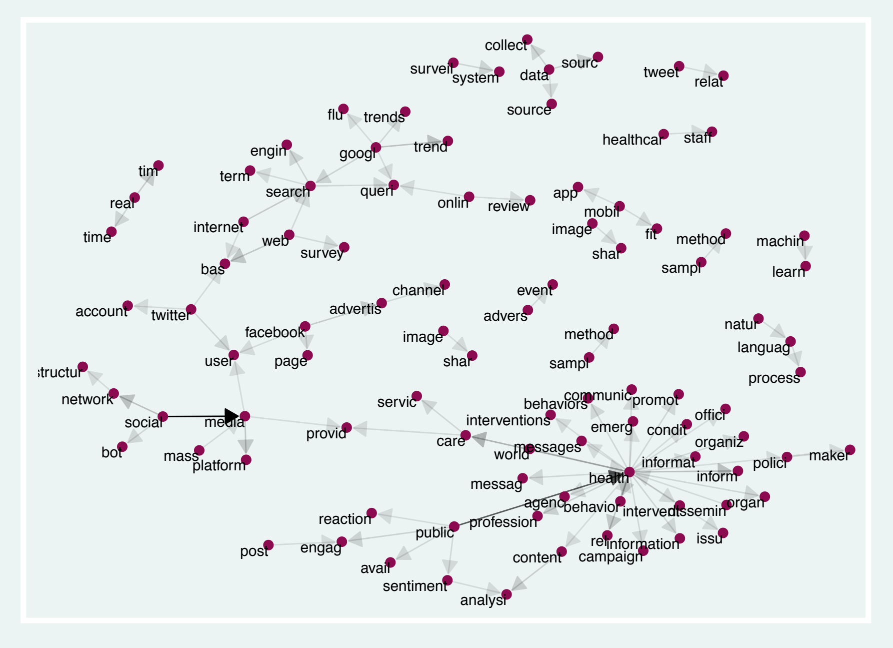

Supplementary Figure 1: Directed graph of common bi-grams formed from the QGS set. These bigrams were used for defining the search strings (filters)

Supplementary Table 1: C_1: Surveillance-related keywords, C_2: Social media-related keywords. λ : empty string, |: or. The terms *infodemiology* and *digital epidemiology* were added to this list for the last round of searches.

C_1	C_2
Surveillance	Social [media, network, listening]
Public	[Facebook, Twitter, Instagram, Yelp, Flicker, Reddit, Quora, YouTube, (micro)blog, Wikipedia, Google+, Google-plus, Tumblr, MySpace, Weibo]
Crowdsource	Web | Internet [λ, forum, search, channel]
Population	Tweet
Community	Google [trends, search, query]
Monitor	Search engine
	Online [review, query, platform]

1.2 QGS

To assess the performance of our search strings in identifying Digital Public Health Surveillance (DPHS) studies in different databases, we created a Quasi Gold Standard (QGS) of 80 related papers. One author manually reviewed all the manuscripts published in the Journal of Medical Internet Research (JMIR)/Public Health and Surveillance (PHS), American Journal of Public Health (APHA), Journal of Public Health (JPH), and Journal of the American Medical Informatics Association (JAMIA) from 2017

to 2018 and selected papers based on their title, abstract, and conclusion. These papers all were used to complement the search strings of the review.

We used the number of records retrieved from database search–using our defined search strings to evaluate the effectiveness of our search process. The sensitivity of this process was defined as follows:[55]

$$\texttt{Sensitivity:}\frac{\texttt{number of QGS articles retrieved}}{\texttt{number of QGS articles indexed in the searched databases}} \quad (2)$$

The searches, using the search strings defined in Supplementary Table 2, in Global Health, Web of Science, PubMed, and Google Scholar retrieved 67 records (before applying the inclusion/exclusion criteria) of the 80 articles in the QGS set. As of these 80 articles were indexed in the searched databases, the precision of our search strings in retrieving the QGS set is 84%. Seven of these records were excluded during the `study selection` phase, which improved the sensitivity to 92%. One reason that our search process could not achieve a sensitivity of 100% is that four papers did not use any of the core terms (i.e. public health, surveillance) in their main text/title and instead, while they utilized digital data to study a public health-related theme in a population. Three papers used other terms such as `infodemiology` or `digital epidemiology` to study the application of digital data for public health surveillance. To improve the sensitivity of the search strings, we added these keywords to our search strings when updated the database search process in January 2020. However, we could not perform any statistical analysis on the updated search results as the QGS set of records was relatively small at 73 records.

Supplementary Table 2: Details of the search process [PHR: Public Health Reports]

**Publisher**	**#QGS$_{(2017-2018)}$**	**#Retrieved**
JMIR/PHS	63	54
APHA	8	6
JPH	2	2
JAMIA	7	5
**Total**	80	67

1.3 Database Search

All databases were searched in April 2019, and the search was updated in September 2019 and January 2020. These searches yielded 4,249 records, of which 2,907 remained after duplicates were removed. A further 2,024 articles were excluded after a title and abstract screen; 503 for surveys, 20 were not written in English, 134 were editorials/letters/viewpoints, 31 used mobile apps to run surveys, and 1,336 were not relevant to the scope. A further 128 articles were removed after the full-text screening based on the exclusion criteria outlined in the methods section. These exclusions left 755 primary articles for the scoping review (Figure 1).

1.4 Inclusion/Exclusion Criteria

We defined the inclusion and exclusion criteria of this review based on the purpose of data generation –by the public and the purpose of data utilization – by potential studies. We detail each of these categories below:

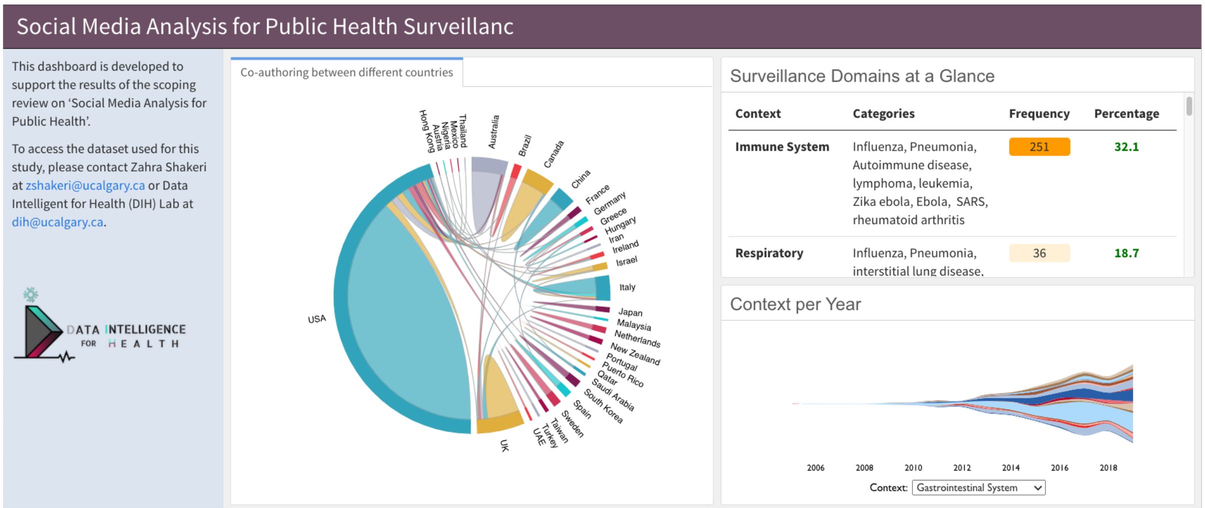

Supplementary Figure 2: The homepage of the visual dashboard presenting the results of the included studies

Data generation purpose– We sought evidence that used data generated voluntarily by the public and is openly accessible by everyone on the Internet. By `voluntary`, we mean the data that is not generated with the primary goal of public health surveillance. Following this definition, all the content sharing and awareness media such as Wikipedia— created and maintained by a community of volunteer editors , news websites, and specific websites— created and maintained for public awareness, without the primary goal of surveillance fall under the scope of our review. In addition to the content generated by laypeople on popular social media platforms (e.g. Twitter, Facebook, Instagram, and Weibo), this definition covers implicit data generated by the public when using different Internet-based technologies. This includes search strings generated by Google Trends, access logs generated by WikiTrends, and likes/clicks generated during user interactions with different digital platforms. We did not put any constraint on the type of data and investigated ten different type combinations in this review. However we **excluded** studies that utilized the following digital data sources:

Online surveys— Studies that utilized the data generated by online surveys/poll were excluded, as this data is not generated voluntarily and might not be openly accessible to everyone. We mean surveys posted on social media platforms or are sent directly to the public by online surveys here.

Mobile App surveys— We excluded all the studies that developed an app to intentionally collect data from their users for the purpose of public health monitoring. Also, studies that used mobile apps to publish the survey link were excluded for further analysis.

Selected social media users— Studies that selected a specific cohort of users on digital platforms for their data collection, while the user was aware of this process and signed consent forms were excluded from this review.

Data utilization purpose– We included studies that used digital data to implement a surveillance system (infoveillance) directly or mined, analyzed, and aggregated information from digital resources to inform public health and public policy for public health surveillance purposes (infodemiology). All the studies that investigated the applicability and the usefulness of different types of digital data for public health surveillance were included in this review. The '`purpose`' of data utilization covers common applications such as outbreak detection, predicting seasonal diseases, explor-

ing unhealthy advertisements, and vaccines to less common applications such as animal health, pediatric health, exploring disease burden, and the risk factors associated with different health practices. We **excluded** studies that utilized digital data for the following purposed:

Education— Studies that leveraged the content of digital media for the purpose of public health-related education (e.g. university students, public health professionals) were excluded for further analysis.

Social media for recruitment— Studies that used social media itself and digital data to recruit subjects for public health studies were excluded.

With only technical contribution— Studies that only contributed to developing a new machine learning or other technical methods for utilizing digital data in public health surveillance were excluded.

Studies were deemed eligible if they met both of the above conditions (i.e. data and purpose). Studies that did not meet any of these conditions were excluded from this review. Also, all review studies that investigated the publications on DPHS were excluded. Also, general commentary, letters, and perspective publications were excluded.

2 Supplementary Note 2: Visual Interactive Dashboard

To complement the results of this review and to add more intuition to the results with a higher level of granularity, we have developed an interactive visual dashboard that is accessible at `https://rpubs.com/zshakeri/dashboard` (Supplementary Figure 2). This dashboard contains four main visual components that are detailed below.

2.1 Chord-Diagram [Authors Collaborations]

We developed a Chord diagram to show the collaboration between authors from different countries. Countries (nodes) are displayed all around a circle and connected with arcs (links). The thickness of the links shows the volume of papers that are published between the countries. Self-links visualize the sing-country publications. Users can interact with this diagram for more information about the number of publications and filter the data for further analysis.

2.2 Stream Diagram [Surveillance Topics Over Time]

A stream diagram is a variation of stacked area charts with a more flexible visual design. We used this diagram to represent the evolution of public health topics over time. Areas are usually displayed around a central axis, and edges are used to give a flowing shape. To filter the dataset based on a specific topic, users can filter the data, and the chart will be updated accordingly.

2.3 Interactive Tabular Visualization [A Big Picture of the Included Studies]

This interactive table illustrates a big picture of the included studies in this review, including the public health topics, frequency, percentage, and temporal trend. Users can interact with this table for more details on each of these variables.

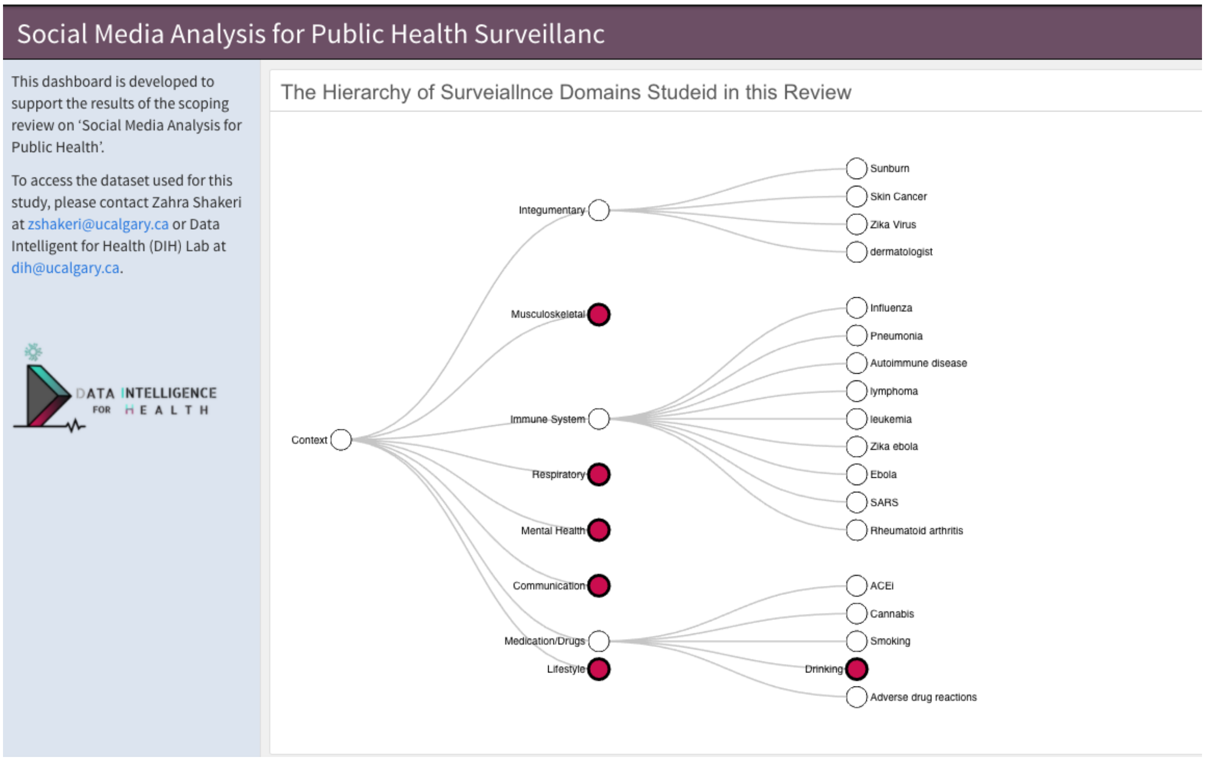

Supplementary Figure 3: Expandable tree diagram, mapped to the PHS topics/categories/sub-categories listed in Table 1.

2.4 Expandable Tree [The Hierarchy of Themes/Categories/Sub-Categories]

Considering the high level of details in Table 1, with a large number of categories and sub-categories, we implemented an interactive collapsible tree diagram that conclusively shows hierarchical data and can be expanded and minimized during the interactions (Supplementary Figure 3). This diagram consists of a root node connected to other nodes by branches. The first level shows the 16 public health topics listed in Table 1. The second level visualizes the 49 categories. The nodes furthest to the right of the tree present the 208 sub-categories and have no child nodes.

3 Supplementary Note 3: Digital Media Platforms

Supplementary Figure 4 represents the frequency of the digital platforms used by the included studies, and the average number of authors per platform. Bing search index, WhatsApp (with one study), Sina, MySpace, Google Reviews, and Google maps (with two studies each) were minimally represented in studies. Regarding the average # of authors per platform, the majority of the platforms follow the overall average (i.e. 5.13), with some exceptions: Sina (9), forums (8), and studies that used more than five digital platforms (social medial platforms) with an average eight authors per study. Supplementary Figure 5 shows the frequency of utilized platforms by the included studies over time. The drop in the number of Twitter studies from 2017 to 2018 could cause by Twitter's new data access policy enacted in 2018; as of July 2018, all the requests for access to Twitter's standard and premium APIs were required to go through a new process. Similarly, the number of Facebook studies spiked dramatically from 2014 to 2017 and then tapered off due to the new Facebook's data collection restrictions implemented in April 2018.

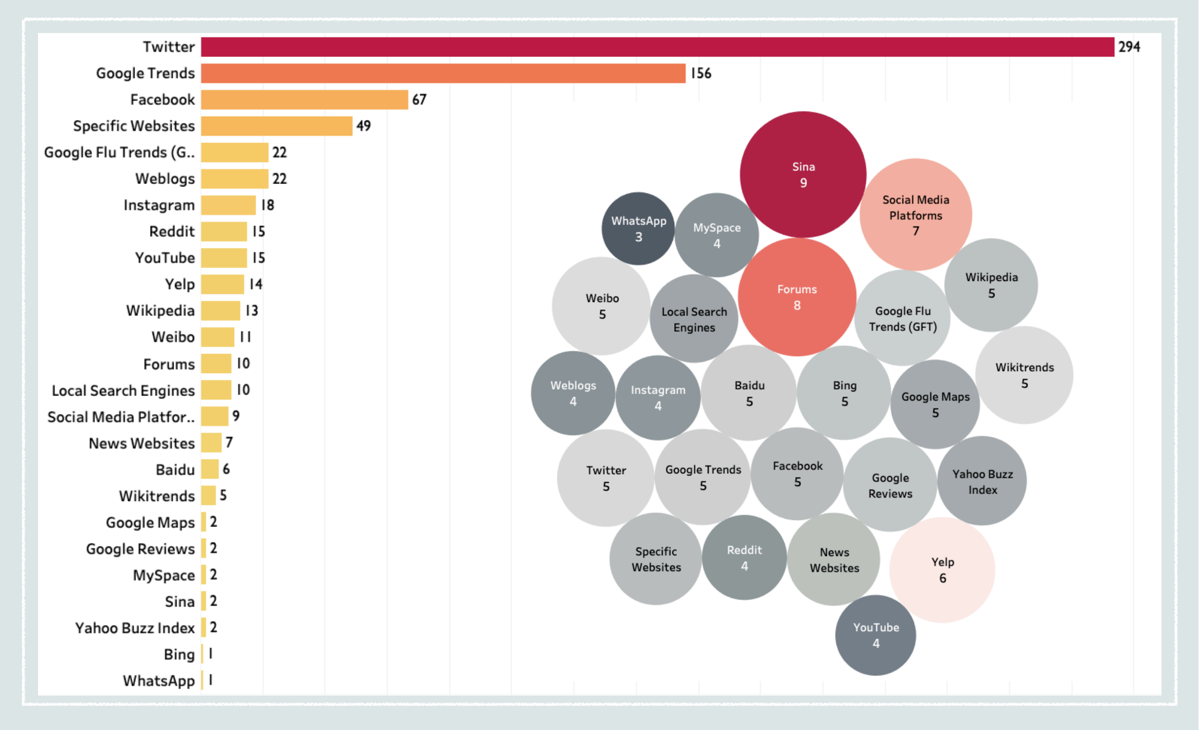

Supplementary Figure 4: The frequency of different digital platforms utilized by the included studies (bar chart), and the average number of authors per platform (bubble chart).

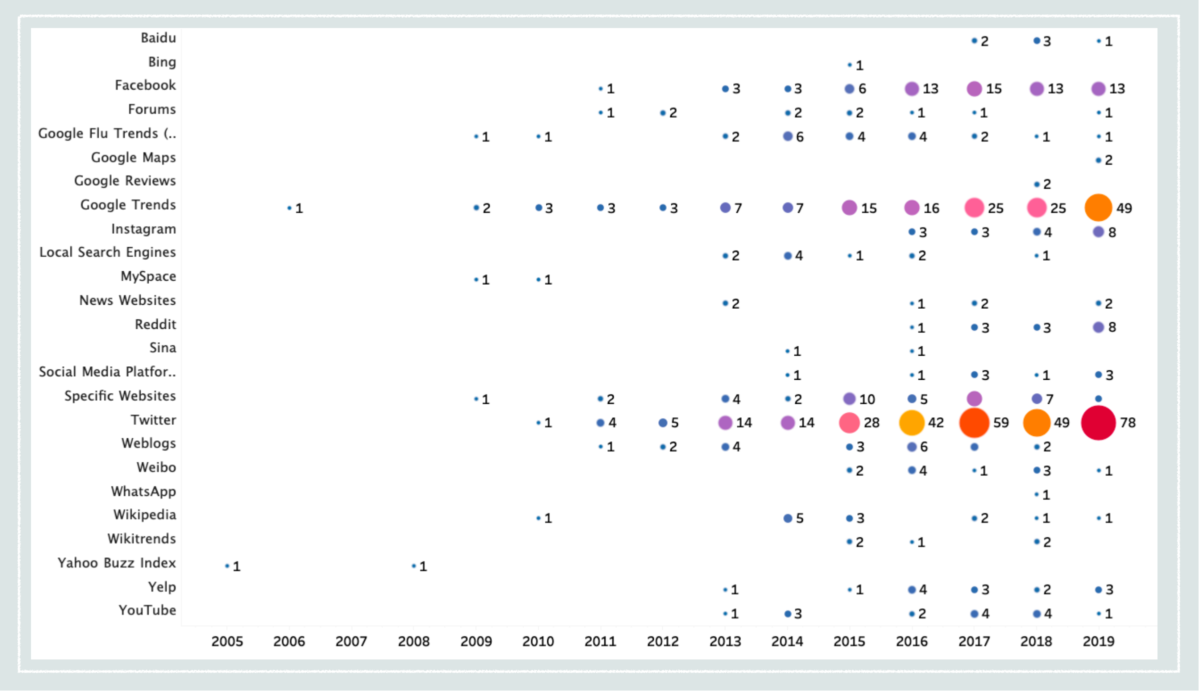

Supplementary Figure 5: The frequency of digital platforms used by the included studies over time.

	Facebook	Google Flu Trends	Google Trends	Local Search Engines	Social Media Platforms	Specific Websites	Twitter	Weblogs	Wikipedia	WikiTrends	YouTube
Communicable Diseases	1	5	4		8	1	2				
Behavioral Risk Factors				1							1
Chronic Diseases	1								1	2	
Mental Health			1				1				
Drug Utilization						1	3		2		
Health Services					2						
Vaccine						2					
Cancer			2		1		2				
Environmental Hazards			1								
Food and Nutrition						1					
Public Health	1						1		1		
Health Practices	1						1				
Pediatric Health								1			

Supplementary Figure 6: The frequency of studies that found the application of digital platforms challenging, mapped to PHS topics.

4 Supplementary Note 4: Applicability Challenges

This section describes the studies that questioned the applicability of using social media for specific purposes. Supplementary Figure 6 shows the mapping between health topics and platforms that might not be effective for them. The number in each cell shows the number of studies associated with each mapping. Interestingly, 52 (7%) studies found the application of digital platforms challenging. For example, Facebook is not an appropriate platform to study the relationship between smoking and genetics, as little information on this topic is present on this platform.[a46] Similarly, the number of views of the Italian Wikipedia articles related to multiple sclerosis (MS) and its treatment showed no promise to explore the disease prevalence,[a232] as this type of data does not reliably reflect its actual epidemiology. While several studies found Twitter as a useful tool to study public interest in and concerns about different diseases, but when it comes to disease comparison (in general), the application of Twitter is more challenging due to the lack of population demographics and word ambiguity.[a753]

The application of specific websites (e.g. news, awareness websites, or online review websites) in developing digital public health campaigns for changing public health-related behaviours was also found to be challenging, which could be due to a poor campaign design or using and inappropriate platform.[a394] The online food review websites in the United States might not be an appropriate platform to detect critical food safety violations in food establishments, as food-borne illnesses are vastly underreported by the US public.[a516]

While Google Trends data is an accurate correlate of the reported incidence of Lyme disease and tick-borne encephalitis in Germany, but it fails to improve the performance of the predictive models.[a408, a428]

5 Supplementary References: Included Articles

In this section, we list all the included studies in this review. These numbers can be directly mapped to Tables 1 and 2 as well as the main text of the manuscript and the Supplementary Information section.

[a1] Jackler, R. K., Li, V. Y., Cardiff, R. A. & Ramamurthi, D. Promotion of tobacco products on facebook: policy versus practice. *Tobacco control* **28**, 67–73 (2019).

[a2] Lee, J. G. Keeping the community posted: Lesbian, gay, bisexual, and transgender blogs and the tobacco epidemic. *LGBT health* **1**, 113–121 (2014).

[a3] Watts, C., Hefler, M. & Freeman, B. 'we have a rich heritage and, we believe, a bright future': how transnational tobacco companies are using twitter to oppose policy and shape their public identity. *Tobacco Control* **28**, 227–232 (2019).

[a4] Cole-Lewis, H. *et al.* Social network behavior and engagement within a smoking cessation facebook page. *Journal of Medical Internet Research* **18**, e205 (2016).

[a5] Ayers, J. W. *et al.* Leveraging big data to improve health awareness campaigns: a novel evaluation of the great american smokeout. *JMIR public health and surveillance* **2**, e16 (2016).

[a6] Jo, C. L., Kornfield, R., Kim, Y., Emery, S. & Ribisl, K. M. Price-related promotions for tobacco products on twitter. *Tobacco control* **25**, 476–479 (2016).

[a7] Cavazos-Rehg, P. A. *et al.* Monitoring of non-cigarette tobacco use using google trends. *Tobacco control* **24**, 249–255 (2015).

[a8] Hefler, M., Kerrigan, V., Freeman, B., Boot, G. R. & Thomas, D. P. Using facebook to reduce smoking among australian aboriginal and torres strait islander people: a participatory grounded action study. *BMC public health* **19**, 615 (2019).

[a9] Park, A., Zhu, S.-H. & Conway, M. The readability of electronic cigarette health information and advice: a quantitative analysis of web-based information. *JMIR public health and surveillance* **3**, e1 (2017).

[a10] Barker, J. O. & Rohde, J. A. Topic clustering of e-cigarette submissions among reddit communities: A network perspective. *Health Education & Behavior* **46**, 59S–68S (2019).

[a11] Chu, K.-H. *et al.* Strategies to find audience segments on twitter for e-cigarette education campaigns. *Addictive behaviors* **91**, 222–226 (2019).

[a12] Guy, M. C. *et al.* Orthodox and unorthodox uses of electronic cigarettes: a surveillance of youtube video content. *Nicotine & Tobacco Research* **21**, 1378–1384 (2019).

[a13] Unger, J. B. *et al.* Perceptions of secondhand e-cigarette aerosol among twitter users. *Tobacco regulatory science* **2**, 146–152 (2016).

[a14] Chu, K.-H. *et al.* Diffusion of messages from an electronic cigarette brand to potential users through twitter. *PloS one* **10**, e0145387 (2015).

[a15] Ayers, J. W. *et al.* Why do people use electronic nicotine delivery systems (electronic cigarettes)? a content analysis of twitter, 2012-2015. *PloS one* **12**, e0170702 (2017).

[a16] Chu, K.-H., Allem, J.-P., Cruz, T. B. & Unger, J. B. Vaping on instagram: cloud chasing, hand checks and product placement. *Tobacco control* **26**, 575–578 (2017).

[a17] Myslín, M., Zhu, S.-H., Chapman, W. & Conway, M. Using twitter to examine smoking behavior and perceptions of emerging tobacco products. *Journal of medical Internet research* **15**, e174 (2013).

[a18] Dai, H., Deem, M. J. & Hao, J. Geographic variations in electronic cigarette advertisements on twitter in the united states. *International journal of public health* **62**, 479–487 (2017).

[a19] Cole-Lewis, H. *et al.* Social listening: a content analysis of e-cigarette discussions on twitter. *Journal of medical Internet research* **17**, e243 (2015).

[a20] Aphinyanaphongs, Y., Lulejian, A., Brown, D. P., Bonneau, R. & Krebs, P. Text classification for automatic detection of e-cigarette use and use for smoking cessation from twitter: a feasibility pilot. In *Biocomputing 2016: Proceedings of the Pacific Symposium*, 480–491 (World Scientific, 2016).

[a21] Harris, J. K. *et al.* Tweeting for and against public health policy: response to the chicago department of public health's electronic cigarette twitter campaign. *Journal of medical Internet research* **16**, e238 (2014).

[a22] Huang, J., Kornfield, R., Szczypka, G. & Emery, S. L. A cross-sectional examination of marketing of electronic cigarettes on twitter. *Tobacco control* **23**, iii26–iii30 (2014).

[a23] Allem, J.-P. *et al.* Campaigns and counter campaigns: reactions on twitter to e-cigarette education. *Tobacco control* **26**, 226–229 (2017).

[a24] Kim, A., Miano, T., Chew, R., Eggers, M. & Nonnemaker, J. Classification of twitter users who tweet about e-cigarettes. *JMIR public health and surveillance* **3**, e63 (2017).

[a25] Williams, R. S., Derrick, J., Liebman, A. K. & LaFleur, K. Content analysis of e-cigarette products, promotions, prices and claims on internet tobacco vendor websites, 2013–2014. *Tobacco control* **27**, e34–e40 (2018).

[a26] Merianos, A. L., Gittens, O. E. & Mahabee-Gittens, E. M. Depiction of health effects of electronic cigarettes on youtube. *Journal of substance use* **21**, 614–619 (2016).

[a27] Gurram, N., Thomson, G., Wilson, N. & Hoek, J. Electronic cigarette online marketing by new zealand vendors. *The New Zealand Medical Journal (Online)* **132**, 20–33 (2019).

[a28] Allem, J.-P., Ferrara, E., Uppu, S. P., Cruz, T. B. & Unger, J. B. E-cigarette surveillance with social media data: social bots, emerging topics, and trends. *JMIR public health and surveillance* **3**, e98 (2017).

[a29] Majmundar, A., Allem, J.-P., Cruz, T. B. & Unger, J. B. Where do people vape? insights from twitter data. *International journal of environmental research and public health* **16**, 3056 (2019).

[a30] Ayers, J. W. *et al.* Revisiting the rise of electronic nicotine delivery systems using search query surveillance. *American journal of preventive medicine* **50**, e173–e181 (2016).

[a31] Huang, J. *et al.* Vaping versus juuling: how the extraordinary growth and marketing of juul transformed the us retail e-cigarette market. *Tobacco control* **28**, 146–151 (2019).

[a32] Sidani, J. E. *et al.* Juul on twitter: Analyzing tweets about use of a new nicotine delivery system. *Journal of School Health* (2020).

[a33] Malik, A., Li, Y., Karbasian, H., Hamari, J. & Johri, A. Live, love, juul: User and content analysis of twitter posts about juul. *American journal of health behavior* **43**, 326–336 (2019).

[a34] Czaplicki, L. *et al.* Characterising juul-related posts on instagram. *Tobacco Control* tobaccocontrol–2018 (2019).

[a35] Zhan, Y., Zhang, Z., Okamoto, J. M., Zeng, D. D. & Leischow, S. J. Underage juul use patterns: Content analysis of reddit messages. *Journal of medical Internet research* **21**, e13038 (2019).

[a36] Brett, E. I. *et al.* A content analysis of juul discussions on social media: Using reddit to understand patterns and perceptions of juul use. *Drug and alcohol dependence* **194**, 358–362 (2019).

[a37] Step, M. M., Bracken, C. C., Trapl, E. S. & Flocke, S. A. User and content characteristics of public tweets referencing little cigars. *American Journal of Health Behavior* **40**, 38–47 (2016).

[a38] Kostygina, G., Tran, H., Shi, Y., Kim, Y. & Emery, S. 'sweeter than a swisher': amount and themes of little cigar and cigarillo content on twitter. *Tobacco control* **25**, i75–i82 (2016).

[a39] Allem, J.-P., Uppu, S. P., Cruz, T. B. & Unger, J. B. Characterizing swisher little cigar–related posts on twitter in 2018: Text analysis. *Journal of medical Internet research* **21**, e14398 (2019).

[a40] Richardson, A. & Vallone, D. M. Youtube: a promotional vehicle for little cigars and cigarillos? *Tobacco control* **23**, 21–26 (2014).

[a41] Williams, R. S. & Derrick, J. C. Internet little cigar and cigarillo vendors: Surveillance of sales and marketing practices via website content analysis. *Preventive medicine* **109**, 51–57 (2018).

[a42] Zhang, Y., Allem, J.-P., Unger, J. B. & Cruz, T. B. Automated identification of hookahs (waterpipes) on instagram: an application in feature extraction using convolutional neural network and support vector machine classification. *Journal of medical Internet research* **20**, e10513 (2018).

[a43] Cawkwell, P. B., Lee, L., Weitzman, M. & Sherman, S. E. Tracking hookah bars in new york: utilizing yelp as a powerful public health tool. *JMIR public health and surveillance* **1**, e19 (2015).

[a44] Chu, K.-H., Colditz, J., Malik, M., Yates, T. & Primack, B. Identifying key target audiences for public health campaigns: Leveraging machine learning in the case of hookah tobacco smoking. *Journal of medical Internet research* **21**, e12443 (2019).

[a45] Ben Taleb, Z., Laestadius, L. I., Asfar, T., Primack, B. A. & Maziak, W. # hookahlife: The rise of waterpipe promotion on instagram. *Health Education & Behavior* **46**, 106–113 (2019).

[a46] De Viron, S., Suggs, L. S., Brand, A. & Van Oyen, H. Communicating genetics and smoking through social media: are we there yet? *Journal of medical Internet research* **15**, e198 (2013).

[a47] Asfar, T. *et al.* How do waterpipe smoking establishments attract smokers? implications for policy. *Substance use & misuse* **54**, 560–571 (2019).

[a48] Salloum, R. G., Osman, A., Maziak, W. & Thrasher, J. F. How popular is waterpipe tobacco smoking? findings from internet search queries. *Tobacco control* **24**, 509–513 (2015).

[a49] Caputi, T. L., Leas, E., Dredze, M., Cohen, J. E. & Ayers, J. W. They're heating up: Internet search query trends reveal significant public interest in heat-not-burn tobacco products. *PloS one* **12**, e0185735 (2017).

[a50] Tabuchi, T. *et al.* Heat-not-burn tobacco product use in japan: its prevalence, predictors and perceived symptoms from exposure to secondhand heat-not-burn tobacco aerosol. *Tobacco control* **27**, e25–e33 (2018).

[a51] Li, Q. *et al.* Understanding users' vaping experiences from social media: Initial study using sentiment opinion summarization techniques. *Journal of medical Internet research* **20**, e252 (2018).

[a52] Allem, J.-P., Majmundar, A., Dharmapuri, L., Cruz, T. B. & Unger, J. B. E-liquid-related posts to twitter in 2018: thematic analysis. *Addictive behaviors reports* **10**, 100196 (2019).

[a53] Laestadius, L. I., Wahl, M. M., Pokhrel, P. & Cho, Y. I. From apple to werewolf: A content analysis of marketing for e-liquids on instagram. *Addictive behaviors* **91**, 119–127 (2019).

[a54] Lima, M. L., Marques, S., Muiños, G. & Camilo, C. All you need is facebook friends? associations between online and face-to-face friendships and health. *Frontiers in psychology* **8**, 68 (2017).

[a55] Nguyen, Q. C. *et al.* Leveraging geotagged twitter data to examine neighborhood happiness, diet, and physical activity. *Applied Geography* **73**, 77–88 (2016).

[a56] Carr, L. J. & Dunsiger, S. I. Search query data to monitor interest in behavior change: application for public health (2012).

[a57] Ford, M. T., Jebb, A. T., Tay, L. & Diener, E. Internet searches for affect-related terms: An indicator of subjective well-being and predictor of health outcomes across us states and metro areas. *Applied Psychology: Health and Well-Being* **10**, 3–29 (2018).

[a58] Yom-Tov, E., Borsa, D., Hayward, A. C., McKendry, R. A. & Cox, I. J. Automatic identification of web-based risk markers for health events. *Journal of medical Internet research* **17**, e29 (2015).

[a59] Kite, J., Foley, B. C., Grunseit, A. C. & Freeman, B. Please like me: Facebook and public health communication. *PloS one* **11**, e0162765 (2016).

[a60] Liu, S., Zhu, M. & Young, S. D. Monitoring freshman college experience through content analysis of tweets: observational study. *JMIR public health and surveillance* **4**, e5 (2018).

[a61] Gittelman, S. *et al.* A new source of data for public health surveillance: Facebook likes. *Journal of medical Internet research* **17**, e98 (2015).

[a62] Prieto, V. M., Matos, S., Alvarez, M., Cacheda, F. & Oliveira, J. L. Twitter: a good place to detect health conditions. *PloS one* **9**, e86191 (2014).

[a63] Nguyen, Q. *et al.* Social media indicators of the food environment and state health outcomes. *Public Health* **148**, 120–128 (2017).

[a64] Muralidhara, S. & Paul, M. J. # healthy selfies: exploration of health topics on instagram. *JMIR public health and surveillance* **4**, e10150 (2018).

[a65] Holmberg, C., Chaplin, J. E., Hillman, T. & Berg, C. Adolescents' presentation of food in social media: An explorative study. *Appetite* **99**, 121–129 (2016).

[a66] Ramachandran, D. *et al.* Food trends and popular nutrition advice online–implications for public health. *Online Journal of Public Health Informatics* **10** (2018).

[a67] Madden, K. M. The seasonal periodicity of healthy contemplations about exercise and weight loss: ecological correlational study. *JMIR public health and surveillance* **3**, e92 (2017).

[a68] Nguyen, Q. C. *et al.* Geotagged us tweets as predictors of county-level health outcomes, 2015–2016. *American journal of public health* **107**, 1776–1782 (2017).

[a69] Cesare, N., Nguyen, Q. C., Grant, C. & Nsoesie, E. O. Social media captures demographic and regional physical activity. *BMJ open sport & exercise medicine* **5**, e000567 (2019).

[a70] Liu, S., Chen, B. & Kuo, A. Monitoring physical activity levels using twitter data: infodemiology study. *Journal of medical Internet research* **21**, e12394 (2019).

[a71] Jane, B. & Gibson, K. Corporate sponsorship of physical activity promotion programmes: part of the solution or part of the problem? *Journal of Public Health* **40**, 279–288 (2018).

[a72] Leggatt-Cook, C. & Chamberlain, K. Blogging for weight loss: personal accountability, writing selves, and the weight-loss blogosphere. *Sociology of health & illness* **34**, 963–977 (2012).

[a73] Ghaznavi, J. & Taylor, L. D. Bones, body parts, and sex appeal: An analysis of# thinspiration images on popular social media. *Body image* **14**, 54–61 (2015).

[a74] Wang, H.-W. & Chen, D.-R. Economic recession and obesity-related internet search behavior in taiwan: analysis of google trends data. *JMIR public health and surveillance* **4**, e37 (2018).

[a75] Nguyen, Q. C. *et al.* Twitter-derived neighborhood characteristics associated with obesity and diabetes. *Scientific reports* **7**, 1–10 (2017).

[a76] Gibbons, J. *et al.* Twitter-based measures of neighborhood sentiment as predictors of residential population health. *PloS one* **14** (2019).

[a77] Giuffrida, L., Lokys, H. & Klemm, O. Assessing the effect of weather on human outdoor perception using twitter. *International Journal of Biometeorology* 1–12 (2018).

[a78] Lynch, M., Brooks-Cleator, L. A., Giles, A. R. & Rumford, M. H. "rip kfc": Public perceptions of a fast-food restaurant closure. *Ecology of food and nutrition* **58**, 120–141 (2019).

[a79] Shen, Y. *et al.* Using social media to assess the consumer nutrition environment: comparing yelp reviews with a direct observation audit instrument for grocery stores. *Public health nutrition* **22**, 257–264 (2019).

[a80] Präger, M., Kurz, C., Böhm, J., Laxy, M. & Maier, W. Using data from online geocoding services for the assessment of environmental obesogenic factors: a feasibility study. *International journal of health geographics* **18**, 13 (2019).

[a81] Chisholm, E. & O'Sullivan, K. Using twitter to explore (un) healthy housing: Learning from the# characterbuildings campaign in new zealand. *International journal of environmental research and public health* **14**, 1424 (2017).

[a82] Althouse, B. M., Allem, J.-P., Childers, M. A., Dredze, M. & Ayers, J. W. Population health concerns during the united states' great recession. *American journal of preventive medicine* **46**, 166–170 (2014).

[a83] Carrotte, E. R., Prichard, I. & Lim, M. S. C. "fitspiration" on social media: A content analysis of gendered images. *Journal of medical Internet research* **19**, e95 (2017).

[a84] Tiggemann, M., Churches, O., Mitchell, L. & Brown, Z. Tweeting weight loss: A comparison of# thinspiration and# fitspiration communities on twitter. *Body Image* **25**, 133–138 (2018).

[a85] Ingram, D. G., Matthews, C. K. & Plante, D. T. Seasonal trends in sleep-disordered breathing: evidence from internet search engine query data. *Sleep and Breathing* **19**, 79–84 (2015).

[a86] Jamison-Powell, S., Linehan, C., Daley, L., Garbett, A. & Lawson, S. " i can't get no sleep" discussing# insomnia on twitter. In *Proceedings of the SIGCHI Conference on Human Factors in Computing Systems*, 1501–1510 (2012).

[a87] McIver, D. J. *et al.* Characterizing sleep issues using twitter. *Journal of medical Internet research* **17**, e140 (2015).

[a88] Veale, H. J. *et al.* The use of social networking platforms for sexual health promotion: identifying key strategies for successful user engagement. *BMC public health* **15**, 85 (2015).

[a89] Young, S. D., Rivers, C. & Lewis, B. Methods of using real-time social media technologies for detection and remote monitoring of hiv outcomes. *Preventive medicine* **63**, 112–115 (2014).

[a90] Meng, H.-W., Kath, S., Li, D. & Nguyen, Q. C. National substance use patterns on twitter. *PloS one* **12**, e0187691 (2017).

[a91] Maani Hessari, N., van Schalkwyk, M. C., Thomas, S. & Petticrew, M. Alcohol industry csr organisations: what can their twitter activity tell us about their independence and their priorities? a comparative analysis. *International journal of environmental research and public health* **16**, 892 (2019).

[a92] Chan, K., Ho, S. & Lam, T. Infodemiology of alcohol use in hong kong mentioned on blogs: infoveillance study. *Journal of medical internet research* **15**, e192 (2013).

[a93] Maani Hessari, N. & Petticrew, M. What does the alcohol industry mean by 'responsible drinking'? a comparative analysis. *Journal of Public Health* **40**, 90–97 (2018).

[a94] Parackal, M., Parackal, S., Eusebius, S. & Mather, D. The use of facebook advertising for communicating public health messages: a campaign against drinking during pregnancy in new zealand. *JMIR public health and surveillance* **3**, e49 (2017).

[a95] Ranney, M. L. *et al.* Tweet now, see you in the ed later? examining the association between alcohol-related tweets and emergency care visits. *Academic Emergency Medicine* **23**, 831–834 (2016).

[a96] Lim, M. S., Hare, J. D., Carrotte, E. R. & Dietze, P. M. An investigation of strategies used in alcohol brand marketing and alcohol-related health promotion on facebook. *Digital health* **2**, 2055207616647305 (2016).

[a97] Wright, L. A., Golder, S., Balkham, A. & McCambridge, J. Understanding public opinion to the introduction of minimum unit pricing in scotland: a qualitative study using twitter. *BMJ open* **9**, e029690 (2019).

[a98] Litt, D. M. *et al.* # drunktwitter: Examining the relations between alcohol-related twitter content and alcohol willingness and use among underage young adults. *Drug and alcohol dependence* **193**, 75–82 (2018).

[a99] Curtis, B. *et al.* Can twitter be used to predict county excessive alcohol consumption rates? *PloS one* **13** (2018).

[a100] Riordan, B. C., Merrill, J. E. & Ward, R. M. "can't wait to blackout tonight": An analysis of the motives to drink to blackout expressed on twitter. *Alcoholism: clinical and experimental research* **43**, 1769–1776 (2019).

[a101] Weitzman, E. R. *et al.* Online searching and social media to detect alcohol use risk at population scale. *American Journal of Preventive Medicine* **58**, 79–88 (2020).

[a102] Lauckner, C. *et al.* Social media photos of substance use and their relationship to attitudes and behaviors among ethnic and racial minority emerging adult men living in low-income areas. *Journal of adolescence* **77**, 152–162 (2019).

[a103] Petticrew, M. *et al.* Diageo's' stop out of control drinking'campaign in ireland: an analysis. *PloS one* **11** (2016).

[a104] Noel, J. K., Babor, T. F. & Grady, J. J. Advertising content, platform characteristics and the appeal of beer advertising on a social networking site. *Alcohol and Alcoholism* **53**, 619–625 (2018).

[a105] Noel, J. K. & Babor, T. F. Alcohol advertising on facebook and the desire to drink among young adults. *Journal of studies on alcohol and drugs* **79**, 751–760 (2018).

[a106] Laestadius, L. I., Guidry, J. P., Greskoviak, R. & Adams, J. Making "weedish fish": An exploratory analysis of cannabis recipes on pinterest. *Substance use & misuse* **54**, 2191–2197 (2019).

[a107] Shi, S., Brant, A. R., Sabolch, A. & Pollom, E. False news of a cannabis cancer cure. *Cureus* **11** (2019).

[a108] Allem, J.-P., Escobedo, P. & Dharmapuri, L. Cannabis surveillance with twitter data: Emerging topics and social bots. *American Journal of Public Health* **110**, 357–362 (2020).

[a109] van Draanen, J., Krishna, T., Tsang, C. & Liu, S. Keeping up with the times: how national public health and governmental organizations communicate about cannabis on twitter. *Substance abuse treatment, prevention, and policy* **14**, 38 (2019).

[a110] Baumgartner, P. & Peiper, N. Utilizing big data and twitter to discover emergent online communities of cannabis users. *Substance abuse: research and treatment* **11**, 1178221817711425 (2017).

[a111] Meacham, M. C., Paul, M. J. & Ramo, D. E. Understanding emerging forms of cannabis use through an online cannabis community: an analysis of relative post volume and subjective highness ratings. *Drug and alcohol dependence* **188**, 364–369 (2018).

[a112] Bright, S. J., Bishop, B., Kane, R., Marsh, A. & Barratt, M. J. Kronic hysteria: exploring the intersection between australian synthetic cannabis legislation, the media, and drug-related harm. *International Journal of Drug Policy* **24**, 231–237 (2013).

[a113] Daniulaityte, R. *et al.* "when 'bad'is 'good'": identifying personal communication and sentiment in drug-related tweets. *JMIR public health and surveillance* **2**, e162 (2016).

[a114] Daniulaityte, R. *et al.* "retweet to pass the blunt": Analyzing geographic and content features of cannabis-related tweeting across the united states. *Journal of studies on alcohol and drugs* **78**, 910–915 (2017).

[a115] Lamy, F. R. *et al.* "those edibles hit hard": Exploration of twitter data on cannabis edibles in the us. *Drug and alcohol dependence* **164**, 64–70 (2016).

[a116] Daniulaityte, R. *et al.* "time for dabs": Analyzing twitter data on marijuana concentrates across the us. *Drug and alcohol dependence* **155**, 307–311 (2015).

[a117] Fullwood, M. D., Kecojevic, A. & Basch, C. H. Examination of youtube videos related to synthetic cannabinoids. *International journal of adolescent medicine and health* **30** (2016).

[a118] Peiper, N. C. *et al.* Patterns of twitter behavior among networks of cannabis dispensaries in california. *Journal of medical Internet research* **19**, e236 (2017).

[a119] van Draanen, J., Krishna, T., Tsang, C. & Liu, S. Keeping up with the times: how national public health and governmental organizations communicate about cannabis on twitter. *Substance abuse treatment, prevention, and policy* **14**, 38 (2019).

[a120] Cavazos-Rehg, P., Krauss, M., Grucza, R. & Bierut, L. Characterizing the followers and tweets of a marijuana-focused twitter handle. *Journal of medical Internet research* **16**, e157 (2014).

[a121] Dakkak, H., Brown, R., Twynstra, J., Charbonneau, K. & Seabrook, J. The perception of pre-and post-natal marijuana exposure on health outcomes: a content analysis of twitter messages. *Journal of neonatal-perinatal medicine* **11**, 409–415 (2018).

[a122] Montgomery, L. & Yockey, A. Rolling and scrolling: The portrayal of marijuana cigars (blunts) on youtube. *Journal of substance use* **23**, 436–440 (2018).

[a123] Lamy, F. R. *et al.* "you got to love rosin: Solventless dabs, pure, clean, natural medicine." exploring twitter data on emerging trends in rosin tech marijuana concentrates. *Drug and alcohol dependence* **183**, 248–252 (2018).

[a124] Cavazos-Rehg, P. A. *et al.* A content analysis of tweets about high-potency marijuana. *Drug and alcohol dependence* **166**, 100–108 (2016).

[a125] Meacham, M. C., Roh, S., Chang, J. S. & Ramo, D. E. Frequently asked questions about dabbing concentrates in online cannabis community discussion forums. *International Journal of Drug Policy* **74**, 11–17 (2019).

[a126] Kolliakou, A. *et al.* Novel psychoactive substances: An investigation of temporal trends in social media and electronic health records. *European Psychiatry* **38**, 15–21 (2016).

[a127] Hassanpour, S., Tomita, N., DeLise, T., Crosier, B. & Marsch, L. A. Identifying substance use risk based on deep neural networks and instagram social media data. *Neuropsychopharmacology* **44**, 487–494 (2019).

[a128] Modrek, S. & Chakalov, B. The# metoo movement in the united states: text analysis of early twitter conversations. *Journal of medical Internet research* **21**, e13837 (2019).

[a129] Bogen, K. W., Bleiweiss, K. K., Leach, N. R. & Orchowski, L. M. # metoo: Disclosure and response to sexual victimization on twitter. *Journal of interpersonal violence* 0886260519851211 (2019).

[a130] Kaufman, M. R., Dey, D., Crainiceanu, C. & Dredze, M. # metoo and google inquiries into sexual violence: a hashtag campaign can sustain information seeking. *Journal of interpersonal violence* 0886260519868197 (2019).

[a131] McHugh, M. C., Saperstein, S. L. & Gold, R. S. Omg u# cyberbully! an exploration of public discourse about cyberbullying on twitter. *Health Education & Behavior* **46**, 97–105 (2019).

[a132] Danielson, C. M. & Emmers-Sommer, T. M. "she stopped me from killing myself": Bullied bloggers' coping behaviors and support sources. *Health communication* **32**, 977–986 (2017).

[a133] McCauley, H. L., Bonomi, A. E., Maas, M. K., Bogen, K. W. & O'Malley, T. L. # maybehedoesnthityou: Social media underscore the realities of intimate partner violence. *Journal of Women's Health* **27**, 885–891 (2018).

[a134] Rizo, C., Deshpande, A., Ing, A. & Seeman, N. A rapid, web-based method for obtaining patient views on effects and side-effects of antidepressants. *Journal of affective disorders* **130**, 290–293 (2011).

[a135] Teoh, D. *et al.* A cross-sectional review of cervical cancer messages on twitter during cervical cancer awareness month. *Journal of lower genital tract disease* **22**, 8 (2018).

[a136] Le, G. M. *et al.* Perceptions of cervical cancer prevention on twitter uncovered by different sampling strategies. *PloS one* **14**, e0211931 (2019).

[a137] Noar, S. M. *et al.* Can a selfie promote public engagement with skin cancer? *Preventive medicine* **111**, 280–283 (2018).

[a138] Ricklefs, C. A. *et al.* Indoor tanning promotions on social media in six us cities# uvtanning# tanning. *Translational behavioral medicine* **6**, 260–270 (2016).

[a139] Hopkins, Z. H. & Secrest, A. M. Public health implications of google searches for sunscreen, sunburn, skin cancer, and melanoma in the united states. *American Journal of Health Promotion* **33**, 611–615 (2019).

[a140] Gough, A. *et al.* Tweet for behavior change: using social media for the dissemination of public health messages. *JMIR public health and surveillance* **3**, e14 (2017).

[a141] Zhao, Y. *et al.* Exploring lung cancer screening discussions on twitter. *Studies in health technology and informatics* **264**, 2011–2012 (2019).

[a142] Xu, S. *et al.* Leveraging social media to promote public health knowledge: example of cancer awareness via twitter. *JMIR public health and surveillance* **2**, e17 (2016).

[a143] Picanço, L., Biancovilli, P. & Jurberg, C. Beyond the drama: The beautiful life in news feeds on cancer. *Journal of Cancer Education* **33**, 424–428 (2018).

[a144] Hale, T. M., Pathipati, A. S., Zan, S. & Jethwani, K. Representation of health conditions on facebook: content analysis and evaluation of user engagement. *Journal of medical Internet research* **16**, e182 (2014).

[a145] Vasconcellos-Silva, P. R. *et al.* Using google trends data to study public interest in breast cancer screening in brazil: why not a pink february? *JMIR Public Health and Surveillance* **3**, e17 (2017).

[a146] Thackeray, R., Burton, S. H., Giraud-Carrier, C., Rollins, S. & Draper, C. R. Using twitter for breast cancer prevention: an analysis of breast cancer awareness month. *BMC cancer* **13**, 508 (2013).

[a147] Modave, F. *et al.* Understanding perceptions and attitudes in breast cancer discussions on twitter. *Studies in health technology and informatics* **264**, 1293 (2019).

[a148] Himelboim, I. & Han, J. Y. Cancer talk on twitter: community structure and information sources in breast and prostate cancer social networks. *Journal of health communication* **19**, 210–225 (2014).

[a149] Carlos, R., Hall, K., Dalton, V. *et al.* Novel data sources for women's health research: mapping breast screening online information seeking through google trends. *Academic radiology* **21**, 1172–1176 (2014).

[a150] Allen, C. G., Roberts, M., Andersen, B. & Khoury, M. J. Communication about hereditary cancers on social media: a content analysis of tweets about hereditary breast and ovarian cancer and lynch syndrome. *Journal of Cancer Education* **35**, 131–137 (2020).

[a151] Vraga, E. K. *et al.* Cancer and social media: A comparison of traffic about breast cancer, prostate cancer, and other reproductive cancers on twitter and instagram. *Journal of health communication* **23**, 181–189 (2018).

[a152] Mohamad, M. & Kok, H. S. Using google trends data to study public interest in breast cancer screening in malaysia. *Asian Pacific Journal of Cancer Prevention: APJCP* **20**, 1427 (2019).

[a153] Nastasi, A. *et al.* Breast cancer screening and social media: a content analysis of evidence use and guideline opinions on twitter. *Journal of Cancer Education* **33**, 695–702 (2018).

[a154] Gage-Bouchard, E. A., LaValley, S., Mollica, M. & Beaupin, L. K. Cancer communication on social media: examining how cancer caregivers use facebook for cancer-related communication. *Cancer nursing* **40**, 332–338 (2017).

[a155] Herth, N. *et al.* Internet information for patients on cancer diets-an analysis of german websites. *Oncology research and treatment* **39**, 273–281 (2016).

[a156] Kent, E. E. *et al.* "obesity is the new major cause of cancer": connections between obesity and cancer on facebook and twitter. *Journal of Cancer Education* **31**, 453–459 (2016).

[a157] O'Hanlon, C. E. What kills us and what moves us: A comparative discourse analysis of heart disease and breast cancer. *Digital health* **5**, 2055207619844865 (2019).

[a158] Luberto, C. M., Hyland, K. A., Streck, J. M., Temel, B. & Park, E. R. Stigmatic and sympathetic attitudes toward cancer patients who smoke: a qualitative analysis of an online discussion board forum. *Nicotine & Tobacco Research* **18**, 2194–2201 (2016).

[a159] Bravo, C. A. & Hoffman-Goetz, L. Tweeting about prostate and testicular cancers: what are individuals saying in their discussions about the 2013 movember canada campaign? *Journal of Cancer Education* **31**, 559–566 (2016).

[a160] Bian, J. *et al.* Using social media data to understand the impact of promotional information on laypeople's discussions: a case study of lynch syndrome. *Journal of medical Internet research* **19**, e414 (2017).

[a161] Sutton, J. *et al.* Lung cancer messages on twitter: content analysis and evaluation. *Journal of the American College of Radiology* **15**, 210–217 (2018).

[a162] Sato, A., Aramaki, E., Shimamoto, Y., Tanaka, S. & Kawakami, K. Blog posting after lung cancer notification: content analysis of blogs written by patients or their families. *JMIR cancer* **1**, e5 (2015).

[a163] Kwan, Z., Yong, S. S. & Robinson, S. Analysis of internet searches using google trends to measure interest in sun protection and skin cancer in selected south-east asian populations. *Photodermatology, Photoimmunology & Photomedicine* **36**, 83–89 (2020).

[a164] Lee, K. C. *et al.* Transmissibility of the campaign for colorectal cancer awareness in korea among twitter users. *Annals of coloproctology* **32**, 184 (2016).

[a165] Alsyouf, M. *et al.* 'fake news' in urology: evaluating the accuracy of articles shared on social media in genitourinary malignancies. *BJU international* **124**, 701–706 (2019).

[a166] Lenoir, P. *et al.* Raising awareness about cervical cancer using twitter: content analysis of the 2015# smearforsmear campaign. *Journal of medical Internet research* **19**, e344 (2017).

[a167] Foroughi, F., Lam, A. K., Lim, M. S., Saremi, N. & Ahmadvand, A. "googling" for cancer: an infodemiological assessment of online search interests in australia, canada, new zealand, the united kingdom, and the united states. *JMIR cancer* **2**, e5 (2016).

[a168] Cooper, C. P., Mallon, K. P., Leadbetter, S., Pollack, L. A. & Peipins, L. A. Cancer internet search activity on a major search engine, united states 2001-2003. *Journal of medical Internet research* **7**, e36 (2005).

[a169] Phillips, C. A. *et al.* Relationship between state-level google online search volume and cancer incidence in the united states: retrospective study. *Journal of medical Internet research* **20**, e6 (2018).

[a170] Faoury, M., Upile, T. & Patel, N. Using google trends to understand information-seeking behaviour about throat cancer. *The Journal of Laryngology & Otology* **133**, 610–614 (2019).

[a171] Odh, I., Löfving, M. & Klaeson, K. Existential challenges in young people living with a cancer diagnosis. *European Journal of Oncology Nursing* **24**, 54–60 (2016).

[a172] Crook, B. *et al.* Hanging by a thread: exploring the features of nonresponse in an online young adult cancer survivorship support community. *Journal of Cancer Survivorship* **10**, 185–193 (2016).

[a173] Pemmaraju, N. *et al.* Rare cancers and social media: analysis of twitter metrics in the first 2 years of a rare-disease community for myeloproliferative neoplasms on social media—# mpnsm. *Current hematologic malignancy reports* **12**, 598–604 (2017).

[a174] Kim, B. & Gillham, D. Gender differences among young adult cancer patients: A study of blogs. *CIN: Computers, Informatics, Nursing* **33**, 3–9 (2015).

[a175] Huang, X. *et al.* Google as a cancer control tool in queensland. *BMC cancer* **17**, 816 (2017).

[a176] Schootman, M. *et al.* The utility of google trends data to examine interest in cancer screening. *BMJ open* **5**, e006678 (2015).

[a177] Noar, S. M., Ribisl, K. M., Althouse, B. M., Willoughby, J. F. & Ayers, J. W. Using digital surveillance to examine the impact of public figure pancreatic cancer announcements on media and search query outcomes. *Journal of the National Cancer Institute Monographs* **2013**, 188–194 (2013).

[a178] Sinnenberg, L. *et al.* Content analysis of metaphors about hypertension and diabetes on twitter: exploratory mixed-methods study. *JMIR diabetes* **3**, e11177 (2018).

[a179] Harris, J. K., Mart, A., Moreland-Russell, S. & Caburnay, C. A. Diabetes topics associated with engagement on twitter. *Preventing chronic disease* **12** (2015).

[a180] Zhang, Y., He, D. & Sang, Y. Facebook as a platform for health information and communication: a case study of a diabetes group. *Journal of medical systems* **37**, 9942 (2013).

[a181] Gabarron, E., Dorronzoro, E., Rivera-Romero, O. & Wynn, R. Diabetes on twitter: a sentiment analysis. *Journal of diabetes science and technology* **13**, 439–444 (2019).

[a182] Abedin, T. *et al.* Social media as a platform for information about diabetes foot care: a study of facebook groups. *Canadian Journal of Diabetes* **41**, 97–101 (2017).

[a183] Tufts, C. *et al.* Characterizing tweet volume and content about common health conditions across pennsylvania: retrospective analysis. *JMIR public health and surveillance* **4**, e10834 (2018).

[a184] Tkachenko, N. *et al.* Google trends can improve surveillance of type 2 diabetes. *Scientific reports* **7**, 1–10 (2017).

[a185] Beguerisse-Díaz, M., McLennan, A. K., Garduno-Hernández, G., Barahona, M. & Ulijaszek, S. J. The 'who'and 'what'of# diabetes on twitter. *Digital health* **3**, 2055207616688841 (2017).

[a186] Tkachenko, N. *et al.* Google trends can improve surveillance of type 2 diabetes. *Scientific reports* **7**, 1–10 (2017).

[a187] AlQarni, Z. A., Yunus, F. & Househ, M. S. Health information sharing on facebook: an exploratory study on diabetes mellitus. *Journal of infection and public health* **9**, 708–712 (2016).

[a188] Liu, Y., Mei, Q., Hanauer, D. A., Zheng, K. & Lee, J. M. Use of social media in the diabetes community: an exploratory analysis of diabetes-related tweets. *JMIR diabetes* **1**, e4 (2016).

[a189] Tenderich, A., Tenderich, B., Barton, T. & Richards, S. E. What are pwds (people with diabetes) doing online? a netnographic analysis. *Journal of diabetes science and technology* **13**, 187–197 (2019).

[a190] Hanna, K., Sambrook, P., Armfield, J. M. & Brennan, D. S. Exploring and modelling impacts of third molar experience on quality of life: a real-time qualitative study using twitter. *International dental journal* **67**, 272–280 (2017).

[a191] Huh, J., Liu, L. S., Neogi, T., Inkpen, K. & Pratt, W. Health vlogs as social support for chronic illness management. *ACM Transactions on Computer-Human Interaction (TOCHI)* **21**, 1–31 (2014).

[a192] Jimeno-Yepes, A., MacKinlay, A., Han, B. & Chen, Q. Identifying diseases, drugs, and symptoms in twitter. (2015).

[a193] Aguirre, P. E. A. *et al.* Are the internet users concerned about molar incisor hypomineralization? an infoveillance study. *International Journal of Paediatric Dentistry* **30**, 27–34 (2020).

[a194] Ng, J. P., Tarazi, N., Byrne, D. P., Baker, J. F. & McCabe, J. P. Scoliosis and the social media: Facebook as a means of information exchange. *Spine deformity* **5**, 102–108 (2017).

[a195] O'Keeffe, S. T. Summertime blues? a re-examination of the seasonality of web searches for restless legs and leg cramps. *Sleep Medicine* **37**, 119–123 (2017).

[a196] Martinez-Arroyo, G. *et al.* Potential uses of an infodemiology approach for health-care services for rheumatology. *Clinical rheumatology* **38**, 869–876 (2019).

[a197] Strotman, P. K., Novicoff, W. M., Nelson, S. J. & Browne, J. A. Increasing public interest in stem cell injections for osteoarthritis of the hip and knee: a google trends analysis. *The Journal of arthroplasty* **34**, 1053–1057 (2019).

[a198] Derksen, C., Serlachius, A., Petrie, K. J. & Dalbeth, N. "what say ye gout experts?" a content analysis of questions about gout posted on the social news website reddit. *BMC musculoskeletal disorders* **18**, 488 (2017).

[a199] Kang, Y., Wang, Y., Zhang, D. & Zhou, L. The public's opinions on a new school meals policy for childhood obesity prevention in the us: A social media analytics approach. *International Journal of Medical Informatics* **103**, 83–88 (2017).

[a200] Chunara, R., Bouton, L., Ayers, J. W. & Brownstein, J. S. Assessing the online social environment for surveillance of obesity prevalence. *PloS one* **8** (2013).

[a201] Huang, Y., Huang, D. & Nguyen, Q. C. Census tract food tweets and chronic disease outcomes in the us, 2015–2018. *International journal of environmental research and public health* **16**, 975 (2019).

[a202] Staite, E. *et al.* 'diabulima'through the lens of social media: a qualitative review and analysis of online blogs by people with type 1 diabetes mellitus and eating disorders. *Diabetic Medicine* **35**, 1329–1336 (2018).

[a203] Harris, J. K., Moreland-Russell, S., Tabak, R. G., Ruhr, L. R. & Maier, R. C. Communication about childhood obesity on twitter. *American journal of public health* **104**, e62–e69 (2014).

[a204] Wang, T., Brede, M., Ianni, A. & Mentzakis, E. Social interactions in online eating disorder communities: A network perspective. *PloS one* **13** (2018).

[a205] Bosley, J. C. *et al.* Decoding twitter: Surveillance and trends for cardiac arrest and resuscitation communication. *Resuscitation* **84**, 206–212 (2013).

[a206] Shah, S. G. S. & Robinson, I. Patients' perspectives on self-testing of oral anticoagulation therapy: content analysis of patients' internet blogs. *BMC health services research* **11**, 25 (2011).

[a207] Bragazzi, N. L. *et al.* Public health awareness of autoimmune diseases after the death of a celebrity. *Clinical rheumatology* **36**, 1911–1917 (2017).

[a208] Al Mamun, M., Ibrahim, H. M. & Turin, T. C. Peer reviewed: social media in communicating health information: an analysis of facebook groups related to hypertension. *Preventing chronic disease* **12** (2015).

[a209] Kamiński, M., Łoniewski, I., Misera, A. & Marlicz, W. Heartburn-related internet searches and trends of interest across six western countries: A four-year retrospective analysis using google ads keyword planner. *International journal of environmental research and public health* **16**, 4591 (2019).

[a210] Scheres, L., Lijfering, W., Middeldorp, S. & Cannegieter, S. Influence of world thrombosis day on digital information seeking on venous thrombosis: a google trends study. *Journal of Thrombosis and Haemostasis* **14**, 2325–2328 (2016).

[a211] Sinnenberg, L. *et al.* Twitter as a potential data source for cardiovascular disease research. *JAMA cardiology* **1**, 1032–1036 (2016).

[a212] Braunberger, T., Mounessa, J., Rudningen, K., Dunnick, C. A. & Dellavalle, R. P. Global skin diseases on instagram hashtags. *Dermatology online journal* **23** (2017).

[a213] Kardeş, S. Seasonal variation in the internet searches for psoriasis. *Archives of dermatological research* **311**, 461–467 (2019).

[a214] Wu, Q. *et al.* Seasonality and global public interest in psoriasis: an infodemiology study. *Postgraduate medical journal* **96**, 139–143 (2020).

[a215] Zink, A. *et al.* Medical needs and major complaints related to pruritus in germany: a 4-year retrospective analysis using google adwords keyword planner. *Journal of the European Academy of Dermatology and Venereology* **33**, 151–156 (2019).

[a216] Zhao, C.-N., Wu, Q. & Pan, H.-F. Using google trends to investigate global copd awareness. *European Respiratory Journal* **54**, 1901076 (2019).

[a217] Apperson, A. *et al.* Facebook groups on chronic obstructive pulmonary disease: Social media content analysis. *International journal of environmental research and public health* **16**, 3789 (2019).

[a218] Harber, P. & Leroy, G. Insights from twitter about public perceptions of asthma, copd, and exposures. *Journal of occupational and environmental medicine* **61**, 484–490 (2019).

[a219] Mavragani, A., Sampri, A., Sypsa, K. & Tsagarakis, K. P. Integrating smart health in the us health care system: infodemiology study of asthma monitoring in the google era. *JMIR public health and surveillance* **4**, e24 (2018).

[a220] Edo-Osagie, O., Smith, G., Lake, I., Edeghere, O. & De La Iglesia, B. Twitter mining using semi-supervised classification for relevance filtering in syndromic surveillance. *PloS one* **14**, e0210689 (2019).

[a221] Brigo, F. *et al.* Wikipedia and neurological disorders. *Journal of clinical neuroscience* **22**, 1170–1172 (2015).

[a222] Brigo, F. *et al.* Information-seeking behaviour for epilepsy: an infodemiological study of searches for wikipedia articles. *Epileptic Disorders* **17**, 460–466 (2015).

[a223] Brigo, F. & Trinka, E. Google search behavior for status epilepticus. *Epilepsy & behavior* **49**, 146–149 (2015).

[a224] Brigo, F. *et al.* Italian wikipedia and epilepsy: an infodemiological study of online information-seeking behavior. *Epilepsy & Behavior* **81**, 119–122 (2018).

[a225] van Campen, J. S. *et al.* Does saint nicholas provoke seizures? hints from google trends. *Epilepsy & Behavior* **32**, 132–134 (2014).

[a226] Brigo, F. *et al.* Why do people google epilepsy?: An infodemiological study of online behavior for epilepsy-related search terms. *Epilepsy & behavior* **31**, 67–70 (2014).

[a227] Bragazzi, N. L. *et al.* Infodemiology of status epilepticus: A systematic validation of the google trends-based search queries. *Epilepsy & Behavior* **55**, 120–123 (2016).

[a228] Ingram, D. G. & Plante, D. T. Seasonal trends in restless legs symptomatology: evidence from internet search query data. *Sleep medicine* **14**, 1364–1368 (2013).

[a229] McGregor, F. *et al.* Social media use by patients with glaucoma: what can we learn? *Ophthalmic and Physiological Optics* **34**, 46–52 (2014).

[a230] Deiner, M. S., Lietman, T. M., McLeod, S. D., Chodosh, J. & Porco, T. C. Surveillance tools emerging from search engines and social media data for determining eye disease patterns. *JAMA ophthalmology* **134**, 1024–1030 (2016).

[a231] Della Rosa, S. & Sen, F. Health topics on facebook groups: content analysis of posts in multiple sclerosis communities. *Interactive journal of medical research* **8**, e10146 (2019).

[a232] Brigo, F. *et al.* Why do people search wikipedia for information on multiple sclerosis? *Multiple sclerosis and related disorders* **20**, 210–214 (2018).

[a233] Deshpande, A. K., Deshpande, S. B. & O'Brien, C. A study of social media utilization by individuals with tinnitus. *American Journal of Audiology* **27**, 559–569 (2018).

[a234] Plante, D. T. & Ingram, D. G. Seasonal trends in tinnitus symptomatology: evidence from internet search engine query data. *European Archives of Oto-Rhino-Laryngology* **272**, 2807–2813 (2015).

[a235] Hemsley, B. & Palmer, S. R. Two studies on twitter networks and tweet content in relation to amyotrophic lateral sclerosis (als): Conversation, information, and'diary of a daily life'. In *HIC*, 41–47 (2016).

[a236] Haghighi, P. D., Kang, Y.-B., Buchbinder, R., Burstein, F. & Whittle, S. Investigating subjective experience and the influence of weather among individuals with fibromyalgia: a content analysis of twitter. *JMIR public health and surveillance* **3**, e4 (2017).

[a237] Hassid, B. G. *et al.* Using search engine query data to explore the epidemiology of common gastrointestinal symptoms. *Digestive diseases and sciences* **62**, 588–592 (2017).

[a238] Tan, W. K., Muldrew, B., Khan, Z. & Fitzgerald, R. C. A crosssectional analysis of facebook comments to study public perception of a new diagnostic test called the cytosponge. *Diseases of the Esophagus* **32**, doy085 (2019).

[a239] Roccetti, M. *et al.* Attitudes of crohn's disease patients: Infodemiology case study and sentiment analysis of facebook and twitter posts. *JMIR public health and surveillance* **3**, e51 (2017).

[a240] Radin, M. & Sciascia, S. Infodemiology of systemic lupus erythematous using google trends. *Lupus* **26**, 886–889 (2017).

[a241] Wu, G. *et al.* Global public interest in systemic lupus erythematosus: an investigation based on internet search data. *Lupus* **28**, 1435–1440 (2019).

[a242] Sciascia, S. & Radin, M. What can google and wikipedia can tell us about a disease? big data trends analysis in systemic lupus erythematosus. *International Journal of Medical Informatics* **107**, 65–69 (2017).

[a243] Wu, G.-C. *et al.* Leveraging google trends to investigate the global public interest in rheumatoid arthritis. *Rheumatology international* **39**, 1439–1444 (2019).

[a244] Ramadona, A. L., Tozan, Y., Lazuardi, L. & Rocklöv, J. A combination of incidence data and mobility proxies from social media predicts the intra-urban spread of dengue in yogyakarta, indonesia. *PLoS neglected tropical diseases* **13**, e0007298 (2019).

[a245] Samaras, L., García-Barriocanal, E. & Sicilia, M.-A. Syndromic surveillance models using web data: the case of influenza in greece and italy using google trends. *JMIR public health and surveillance* **3**, e90 (2017).

[a246] Şerban, O., Thapen, N., Maginnis, B., Hankin, C. & Foot, V. Real-time processing of social media with sentinel: A syndromic surveillance system incorporating deep learning for health classification. *Information Processing & Management* **56**, 1166–1184 (2019).

[a247] Ahmed, W., Bath, P. A., Sbaffi, L. & Demartini, G. Novel insights into views towards h1n1 during the 2009 pandemic: a thematic analysis of twitter data. *Health Information & Libraries Journal* **36**, 60–72 (2019).

[a248] Xue, H., Bai, Y., Hu, H. & Liang, H. Regional level influenza study based on twitter and machine learning method. *PloS one* **14**, e0215600 (2019).

[a249] Yang, W., Lipsitch, M. & Shaman, J. Inference of seasonal and pandemic influenza transmission dynamics. *Proceedings of the National Academy of Sciences* **112**, 2723–2728 (2015).

[a250] Chorianopoulos, K. & Talvis, K. Flutrack. org: Open-source and linked data for epidemiology. *Health informatics journal* **22**, 962–974 (2016).

[a251] de Lange, M. M. *et al.* Comparison of five influenza surveillance systems during the 2009 pandemic and their association with media attention. *BMC Public Health* **13**, 881 (2013).

[a252] Wilson, N. *et al.* Interpreting "google flu trends" data for pandemic h1n1 influenza: the new zealand experience. *Eurosurveillance* **14**, 19386 (2009).

[a253] Santillana, M., Zhang, D. W., Althouse, B. M. & Ayers, J. W. What can digital disease detection learn from (an external revision to) google flu trends? *American journal of preventive medicine* **47**, 341–347 (2014).

[a254] Olson, D. R., Konty, K. J., Paladini, M., Viboud, C. & Simonsen, L. Reassessing google flu trends data for detection of seasonal and pandemic influenza: a comparative epidemiological study at three geographic scales. *PLoS Comput Biol* **9**, e1003256 (2013).

[a255] Moss, R., Zarebski, A., Dawson, P. & McCaw, J. M. Retrospective forecasting of the 2010–2014 melbourne influenza seasons using multiple surveillance systems. *Epidemiology & Infection* **145**, 156–169 (2017).

[a256] Timpka, T. *et al.* Performance of ehealth data sources in local influenza surveillance: a 5-year open cohort study. *Journal of medical Internet research* **16**, e116 (2014).

[a257] Carneiro, H. A. & Mylonakis, E. Google trends: a web-based tool for real-time surveillance of disease outbreaks. *Clinical infectious diseases* **49**, 1557–1564 (2009).

[a258] Pollett, S. *et al.* Evaluating google flu trends in latin america: important lessons for the next phase of digital disease detection. *Clinical Infectious Diseases* ciw657 (2016).

[a259] Thompson, L., Malik, M., Gumel, A., Strome, T. & Mahmud, S. Emergency department and 'google flu trends' data as syndromic surveillance indicators for seasonal influenza. *Epidemiology & Infection* **142**, 2397–2405 (2014).

[a260] Martin, L. J., Xu, B. & Yasui, Y. Improving google flu trends estimates for the united states through transformation. *PloS one* **9**, e109209 (2014).

[a261] Yang, S., Santillana, M. & Kou, S. C. Accurate estimation of influenza epidemics using google search data via argo. *Proceedings of the National Academy of Sciences* **112**, 14473–14478 (2015).

[a262] Pawelek, K. A., Oeldorf-Hirsch, A. & Rong, L. Modeling the impact of twitter on influenza epidemics. *Mathematical Biosciences & Engineering* **11**, 1337 (2014).

[a263] Tausczik, Y., Faasse, K., Pennebaker, J. W. & Petrie, K. J. Public anxiety and information seeking following the h1n1 outbreak: blogs, newspaper articles, and wikipedia visits. *Health communication* **27**, 179–185 (2012).

[a264] Wakamiya, S., Kawai, Y. & Aramaki, E. Twitter-based influenza detection after flu peak via tweets with indirect information: text mining study. *JMIR public health and surveillance* **4**, e65 (2018).

[a265] Mowery, J. Twitter influenza surveillance: quantifying seasonal misdiagnosis patterns and their impact on surveillance estimates. *Online journal of public health informatics* **8** (2016).

[a266] Kandula, S., Pei, S. & Shaman, J. Improved forecasts of influenza-associated hospitalization rates with google search trends. *Journal of the Royal Society Interface* **16**, 20190080 (2019).

[a267] Priedhorsky, R., Daughton, A. R., Barnard, M., O'Connell, F. & Osthus, D. Estimating influenza incidence using search query deceptiveness and generalized ridge regression. *PLoS computational biology* **15**, e1007165 (2019).

[a268] Signorini, A., Segre, A. M. & Polgreen, P. M. The use of twitter to track levels of disease activity and public concern in the us during the influenza a h1n1 pandemic. *PloS one* **6**, e19467 (2011).

[a269] Aslam, A. A. *et al.* The reliability of tweets as a supplementary method of seasonal influenza surveillance. *Journal of medical Internet research* **16**, e250 (2014).

[a270] Hickmann, K. S. *et al.* Forecasting the 2013–2014 influenza season using wikipedia. *PLoS Comput Biol* **11**, e1004239 (2015).

[a271] Nagel, A. C. *et al.* The complex relationship of realspace events and messages in cyberspace: case study of influenza and pertussis using tweets. *Journal of medical Internet research* **15**, e237 (2013).

[a272] Corley, C. D., Cook, D. J., Mikler, A. R. & Singh, K. P. Text and structural data mining of influenza mentions in web and social media. *International journal of environmental research and public health* **7**, 596–615 (2010).

[a273] Wang, F. *et al.* Regional level influenza study with geo-tagged twitter data. *Journal of medical systems* **40**, 189 (2016).

[a274] Gesualdo, F. *et al.* Influenza-like illness surveillance on twitter through automated learning of naïve language. *PLoS One* **8**, e82489 (2013).

[a275] Lu, F. S., Hattab, M. W., Clemente, C. L., Biggerstaff, M. & Santillana, M. Improved state-level influenza nowcasting in the united states leveraging internet-based data and network approaches. *Nature communications* **10**, 1–10 (2019).

[a276] Domnich, A. *et al.* Age-related differences in the accuracy of web query-based predictions of influenza-like illness. *PLoS One* **10**, e0127754 (2015).

[a277] Guo, P. *et al.* Monitoring seasonal influenza epidemics by using internet search data with an ensemble penalized regression model. *Scientific reports* **7**, 46469 (2017).

[a278] McIver, D. J. & Brownstein, J. S. Wikipedia usage estimates prevalence of influenza-like illness in the united states in near real-time. *PLoS Comput Biol* **10**, e1003581 (2014).

[a279] Polgreen, P. M., Chen, Y., Pennock, D. M., Nelson, F. D. & Weinstein, R. A. Using internet searches for influenza surveillance. *Clinical infectious diseases* **47**, 1443–1448 (2008).

[a280] Yang, S. *et al.* Using electronic health records and internet search information for accurate influenza forecasting. *BMC infectious diseases* **17**, 332 (2017).

[a281] Martin, L. J., Lee, B. & Yasui, Y. Google flu trends in canada: a comparison of digital disease surveillance data with physician consultations and respiratory virus surveillance data, 2010–2014. *Epidemiology & Infection* **144**, 325–332 (2016).

[a282] Klembczyk, J. J. *et al.* Google flu trends spatial variability validated against emergency department influenza-related visits. *Journal of medical Internet research* **18**, e175 (2016).

[a283] Araz, O. M., Bentley, D. & Muelleman, R. L. Using google flu trends data in forecasting influenza-like–illness related ed visits in omaha, nebraska. *The American journal of emergency medicine* **32**, 1016–1023 (2014).

[a284] Kandula, S., Hsu, D. & Shaman, J. Subregional nowcasts of seasonal influenza using search trends. *Journal of medical Internet research* **19**, e370 (2017).

[a285] Davidson, M. W., Haim, D. A. & Radin, J. M. Using networks to combine "big data" and traditional surveillance to improve influenza predictions. *Scientific reports* **5**, 8154 (2015).

[a286] Zhang, Y., Bambrick, H., Mengersen, K., Tong, S. & Hu, W. Using google trends and ambient temperature to predict seasonal influenza outbreaks. *Environment international* **117**, 284–291 (2018).

[a287] Zhao, N., Cao, G., Vanos, J. K. & Vecellio, D. J. The effects of synoptic weather on influenza infection incidences: a retrospective study utilizing digital disease surveillance. *International journal of biometeorology* **62**, 69–84 (2018).

[a288] Valdivia, A. *et al.* Monitoring influenza activity in europe with google flu trends: comparison with the findings of sentinel physician networks–results for 2009-10. *Eurosurveillance* **15**, 19621 (2010).

[a289] Broniatowski, D. A., Paul, M. J. & Dredze, M. National and local influenza surveillance through twitter: an analysis of the 2012-2013 influenza epidemic. *PloS one* **8**, e83672 (2013).

[a290] Xu, Q. *et al.* Forecasting influenza in hong kong with google search queries and statistical model fusion. *PloS one* **12** (2017).

[a291] Ertem, Z., Raymond, D. & Meyers, L. A. Optimal multi-source forecasting of seasonal influenza. *PLoS computational biology* **14**, e1006236 (2018).

[a292] Clemente, L., Lu, F. & Santillana, M. Improved real-time influenza surveillance: Using internet search data in eight latin american countries. *JMIR public health and surveillance* **5**, e12214 (2019).

[a293] Zimmer, C., Leuba, S. I., Yaesoubi, R. & Cohen, T. Use of daily internet search query data improves real-time projections of influenza epidemics. *Journal of The Royal Society Interface* **15**, 20180220 (2018).

[a294] Lu, F. S. *et al.* Accurate influenza monitoring and forecasting using novel internet data streams: a case study in the boston metropolis. *JMIR public health and surveillance* **4**, e4 (2018).

[a295] Allen, C., Tsou, M.-H., Aslam, A., Nagel, A. & Gawron, J.-M. Applying gis and machine learning methods to twitter data for multiscale surveillance of influenza. *PloS one* **11** (2016).

[a296] Bilge, U., Bozkurt, S., Yolcular, B. O. & Ozel, D. Can social web helpoff detect influenza related illnesses in turkey? *Stud Health Technol Inform* **174**, 100–104 (2012).

[a297] Santillana, M. *et al.* Combining search, social media, and traditional data sources to improve influenza surveillance. *PLoS computational biology* **11** (2015).

[a298] Shin, S.-Y. *et al.* Correlation between national influenza surveillance data and search queries from mobile devices and desktops in south korea. *PloS one* **11** (2016).

[a299] Thapen, N., Simmie, D., Hankin, C. & Gillard, J. Defender: detecting and forecasting epidemics using novel data-analytics for enhanced response. *PloS one* **11** (2016).

[a300] Ginsberg, J. *et al.* Detecting influenza epidemics using search engine query data. *Nature* **457**, 1012–1014 (2009).

[a301] Rangarajan, P., Mody, S. K. & Marathe, M. Forecasting dengue and influenza incidences using a sparse representation of google trends, electronic health records, and time series data. *PLoS computational biology* **15** (2019).

[a302] Kagashe, I., Yan, Z. & Suheryani, I. Enhancing seasonal influenza surveillance: topic analysis of widely used medicinal drugs using twitter data. *Journal of medical Internet research* **19**, e315 (2017).

[a303] Woo, H. *et al.* Estimating influenza outbreaks using both search engine query data and social media data in south korea. *Journal of medical Internet research* **18**, e177 (2016).

[a304] Yom-Tov, E., Johansson-Cox, I., Lampos, V. & Hayward, A. C. Estimating the secondary attack rate and serial interval of influenza-like illnesses using social media. *Influenza and other respiratory viruses* **9**, 191–199 (2015).

[a305] Sharpe, J. D., Hopkins, R. S., Cook, R. L. & Striley, C. W. Evaluating google, twitter, and wikipedia as tools for influenza surveillance using bayesian change point analysis: a comparative analysis. *JMIR public health and surveillance* **2**, e161 (2016).

[a306] Moss, R., Zarebski, A., Dawson, P. & McCaw, J. M. Forecasting influenza outbreak dynamics in melbourne from internet search query surveillance data. *Influenza and other respiratory viruses* **10**, 314–323 (2016).

[a307] Woo, H. *et al.* Identification of keywords from twitter and web blog posts to detect influenza epidemics in korea. *Disaster medicine and public health preparedness* **12**, 352–359 (2018).

[a308] Zhang, Y., Yakob, L., Bonsall, M. B. & Hu, W. Predicting seasonal influenza epidemics using cross-hemisphere influenza surveillance data and local internet query data. *Scientific reports* **9**, 1–7 (2019).

[a309] Velardi, P., Stilo, G., Tozzi, A. E. & Gesualdo, F. Twitter mining for fine-grained syndromic surveillance. *Artificial intelligence in medicine* **61**, 153–163 (2014).

[a310] Collier, N., Son, N. T. & Nguyen, N. M. Omg u got flu? analysis of shared health messages for bio-surveillance. *Journal of biomedical semantics* **2**, S9 (2011).

[a311] Santos, J. C. & Matos, S. Analysing twitter and web queries for flu trend prediction. *Theoretical Biology and Medical Modelling* **11**, S6 (2014).

[a312] Alessa, A. & Faezipour, M. Preliminary flu outbreak prediction using twitter posts classification and linear regression with historical centers for disease control and prevention reports: Prediction framework study. *JMIR public health and surveillance* **5**, e12383 (2019).

[a313] Chorianopoulos, K. & Talvis, K. Flutrack. org: Open-source and linked data for epidemiology. *Health informatics journal* **22**, 962–974 (2016).

[a314] Chew, C. & Eysenbach, G. Pandemics in the age of twitter: content analysis of tweets during the 2009 h1n1 outbreak. *PloS one* **5**, e14118 (2010).

[a315] Eysenbach, G. Infodemiology: tracking flu-related searches on the web for syndromic surveillance. In *AMIA annual symposium proceedings*, vol. 2006, 244 (American Medical Informatics Association, 2006).

[a316] Yun, G. W. *et al.* Social media and flu: Media twitter accounts as agenda setters. *International journal of medical informatics* **91**, 67–73 (2016).

[a317] Hashmi, S. La gloria, mexico: the possible origins and response of a worldwide h1n1 flu pandemic in 2009. *American Journal of Disaster Medicine* **8**, 57–64 (2013).

[a318] Baltrusaitis, K. *et al.* Determinants of participants' follow-up and characterization of representativeness in flu near you, a participatory disease surveillance system. *JMIR public health and surveillance* **3**, e18 (2017).

[a319] Wu, C., Kao, S.-C., Shih, C.-H. & Kan, M.-H. Open data mining for taiwan's dengue epidemic. *Acta tropica* **183**, 1–7 (2018).

[a320] Yang, S. *et al.* Advances in using internet searches to track dengue. *PLoS computational biology* **13**, e1005607 (2017).

[a321] Guo, P. *et al.* Can internet search queries be used for dengue fever surveillance in china? *International Journal of Infectious Diseases* **63**, 74–76 (2017).

[a322] Worthen, M., Leonard, T. H., Blair, T. R. & Gupta, N. Experiences of parents caring for infants with rare scalp mass as identified through a disease-specific blog. *The Journal of the American Board of Family Medicine* **28**, 750–758 (2015).

[a323] de Almeida Marques-Toledo, C. *et al.* Dengue prediction by the web: Tweets are a useful tool for estimating and forecasting dengue at country and city level. *PLoS neglected tropical diseases* **11**, e0005729 (2017).

[a324] Gluskin, R. T., Johansson, M. A., Santillana, M. & Brownstein, J. S. Evaluation of internet-based dengue query data: Google dengue trends. *PLoS neglected tropical diseases* **8** (2014).

[a325] Ho, H. T. *et al.* Using google trends to examine the spatio-temporal incidence and behavioral patterns of dengue disease: a case study in metropolitan manila, philippines. *Tropical medicine and infectious disease* **3**, 118 (2018).

[a326] Nsoesie, E. O. *et al.* Social media as a sentinel for disease surveillance: what does sociodemographic status have to do with it? *PLoS currents* **8** (2016).

[a327] Husnayain, A., Fuad, A. & Lazuardi, L. Correlation between google trends on dengue fever and national surveillance report in indonesia. *Global Health Action* **12**, 1552652 (2019).

[a328] Chan, E. H., Sahai, V., Conrad, C. & Brownstein, J. S. Using web search query data to monitor dengue epidemics: a new model for neglected tropical disease surveillance. *PLoS neglected tropical diseases* **5**, e1206 (2011).

[a329] Huang, X. *et al.* Can online self-reports assist in real-time identification of influenza vaccination uptake? a cross-sectional study of influenza vaccine-related tweets in the usa, 2013–2017. *BMJ open* **9**, e024018 (2019).

[a330] Liang, H. *et al.* How did ebola information spread on twitter: broadcasting or viral spreading? *BMC public health* **19**, 438 (2019).

[a331] Liu, K. *et al.* Chinese public attention to the outbreak of ebola in west africa: evidence from the online big data platform. *International journal of environmental research and public health* **13**, 780 (2016).

[a332] Mejía, F. *et al.* Fear on the networks: analyzing the 2014 ebola outbreak. *Revista Panamericana de Salud Pública* **41**, e134 (2018).

[a333] Kellogg, C. *et al.* What consumers say about nursing homes in online reviews. *The Gerontologist* **58**, e273–e280 (2018).

[a334] Odlum, M. & Yoon, S. What can we learn about the ebola outbreak from tweets? *American journal of infection control* **43**, 563–571 (2015).

[a335] Feng, S., Hossain, L., Crawford, J. W. & Bossomaier, T. Quantifying network dynamics and information flow across chinese social media during the african ebola outbreak. *Disaster Medicine and Public Health Preparedness* **12**, 26–37 (2018).

[a336] Roberts, H., Seymour, B., Fish, S. A., Robinson, E. & Zuckerman, E. Digital health communication and global public influence: a study of the ebola epidemic. *Journal of Health Communication* **22**, 51–58 (2017).

[a337] Odlum, M. & Yoon, S. Health information needs and health seeking behavior during the 2014-2016 ebola outbreak: A twitter content analysis. *PLoS currents* **10** (2018).

[a338] Fung, I. C.-H. *et al.* Social media's initial reaction to information and misinformation on ebola, august 2014: facts and rumors. *Public Health Reports* **131**, 461–473 (2016).

[a339] Househ, M. Communicating ebola through social media and electronic news media outlets: A cross-sectional study. *Health informatics journal* **22**, 470–478 (2016).

[a340] Pettey, W., Carter, M., TOTH, D. A., Samore, M. & Gundlapalli, A. Constructing ebola transmission chains from west africa and estimating model parameters using internet sources. *Epidemiology & Infection* **145**, 1993–2002 (2017).

[a341] Wong, R. & Harris, J. K. Geospatial distribution of local health department tweets and online searches about ebola during the 2014 ebola outbreak. *Disaster medicine and public health preparedness* **12**, 287–290 (2018).

[a342] Lazard, A. J., Scheinfeld, E., Bernhardt, J. M., Wilcox, G. B. & Suran, M. Detecting themes of public concern: a text mining analysis of the centers for disease control and prevention's ebola live twitter chat. *American journal of infection control* **43**, 1109–1111 (2015).

[a343] Strekalova, Y. A. Health risk information engagement and amplification on social media: News about an emerging pandemic on facebook. *Health Education & Behavior* **44**, 332–339 (2017).

[a344] Alicino, C. *et al.* Assessing ebola-related web search behaviour: insights and implications from an analytical study of google trends-based query volumes. *Infectious diseases of poverty* **4**, 54 (2015).

[a345] Towers, S. *et al.* Mass media and the contagion of fear: the case of ebola in america. *PloS one* **10**, e0129179 (2015).

[a346] Hossain, L., Kam, D., Kong, F., Wigand, R. & Bossomaier, T. Social media in ebola outbreak. *Epidemiology & Infection* **144**, 2136–2143 (2016).

[a347] Mamidi, R., Miller, M., Banerjee, T., Romine, W. & Sheth, A. Identifying key topics bearing negative sentiment on twitter: insights concerning the 2015-2016 zika epidemic. *JMIR Public Health and Surveillance* **5**, e11036 (2019).

[a348] Daughton, A. R. & Paul, M. J. Identifying protective health behaviors on twitter: observational study of travel advisories and zika virus. *Journal of medical Internet research* **21**, e13090 (2019).

[a349] Vijaykumar, S., Meurzec, R. W., Jayasundar, K., Pagliari, C. & Fernandopulle, Y. What's buzzing on your feed? health authorities' use of facebook to combat zika in singapore. *Journal of the American Medical Informatics Association* **24**, 1155–1159 (2017).

[a350] Gui, X. *et al.* Understanding the patterns of health information dissemination on social media during the zika outbreak. In *AMIA Annual Symposium Proceedings*, vol. 2017, 820 (American Medical Informatics Association, 2017).

[a351] Pruss, D. *et al.* Zika discourse in the americas: A multilingual topic analysis of twitter. *PloS one* **14** (2019).

[a352] McGough, S. F., Brownstein, J. S., Hawkins, J. B. & Santillana, M. Forecasting zika incidence in the 2016 latin america outbreak combining traditional disease surveillance with search, social media, and news report data. *PLoS neglected tropical diseases* **11**, e0005295 (2017).

[a353] Bragazzi, N. L. *et al.* Global reaction to the recent outbreaks of zika virus: Insights from a big data analysis. *PloS one* **12** (2017).

[a354] Sharma, M., Yadav, K., Yadav, N. & Ferdinand, K. C. Zika virus pandemic—analysis of facebook as a social media health information platform. *American journal of infection control* **45**, 301–302 (2017).

[a355] Masri, S. *et al.* Use of twitter data to improve zika virus surveillance in the united states during the 2016 epidemic. *BMC public health* **19**, 761 (2019).

[a356] Glowacki, E. M., Lazard, A. J., Wilcox, G. B., Mackert, M. & Bernhardt, J. M. Identifying the public's concerns and the centers for disease control and prevention's reactions during a health crisis: An analysis of a zika live twitter chat. *American journal of infection control* **44**, 1709–1711 (2016).

[a357] Lehnert, J. D. *et al.* Use of obstetric practice web sites to distribute zika virus information to pregnant women during a zika virus outbreak. *Journal of Public Health Management and Practice* **23**, 608 (2017).

[a358] Adebayo, G., Neumark, Y., Gesser-Edelsburg, A., Ahmad, W. A. & Levine, H. Zika pandemic online trends, incidence and health risk communication: a time trend study. *BMJ global health* **2**, e000296 (2017).

[a359] Barata, G., Shores, K. & Alperin, J. P. Local chatter or international buzz? language differences on posts about zika research on twitter and facebook. *PloS one* **13**, e0190482 (2018).

[a360] Chen, S. *et al.* Dynamics of health agency response and public engagement in public health emergency: A case study of centers for disease control and prevention tweeting patterns during the 2016 zika epidemic .

[a361] Stefanidis, A. *et al.* Zika in twitter: temporal variations of locations, actors, and concepts. *JMIR public health and surveillance* **3**, e22 (2017).

[a362] Seltzer, E., Horst-Martz, E., Lu, M. & Merchant, R. Public sentiment and discourse about zika virus on instagram. *Public Health* **150**, 170–175 (2017).

[a363] Petersen, J., Simons, H., Patel, D. & Freedman, J. Early detection of perceived risk among users of a uk travel health website compared with internet search activity and media coverage during the 2015–2016 zika virus outbreak: an observational study. *BMJ open* **7**, e015831 (2017).

[a364] Teng, Y. *et al.* Dynamic forecasting of zika epidemics using google trends. *PloS one* **12**, e0165085 (2017).

[a365] Majumder, M. S. *et al.* Utilizing nontraditional data sources for near real-time estimation of transmission dynamics during the 2015-2016 colombian zika virus disease outbreak. *JMIR public health and surveillance* **2**, e30 (2016).

[a366] Miller, M., Banerjee, T., Muppalla, R., Romine, W. & Sheth, A. What are people tweeting about zika? an exploratory study concerning its symptoms, treatment, transmission, and prevention. *JMIR public health and surveillance* **3**, e38 (2017).

[a367] Robertson, C. & Yee, L. Avian influenza risk surveillance in north america with online media. *PloS one* **11** (2016).

[a368] Gu, H. *et al.* Importance of internet surveillance in public health emergency control and prevention: evidence from a digital epidemiologic study during avian influenza a h7n9 outbreaks. *Journal of medical Internet research* **16**, e20 (2014).

[a369] Chen, B. *et al.* Does eating chicken feet with pickled peppers cause avian influenza? observational case study on chinese social media during the avian influenza a (h7n9) outbreak. *JMIR Public Health and Surveillance* **4**, e32 (2018).

[a370] Barros, J. M., Duggan, J. & Rebholz-Schuhmann, D. Disease mentions in airport and hospital geolocations expose dominance of news events for disease concerns. *Journal of biomedical semantics* **9**, 18 (2018).

[a371] Edelstein, M., Wallensten, A., Zetterqvist, I. & Hulth, A. Detecting the norovirus season in sweden using search engine data–meeting the needs of hospital infection control teams. *PloS one* **9** (2014).

[a372] Osuka, H., Hall, A. J., Wikswo, M. E., Baker, J. M. & Lopman, B. A. Temporal relationship between healthcare-associated and nonhealthcare-associated norovirus outbreaks and google trends data in the united states. *infection control & hospital epidemiology* **39**, 355–358 (2018).

[a373] Signorini, A., Segre, A. M. & Polgreen, P. M. The use of twitter to track levels of disease activity and public concern in the us during the influenza a h1n1 pandemic. *PloS one* **6**, e19467 (2011).

[a374] Song, J., Song, T. M., Seo, D.-C., Jin, D.-L. & Kim, J. S. Social big data analysis of information spread and perceived infection risk during the 2015 middle east respiratory syndrome outbreak in south korea. *Cyberpsychology, Behavior, and Social Networking* **20**, 22–29 (2017).

[a375] Mahroum, N. *et al.* Public reaction to chikungunya outbreaks in italy—insights from an extensive novel data streams-based structural equation modeling analysis. *PloS one* **13**, e0197337 (2018).

[a376] Rocklöv, J. *et al.* Using big data to monitor the introduction and spread of chikungunya, europe, 2017. *Emerging infectious diseases* **25**, 1041 (2019).

[a377] Nan, Y. & Gao, Y. A machine learning method to monitor china's aids epidemics with data from baidu trends. *PloS one* **13** (2018).

[a378] Nan, Y. & Gao, Y. A machine learning method to monitor china's aids epidemics with data from baidu trends. *PloS one* **13** (2018).

[a379] Li, K. *et al.* Using baidu search engine to monitor aids epidemics inform for targeted intervention of hiv/aids in china. *Scientific reports* **9**, 1–12 (2019).

[a380] Kakalou, C., Lazarus, J. V. & Koutkias, V. Mining social media for perceptions and trends on hiv pre-exposure prophylaxis. *Studies in Health Technology and Informatics, 2019, vol. 264, num., p. 4* (2019).

[a381] Gabarron, E., Lau, A. Y. & Wynn, R. Weekly pattern for online information seeking on hiv-a multi-language study. In *MIE*, 778–782 (2016).

[a382] Ireland, M. E., Chen, Q., Schwartz, H. A., Ungar, L. H. & Albarracin, D. Action tweets linked to reduced county-level hiv prevalence in the united states: Online messages and structural determinants. *AIDS and Behavior* **20**, 1256–1264 (2016).

[a383] Domnich, A. *et al.* Demand-based web surveillance of sexually transmitted infections in russia. *International journal of public health* **59**, 841–849 (2014).

[a384] Ireland, M. E., Schwartz, H. A., Chen, Q., Ungar, L. H. & Albarracín, D. Future-oriented tweets predict lower county-level hiv prevalence in the united states. *Health Psychology* **34**, 1252 (2015).

[a385] Cornelius, J., Kennedy, A. & Wesslen, R. An examination of twitter data to identify risky sexual practices among youth and young adults in botswana. *International journal of environmental research and public health* **16**, 656 (2019).

[a386] Young, S. D. & Zhang, Q. Using search engine big data for predicting new hiv diagnoses. *PloS one* **13**, e0199527 (2018).

[a387] Zheluk, A., Quinn, C., Hercz, D. & Gillespie, J. A. Internet search patterns of human immunodeficiency virus and the digital divide in the russian federation: infoveillance study. *Journal of medical Internet research* **15**, e256 (2013).

[a388] Nielsen, R. C. *et al.* Social media monitoring of discrimination and hiv testing in brazil, 2014–2015. *AIDS and Behavior* **21**, 114–120 (2017).

[a389] Odlum, M., Yoon, S., Broadwell, P., Brewer, R. & Kuang, D. How twitter can support the hiv/aids response to achieve the 2030 eradication goal: in-depth thematic analysis of world aids day tweets. *JMIR public health and surveillance* **4**, e10262 (2018).

[a390] Lopez, C., Krauskopf, E., Villota, C., Burzio, L. & Villegas, J. Cervical cancer, human papillomavirus and vaccines: assessment of the information retrieved from general knowledge websites in chile. *Public health* **148**, 19–24 (2017).

[a391] Johnson, A. K., Mikati, T. & Mehta, S. D. Examining the themes of std-related internet searches to increase specificity of disease forecasting using internet search terms. *Scientific reports* **6**, 36503 (2016).

[a392] Young, S. D., Torrone, E. A., Urata, J. & Aral, S. O. Using search engine data as a tool to predict syphilis. *Epidemiology (Cambridge, Mass.)* **29**, 574 (2018).

[a393] Young, S. D., Mercer, N., Weiss, R. E., Torrone, E. A. & Aral, S. O. Using social media as a tool to predict syphilis. *Preventive medicine* **109**, 58–61 (2018).

[a394] Ross, C. *et al.* Update on stis: Impact of a social media campaign targeting men who have sex with men during an outbreak of syphilis in winnipeg, canada. *Canada Communicable Disease Report* **42**, 45 (2016).

[a395] Generous, N., Fairchild, G., Deshpande, A., Del Valle, S. Y. & Priedhorsky, R. Global disease monitoring and forecasting with wikipedia. *PLoS Comput Biol* **10**, e1003892 (2014).

[a396] Janies, D. *et al.* Syndromic surveillance of infectious diseases meets molecular epidemiology in a workflow and phylogeographic application. *Studies in health technology and informatics* **216**, 766 (2015).

[a397] Deiner, M. S. *et al.* Google searches and detection of conjunctivitis epidemics worldwide. *Ophthalmology* **126**, 1219–1229 (2019).

[a398] Hulth, A. & Rydevik, G. Get well: an automated surveillance system for gaining new epidemiological knowledge. *BMC Public Health* **11**, 252 (2011).

[a399] Kaveh-Yazdy, F. & Zareh-Bidoki, A.-M. Search engines, news wires and digital epidemiology: Presumptions and facts. *International journal of medical informatics* **115**, 53–63 (2018).

[a400] Fung, I. C.-H. *et al.* Chinese social media reaction to information about 42 notifiable infectious diseases. *PLoS One* **10** (2015).

[a401] Basch, C. H., Hillyer, G. C., Garcia, P. & Basch, C. E. Clostridium difficile on youtube: A need for greater focus on prevention. *Journal of infection and public health* **11**, 290–293 (2018).

[a402] Deiner, M. S. *et al.* Clinical age-specific seasonal conjunctivitis patterns and their online detection in twitter, blog, forum, and comment social media posts. *Investigative ophthalmology & visual science* **59**, 910–920 (2018).

[a403] Covolo, L. *et al.* Meningococcal disease in italy: public concern, media coverage and policy change. *BMC public health* **19**, 1061 (2019).

[a404] Santangelo, O. *et al.* Digital epidemiology: assessment of measles infection through google trends mechanism in italy. *Ann Ig* **31**, 385–391 (2019).

[a405] Mollema, L. *et al.* Disease detection or public opinion reflection? content analysis of tweets, other social media, and online newspapers during the measles outbreak in the netherlands in 2013. *Journal of medical Internet research* **17**, e128 (2015).

[a406] Jahanbin, K., Rahmanian, F., Rahmanian, V. & Jahromi, A. S. Application of twitter and web news mining in infectious disease surveillance systems and prospects for public health. *GMS Hygiene and Infection Control* **14** (2019).

[a407] Meadows, C. Z., Tang, L. & Liu, W. Twitter message types, health beliefs, and vaccine attitudes during the 2015 measles outbreak in california. *American journal of infection control* **47**, 1314–1318 (2019).

[a408] Sulyok, M., Richter, H., Sulyok, Z., Kapitány-Fövény, M. & Walker, M. D. Predicting tick-borne encephalitis using google trends. *Ticks and Tick-borne Diseases* **11**, 101306 (2020).

[a409] Rohart, F. *et al.* Disease surveillance based on internet-based linear models: an australian case study of previously unmodeled infection diseases. *Scientific reports* **6**, 38522 (2016).

[a410] Schaible, B. J. *et al.* Twitter conversations and english news media reports on poliomyelitis in five different countries, january 2014 to april 2015. *The Permanente journal* **23** (2019).

[a411] Gesser-Edelsburg, A., Walter, N. & Shir-Raz, Y. The "new public" and the "good ol'press": Evaluating online news sources during the 2013 polio outbreak in israel. *Health communication* **32**, 169–179 (2017).

[a412] Zhao, L. *et al.* Simnest: Social media nested epidemic simulation via online semi-supervised deep learning. In *2015 IEEE International Conference on Data Mining*, 639–648 (IEEE, 2015).

[a413] Giordano, A. *et al.* Assessing seasonal dynamics of guillain-barre syndrome with search engine query data. *Neurological Sciences* **40**, 1015–1018 (2019).

[a414] Verma, M. *et al.* Google search trends predicting disease outbreaks: An analysis from india. *Healthcare informatics research* **24**, 300–308 (2018).

[a415] Zhou, X., Ye, J. & Feng, Y. Tuberculosis surveillance by analyzing google trends. *IEEE transactions on biomedical engineering* **58**, 2247–2254 (2011).

[a416] Huang, D.-C. & Wang, J.-F. Monitoring hand, foot and mouth disease by combining search engine query data and meteorological factors. *Science of The Total Environment* **612**, 1293–1299 (2018).

[a417] Oren, E., Frere, J., Yom-Tov, E. & Yom-Tov, E. Respiratory syncytial virus tracking using internet search engine data. *BMC public health* **18**, 445 (2018).

[a418] Samaras, L., García-Barriocanal, E. & Sicilia, M.-A. Syndromic surveillance models using web data: The case of scarlet fever in the uk. *Informatics for Health and Social Care* **37**, 106–124 (2012).

[a419] Da'ar, O. B., Yunus, F., Hossain, N. M. & Househ, M. Impact of twitter intensity, time, and location on message lapse of bluebird's pursuit of fleas in madagascar. *Journal of Infection and Public Health* **10**, 396–402 (2017).

[a420] Bragazzi, N. L. & Mahroum, N. Google trends predicts present and future plague cases during the plague outbreak in madagascar: Infodemiological study. *JMIR public health and surveillance* **5**, e13142 (2019).

[a421] Bragazzi, N. L. *et al.* Infodemiological data of west-nile virus disease in italy in the study period 2004–2015. *Data in brief* **9**, 839 (2016).

[a422] Watad, A. *et al.* Forecasting the west nile virus in the united states: an extensive novel data streams–based time series analysis and structural equation modeling of related digital searching behavior. *JMIR public health and surveillance* **5**, e9176 (2019).

[a423] Arehart, C. H., David, M. Z. & Dukic, V. tracking us pertussis incidence: correlation of public health surveillance and google search data varies by state. *Scientific Reports* **9**, 1–10 (2019).

[a424] Pollett, S. *et al.* Validating the use of google trends to enhance pertussis surveillance in california. *PLoS currents* **7** (2015).

[a425] Zhang, Y. *et al.* Monitoring pertussis infections using internet search queries. *Scientific reports* **7**, 1–7 (2017).

[a426] Gianfredi, V. *et al.* Monitoring public interest toward pertussis outbreaks: an extensive google trends–based analysis. *Public Health* **165**, 9–15 (2018).

[a427] Saris, K. *et al.* Does online search behavior coincide with candida auris cases? an exploratory study. *Journal of Fungi* **5**, 44 (2019).

[a428] Kapitány-Fövény, M. *et al.* Can google trends data improve forecasting of lyme disease incidence? *Zoonoses and public health* **66**, 101–107 (2019).

[a429] Seifter, A., Schwarzwalder, A., Geis, K. & Aucott, J. The utility of "google trends" for epidemiological research: Lyme disease as an example. *Geospatial health* 135–137 (2010).

[a430] Lantos, P. M. *et al.* Unorthodox alternative therapies marketed to treat lyme disease. *Clinical Infectious Diseases* **60**, 1776–1782 (2015).

[a431] Adawi, M. *et al.* Discrepancies between classic and digital epidemiology in searching for the mayaro virus: preliminary qualitative and quantitative analysis of google trends. *JMIR public health and surveillance* **3**, e93 (2017).

[a432] Ocampo, A. J., Chunara, R. & Brownstein, J. S. Using search queries for malaria surveillance, thailand. *Malaria journal* **12**, 390 (2013).

[a433] Zhou, X. *et al.* Monitoring epidemic alert levels by analyzing internet search volume. *IEEE Transactions on Biomedical Engineering* **60**, 446–452 (2012).

[a434] Chunara, R., Andrews, J. R. & Brownstein, J. S. Social and news media enable estimation of epidemiological patterns early in the 2010 haitian cholera outbreak. *The American journal of tropical medicine and hygiene* **86**, 39–45 (2012).

[a435] Niforatos, J. D. & Pescatore, R. Search engine queries for pediatric fever during" cold and flu" season. *Cureus* **11** (2019).

[a436] Pretorius, K. A., Mackert, M. & Wilcox, G. B. Sudden infant death syndrome and safe sleep on twitter: analysis of influences and themes to guide health promotion efforts. *JMIR pediatrics and parenting* **1**, e10435 (2018).

[a437] Awofeso, N., Al Imam, S. & Ahmed, A. Content analysis of media coverage of childhood obesity topics in uae newspapers and popular social media platforms, 2014-2017. *International Journal of Health Policy and Management* **8**, 81 (2019).

[a438] Golder, S. *et al.* Pharmacoepidemiologic evaluation of birth defects from health-related postings in social media during pregnancy. *Drug Safety* **42**, 389–400 (2019).

[a439] Klein, A. Z., Sarker, A., Cai, H., Weissenbacher, D. & Gonzalez-Hernandez, G. Social media mining for birth defects research: a rule-based, bootstrapping approach to collecting data for rare health-related events on twitter. *Journal of biomedical informatics* **87**, 68–78 (2018).

[a440] Perzel, S. *et al.* Searching the web: a survey on the quality of advice on postnatal sequelae of intrauterine growth restriction and the implication of developmental origins of health and disease. *Journal of developmental origins of health and disease* **8**, 604–612 (2017).

[a441] Siracuse, B. L., Ippolito, J. A., Gibson, P. D. & Beebe, K. S. Hoverboards: a new cause of pediatric morbidity. *Injury* **48**, 1110–1114 (2017).

[a442] Bakker, K. M., Martinez-Bakker, M. E., Helm, B. & Stevenson, T. J. Digital epidemiology reveals global childhood disease seasonality and the effects of immunization. *Proceedings of the National Academy of Sciences* **113**, 6689–6694 (2016).

[a443] Kräenbring, J. *et al.* Accuracy and completeness of drug information in wikipedia: a comparison with standard textbooks of pharmacology. *PloS one* **9**, e106930 (2014).

[a444] Phillips, J., Lam, C. & Palmisano, L. Analysis of the accuracy and readability of herbal supplement information on wikipedia. *Journal of the American Pharmacists Association* **54**, 406–414 (2014).

[a445] Koppen, L., Phillips, J. & Papageorgiou, R. Analysis of reference sources used in drug-related wikipedia articles. *Journal of the Medical Library Association: JMLA* **103**, 140 (2015).

[a446] Hanna, A. & Hanna, L.-A. What, where and when? using google trends and google to investigate patient needs and inform pharmacy practice. *International Journal of Pharmacy Practice* **27**, 80–87 (2019).

[a447] Law, M. R., Mintzes, B. & Morgan, S. G. The sources and popularity of online drug information: an analysis of top search engine results and web page views. *Annals of Pharmacotherapy* **45**, 350–356 (2011).

[a448] Teck, J. T. W. & McCann, M. Tracking internet interest in anabolic-androgenic steroids using google trends. *The International journal on drug policy* **51**, 52 (2018).

[a449] Koo, M. Complementary and alternative medicine on wikipedia: opportunities for improvement. *Evidence-Based Complementary and Alternative Medicine* **2014** (2014).

[a450] Meehan, D., Bizzi, I. H. & Ghezzi, P. Stem cell therapy on the internet: information quality and content analysis of english language web pages returned by google. *Frontiers in ICT* **4**, 28 (2017).

[a451] Elphinston, R. A. *et al.* The promotion of policy changes restricting access to codeine medicines on twitter: what do national pain organizations say? *The Journal of Pain* (2019).

[a452] Kecojevic, A., Basch, C., Basch, C. & Kernan, W. Pre-exposure prophylaxis youtube videos: Content evaluation. *JMIR public health and surveillance* **4**, e19 (2018).

[a453] Callahan, A. *et al.* Analyzing information seeking and drug-safety alert response by health care professionals as new methods for surveillance. *Journal of medical Internet research* **17**, e204 (2015).

[a454] Calix, R. A., Gupta, R., Gupta, M. & Jiang, K. Deep gramulator: Improving precision in the classification of personal health-experience tweets with deep learning. In *2017 IEEE International Conference on Bioinformatics and Biomedicine (BIBM)*, 1154–1159 (IEEE, 2017).

[a455] Carbonell, P., Mayer, M. Á. & Bravo, À. Exploring brand-name drug mentions on twitter for pharmacovigilance. In *MIE*, 55–59 (2015).

[a456] Huesch, M. D. Commercial online social network data and statin side-effect surveillance: a pilot observational study of aggregate mentions on facebook (2017).

[a457] Liang, B. A. & Mackey, T. K. Prevalence and global health implications of social media in direct-to-consumer drug advertising. *Journal of Medical Internet Research* **13**, e64 (2011).

[a458] Mackey, T. K. & Liang, B. A. Global reach of direct-to-consumer advertising using social media for illicit online drug sales. *Journal of medical Internet research* **15**, e105 (2013).

[a459] Mackey, T. K., Kalyanam, J., Katsuki, T. & Lanckriet, G. Twitter-based detection of illegal online sale of prescription opioid. *American journal of public health* **107**, 1910–1915 (2017).

[a460] Jha, S., Wang, Z., Laucis, N. & Bhattacharyya, T. Trends in media reports, oral bisphosphonate prescriptions, and hip fractures 1996–2012: an ecological analysis. *Journal of Bone and Mineral Research* **30**, 2179–2187 (2015).

[a461] Santacroce, R. *et al.* Psyclones: a roller coaster of life? hidden synthetic cannabinoids and stimulants in apparently harmless products. *Human Psychopharmacology: Clinical and Experimental* **30**, 265–271 (2015).

[a462] Sinha, M. S. *et al.* Social media impact of the food and drug administration's drug safety communication messaging about zolpidem: mixed-methods analysis. *JMIR public health and surveillance* **4**, e1 (2018).

[a463] Cumbraos-Sánchez, M. J. *et al.* Qualitative and quantitative evaluation of the use of twitter as a tool of antimicrobial stewardship. *International journal of medical informatics* **131**, 103955 (2019).

[a464] Powell, G. E. *et al.* Social media listening for routine post-marketing safety surveillance. *Drug safety* **39**, 443–454 (2016).

[a465] Odgers, D. J., Harpaz, R., Callahan, A., Stiglic, G. & Shah, N. H. Analyzing search behavior of healthcare professionals for drug safety surveillance. In *Pacific Symposium on Biocomputing Co-Chairs*, 306–317 (World Scientific, 2014).

[a466] Matsuda, S. *et al.* Analysis of patient narratives in disease blogs on the internet: an exploratory study of social pharmacovigilance. *JMIR public health and Surveillance* **3**, e10 (2017).

[a467] Jiang, K. & Zheng, Y. Mining twitter data for potential drug effects. In *International conference on advanced data mining and applications*, 434–443 (Springer, 2013).

[a468] Freifeld, C. C. *et al.* Digital drug safety surveillance: monitoring pharmaceutical products in twitter. *Drug safety* **37**, 343–350 (2014).

[a469] O'Connor, K. *et al.* Pharmacovigilance on twitter? mining tweets for adverse drug reactions. In *AMIA annual symposium proceedings*, vol. 2014, 924 (American Medical Informatics Association, 2014).

[a470] Korkontzelos, I. *et al.* Analysis of the effect of sentiment analysis on extracting adverse drug reactions from tweets and forum posts. *Journal of biomedical informatics* **62**, 148–158 (2016).

[a471] Bollegala, D., Maskell, S., Sloane, R., Hajne, J. & Pirmohamed, M. Causality patterns for detecting adverse drug reactions from social media: text mining approach. *JMIR public health and surveillance* **4**, e51 (2018).

[a472] Chee, B. W., Berlin, R. & Schatz, B. Predicting adverse drug events from personal health messages. In *AMIA Annual Symposium Proceedings*, vol. 2011, 217 (American Medical Informatics Association, 2011).

[a473] Topaz, M. *et al.* Clinicians' reports in electronic health records versus patients' concerns in social media: a pilot study of adverse drug reactions of aspirin and atorvastatin. *Drug safety* **39**, 241–250 (2016).

[a474] MacKinlay, A., Aamer, H. & Yepes, A. J. Detection of adverse drug reactions using medical named entities on twitter. In *AMIA Annual Symposium Proceedings*, vol. 2017, 1215 (American Medical Informatics Association, 2017).

[a475] Nguyen, T. *et al.* Estimation of the prevalence of adverse drug reactions from social media. *International journal of medical informatics* **102**, 130–137 (2017).

[a476] Lardon, J. *et al.* Evaluating twitter as a complementary data source for pharmacovigilance. *Expert opinion on drug safety* **17**, 763–774 (2018).

[a477] Pierce, C. E. *et al.* Evaluation of facebook and twitter monitoring to detect safety signals for medical products: an analysis of recent fda safety alerts. *Drug safety* **40**, 317–331 (2017).

[a478] Katsahian, S. *et al.* Evaluation of internet social networks using net scoring tool: A case study in adverse drug reaction mining. *Studies in health technology and informatics* **210**, 526–530 (2015).

[a479] Koutkias, V. G., Lillo-Le Louët, A. & Jaulent, M.-C. Exploiting heterogeneous publicly available data sources for drug safety surveillance: computational framework and case studies. *Expert opinion on drug safety* **16**, 113–124 (2017).

[a480] Patel, R. *et al.* Frequent discussion of insomnia and weight gain with glucocorticoid therapy: an analysis of twitter posts. *NPJ digital medicine* **1**, 1–7 (2018).

[a481] Adrover, C., Bodnar, T., Huang, Z., Telenti, A. & Salathé, M. Identifying adverse effects of hiv drug treatment and associated sentiments using twitter. *JMIR public health and surveillance* **1**, e7 (2015).

[a482] Correia, R. B., Li, L. & Rocha, L. M. Monitoring potential drug interactions and reactions via network analysis of instagram user timelines. In *Biocomputing 2016: Proceedings of the Pacific Symposium*, 492–503 (World Scientific, 2016).

[a483] Smith, K. *et al.* Methods to compare adverse events in twitter to faers, drug information databases, and systematic reviews: proof of concept with adalimumab. *Drug safety* **41**, 1397–1410 (2018).

[a484] Sampathkumar, H., Chen, X.-w. & Luo, B. Mining adverse drug reactions from online healthcare forums using hidden markov model. *BMC medical informatics and decision making* **14**, 91 (2014).

[a485] Cherian, R., Westbrook, M., Ramo, D. & Sarkar, U. Representations of codeine misuse on instagram: content analysis. *JMIR public health and surveillance* **4**, e22 (2018).

[a486] Choo, E. K. *et al.* The prescription opioid epidemic: Social media responses to the residents' perspective article. *Annals of emergency medicine* **67**, 40–48 (2016).

[a487] Chan, B., Lopez, A. & Sarkar, U. The canary in the coal mine tweets: social media reveals public perceptions of non-medical use of opioids. *PLoS One* **10**, e0135072 (2015).

[a488] D'Agostino, A. R. *et al.* Social networking online to recover from opioid use disorder: a study of community interactions. *Drug and alcohol dependence* **181**, 5–10 (2017).

[a489] Young, S. D., Zheng, K., Chu, L. F. & Humphreys, K. Internet searches for opioids predict future emergency department heroin admissions. *Drug and alcohol dependence* **190**, 166–169 (2018).

[a490] Zheluk, A., Quinn, C. & Meylakhs, P. Internet search and krokodil in the russian federation: an infoveillance study. *Journal of medical Internet research* **16**, e212 (2014).

[a491] Semra, T., Chang Vicky, C., Charles-Antoine, D., Wendy, T. & Do Minh, T. At-a-glance-what can social media tell us about the opioid crisis in canada? *Health promotion and chronic disease prevention in Canada: research, policy and practice* **38**, 263 (2018).

[a492] Vosburg, S. K., Haynes, C., Besharat, A. & Green, J. L. Changes in drug use patterns reported on the web after the introduction of adf oxycontin: findings from the researched abuse, diversion, and addiction-related surveillance (radars) system web monitoring program. *Pharmacoepidemiology and drug safety* **26**, 1044–1052 (2017).

[a493] Chary, M. *et al.* Epidemiology from tweets: estimating misuse of prescription opioids in the usa from social media. *Journal of Medical Toxicology* **13**, 278–286 (2017).

[a494] Katsuki, T., Mackey, T. K. & Cuomo, R. Establishing a link between prescription drug abuse and illicit online pharmacies: analysis of twitter data. *Journal of medical Internet research* **17**, e280 (2015).

[a495] Kalyanam, J., Katsuki, T., Lanckriet, G. R. & Mackey, T. K. Exploring trends of nonmedical use of prescription drugs and polydrug abuse in the twittersphere using unsupervised machine learning. *Addictive behaviors* **65**, 289–295 (2017).

[a496] Mackey, T. K. & Kalyanam, J. Detection of illicit online sales of fentanyls via twitter. *F1000Research* **6** (2017).

[a497] Mimura, W. & Akazawa, M. The association between internet searches and moisturizer prescription in japan: Retrospective observational study. *JMIR public health and surveillance* **5**, e13212 (2019).

[a498] Deluca, P. *et al.* Identifying emerging trends in recreational drug use; outcomes from the psychonaut web mapping project. *Progress in Neuro-Psychopharmacology and Biological Psychiatry* **39**, 221–226 (2012).

[a499] Bersani, F. S. *et al.* Drops of madness? recreational misuse of tropicamide collyrium; early warning alerts from russia and italy. *General Hospital Psychiatry* **35**, 571–573 (2013).

[a500] Hanson, C. L. *et al.* Tweaking and tweeting: exploring twitter for nonmedical use of a psychostimulant drug (adderall) among college students. *Journal of medical Internet research* **15**, e62 (2013).

[a501] Anderson, L. S. *et al.* Using social listening data to monitor misuse and nonmedical use of bupropion: a content analysis. *JMIR public health and surveillance* **3**, e6 (2017).

[a502] Yin, S. & Ho, M. Monitoring a toxicological outbreak using internet search query data. *Clinical toxicology* **50**, 818–822 (2012).

[a503] Borron, S. W. *et al.* Intentional misuse and abuse of loperamide: a new look at a drug with "low abuse potential". *The Journal of Emergency Medicine* **53**, 73–84 (2017).

[a504] Akay, A., Dragomir, A. & Erlandsson, B.-E. A novel data-mining approach leveraging social media to monitor consumer opinion of sitagliptin. *IEEE journal of biomedical and health informatics* **19**, 389–396 (2013).

[a505] Rizo, C., Deshpande, A., Ing, A. & Seeman, N. A rapid, web-based method for obtaining patient views on effects and side-effects of antidepressants. *Journal of affective disorders* **130**, 290–293 (2011).

[a506] Graves, R. L. *et al.* Patient narratives in yelp reviews offer insight into opioid experiences and the challenges of pain management. *Pain management* **8**, 95–104 (2018).

[a507] Kate, K., Negi, S. & Kalagnanam, J. Monitoring food safety violation reports from internet forums. In *MIE*, 1090–1094 (2014).

[a508] Quade, P. & Nsoesie, E. O. A platform for crowdsourced foodborne illness surveillance: description of users and reports. *JMIR Public Health and Surveillance* **3**, e42 (2017).

[a509] Vassallo, A. J. *et al.* Junk food marketing on instagram: content analysis. *JMIR public health and surveillance* **4**, e54 (2018).

[a510] Blackburn, K. G., Yilmaz, G. & Boyd, R. L. Food for thought: Exploring how people think and talk about food online. *Appetite* **123**, 390–401 (2018).

[a511] Schomberg, J. P., Haimson, O. L., Hayes, G. R. & Anton-Culver, H. Supplementing public health inspection via social media. *PloS one* **11**, e0152117 (2016).

[a512] Mialon, M. & da Silva Gomes, F. Public health and the ultra-processed food and drink products industry: corporate political activity of major transnationals in latin america and the caribbean. *Public health nutrition* **22**, 1898–1908 (2019).

[a513] Bahk, G. J., Kim, Y. S. & Park, M. S. Use of internet search queries to enhance surveillance of foodborne illness. *Emerging infectious diseases* **21**, 1906 (2015).

[a514] Harris, J. K. *et al.* Using twitter to identify and respond to food poisoning: The food safety stl project. *Journal of Public Health Management and Practice* **23**, 577 (2017).

[a515] Effland, T. *et al.* Discovering foodborne illness in online restaurant reviews. *Journal of the American Medical Informatics Association* **25**, 1586–1592 (2018).

[a516] Harris, J. K., Wong, R., Matthew, M. G. & Mansour, R. Availability of foodborne illness reporting mechanisms for the public on local health department web sites. *Journal of Public Health Management and Practice* **24**, 241–247 (2018).

[a517] Gomez-Lopez, I. N. *et al.* Using social media to identify sources of healthy food in urban neighborhoods. *Journal of Urban Health* **94**, 429–436 (2017).

[a518] Tan, L., Ng, S. H., Omar, A. & Karupaiah, T. What's on youtube? a case study on food and beverage advertising in videos targeted at children on social media. *Childhood Obesity* **14**, 280–290 (2018).

[a519] Cabrera-Hernández, L. M., Wanden-Berghe, C., Castro, C. C. & Sanz-Valero, J. The presence and accuracy of food and nutrition terms in the spanish and english editions of wikipedia: in comparison with the mini larousse encyclopaedia. *Nutricion hospitalaria* **31**, 488–493 (2015).

[a520] Jaichuen, N., Vongmongkol, V., Suphanchaimat, R., Sasiwatpaisit, N. & Tangcharoensathien, V. Food marketing in facebook to thai children and youth: An assessment of the efficacy of thai regulations. *International journal of environmental research and public health* **16**, 1204 (2019).

[a521] Vandevijvere, S., Aitken, C. & Swinburn, B. Volume, nature and potential impact of advertisements on facebook and youtube by food brands popular in new zealand. *To subscribe to the NZMJ, email* **131** (2018).

[a522] Schneider, E. P., McGovern, E. E., Lynch, C. L. & Brown, L. S. Do food blogs serve as a source of nutritionally balanced recipes? an analysis of 6 popular food blogs. *Journal of nutrition education and behavior* **45**, 696–700 (2013).

[a523] Bragg, M. A. *et al.* Fast food, beverage, and snack brands on social media in the united states: An examination of marketing techniques utilized in 2000 brand posts. *Pediatric Obesity* **15**, e12606 (2020).

[a524] Mehta, N., Gupta, A. & Nissan, M. All i have learned, i have learned from google: Why today's facial rejuvenation patients are prone to misinformation, and the steps we can take to contend with unreliable information. *Facial Plastic Surgery* **35**, 387–392 (2019).

[a525] Tang, S. Y., Israel, J. S., Poore, S. O. & Afifi, A. M. Facebook facts: breast reconstruction patient-reported outcomes using social media. *Plastic and reconstructive surgery* **141**, 1106–1113 (2018).

[a526] Seidenberg, A. B. *et al.* Tanning bed burns reported on twitter: over 15,000 in 2013. *Translational behavioral medicine* **6**, 271–276 (2016).

[a527] Toosi, B. & Kalia, S. Seasonal and geographic patterns in tanning using real-time data from google trends. *JAMA dermatology* **152**, 215–217 (2016).

[a528] Mejova, Y., Weber, I. & Fernandez-Luque, L. Online health monitoring using facebook advertisement audience estimates in the united states: evaluation study. *JMIR public health and surveillance* **4**, e30 (2018).

[a529] Paul, M. J. & Dredze, M. Discovering health topics in social media using topic models. *PloS one* **9** (2014).

[a530] Jiang, K., Gupta, R., Gupta, M., Calix, R. A. & Bernard, G. R. Identifying personal health experience tweets with deep neural networks. In *2017 39th Annual International Conference of the IEEE Engineering in Medicine and Biology Society (EMBC)*, 1174–1177 (IEEE, 2017).

[a531] Moon, R. J., Curtis, E. M., Davies, J. H., Cooper, C. & Harvey, N. C. Seasonal variation in internet searches for vitamin d. *Archives of osteoporosis* **12**, 28 (2017).

[a532] Jiang, K. *et al.* Identifying tweets of personal health experience through word embedding and lstm neural network. *BMC bioinformatics* **19**, 210 (2018).

[a533] Hopkins, Z. H., Carlisle, R. & Secrest, A. M. Using google search data for sunburn as a surrogate for sunburn prevalence in the united states. *The Journal of clinical and aesthetic dermatology* **12**, 32 (2019).

[a534] Sabus, C., Johns, B., Schultz, N. & Gagnon, K. Exploration of content and reach of physical therapy-related discussion on twitter. *Physical therapy* **99**, 1048–1055 (2019).

[a535] Ruck, J. M. *et al.* Use of twitter in communicating living solid organ donation information to the public: An exploratory study of living donors and transplant professionals. *Clinical transplantation* **33**, e13447 (2019).

[a536] Linkov, F. *et al.* Bariatric surgery interest around the world: what google trends can teach us. *Surgery for Obesity and Related Diseases* **10**, 533–538 (2014).

[a537] Tijerina, J. D. *et al.* Celebrity influence affecting public interest in plastic surgery procedures: Google trends analysis. *Aesthetic Plastic Surgery* **43**, 1669–1680 (2019).

[a538] Sundstrom, B. *et al.* Voices of the "99 percent": the role of online narrative to improve health care. *The Permanente journal* **20** (2016).

[a539] Li, Y., Cai, X. & Wang, M. Social media ratings of nursing homes associated with experience of care and "nursing home compare" quality measures. *BMC health services research* **19**, 260 (2019).

[a540] Hutchinson, M. & Jackson, D. Troubling fragments and small stories: an analysis of public commentary on nursing through a web blog. *Collegian* **21**, 81–88 (2014).

[a541] Perez, V. & Freedman, S. Do crowdsourced hospital ratings coincide with hospital compare measures of clinical and nonclinical quality? *Health Services Research* **53**, 4491 (2018).

[a542] Bardach, N. S., Asteria-Peñaloza, R., Boscardin, W. J. & Dudley, R. A. The relationship between commercial website ratings and traditional hospital performance measures in the usa. *BMJ Qual Saf* **22**, 194–202 (2013).

[a543] Ranard, B. L. *et al.* Yelp reviews of hospital care can supplement and inform traditional surveys of the patient experience of care. *Health Affairs* **35**, 697–705 (2016).

[a544] Agarwal, A. K. *et al.* Online ratings of the patient experience: emergency departments versus urgent care centers. *Annals of emergency medicine* **73**, 631–638 (2019).

[a545] Kilaru, A. S. *et al.* What do patients say about emergency departments in online reviews? a qualitative study. *BMJ quality & safety* **25**, 14–24 (2016).

[a546] Smith, R. J. & Lipoff, J. B. Evaluation of dermatology practice online reviews: lessons from qualitative analysis. *Jama Dermatology* **152**, 153–157 (2016).

[a547] Dorfman, R. G. *et al.* Happy and unhappy patients: a quantitative analysis of online plastic surgeon reviews for breast augmentation. *Plastic and reconstructive surgery* **141**, 663e–673e (2018).

[a548] Asanad, K. *et al.* Online physician reviews in female pelvic medicine and reconstructive surgery: what do patients really want? *Female pelvic medicine & reconstructive surgery* **24**, 109–114 (2018).

[a549] Gilbert, K. *et al.* Physician rating websites: do radiologists have an online presence? *Journal of the American College of Radiology* **12**, 867–871 (2015).

[a550] Ssendikaddiwa, J. & Lavergne, R. Access to primary care and internet searches for walk-in clinics and emergency departments in canada: Observational study using google trends and population health survey data. *JMIR Public Health and Surveillance* **5**, e13130 (2019).

[a551] Ekström, A., Kurland, L., Farrokhnia, N., Castrén, M. & Nordberg, M. Forecasting emergency department visits using internet data. *Annals of emergency medicine* **65**, 436–442 (2015).

[a552] Tran, N. N. & Lee, J. Online reviews as health data: examining the association between availability of health care services and patient star ratings exemplified by the yelp academic dataset. *JMIR public health and surveillance* **3**, e43 (2017).

[a553] Rogers, M. J., Penvose, I., Curry, E. J., DeGiacomo, A. & Li, X. Medicaid health insurance status limits patient accessibility to rehabilitation services following acl reconstruction surgery. *Orthopaedic journal of sports medicine* **6**, 2325967118763353 (2018).

[a554] Zhang, Z. & Ahmed, W. A comparison of information sharing behaviours across 379 health conditions on twitter. *International journal of public health* **64**, 431–440 (2019).

[a555] Brunner, M., Hemsley, B., Dann, S., Togher, L. & Palmer, S. Hashtag# tbi: a content and network data analysis of tweets about traumatic brain injury. *Brain injury* **32**, 49–63 (2018).

[a556] Berry, N. *et al.* # whywetweetmh: understanding why people use twitter to discuss mental health problems. *Journal of medical Internet research* **19**, e107 (2017).

[a557] Müller, M. M. & Salathé, M. Crowdbreaks: Tracking health trends using public social media data and crowdsourcing. *Frontiers in public health* **7**, 81 (2019).

[a558] Ling, R. & Lee, J. Disease monitoring and health campaign evaluation using google search activities for hiv and aids, stroke, colorectal cancer, and marijuana use in canada: a retrospective observational study. *JMIR public health and surveillance* **2**, e156 (2016).

[a559] Hart, M., Stetten, N., Islam, S. & Pizarro, K. Twitter and public health (part 2): qualitative analysis of how individual health professionals outside organizations use microblogging to promote and disseminate health-related information. *JMIR public health and surveillance* **3**, e54 (2017).

[a560] Schwind, J. S. *et al.* Online surveillance of media health event reporting in nepal: digital disease detection from a one health perspective. *BMC International Health and Human Rights* **17**, 1–7 (2017).

[a561] Wang, T., Wang, F., Xin, X., Pleasants, R. A. & Shi, L. Methods for using microblogs for health communication with a pharmacist-based account. *Patient education and counseling* **99**, 1432–1437 (2016).

[a562] Lee, Y.-F., Yeh, P.-K., Ho, P.-S., Tzeng, D.-S. *et al.* Searching for suicide information on web search engines in chinese. *Journal of Medical Sciences* **37**, 86 (2017).

[a563] Buchbinder, R. & Bourne, A. Content analysis of consumer information about knee arthroscopy in australia. *ANZ journal of surgery* **88**, 346–353 (2018).

[a564] Friedlin, J. & McDonald, C. J. An evaluation of medical knowledge contained in wikipedia and its use in the loinc database. *Journal of the American Medical Informatics Association* **17**, 283–287 (2010).

[a565] Park, A. & Conway, M. Tracking health related discussions on reddit for public health applications. In *AMIA Annual Symposium Proceedings*, vol. 2017, 1362 (American Medical Informatics Association, 2017).

[a566] A Rahim, A. I., Ibrahim, M. I., A Salim, F. N. & Ariffin, M. A. I. Health information engagement factors in malaysia: A content analysis of facebook use by the ministry of health in 2016 and 2017. *International journal of environmental research and public health* **16**, 591 (2019).

[a567] Paul, M. J. & Dredze, M. You are what you tweet: Analyzing twitter for public health. In *Fifth International AAAI Conference on Weblogs and Social Media* (2011).

[a568] Sugawara, Y. *et al.* Cancer patients on twitter: a novel patient community on social media. *BMC Research Notes* **5**, 699 (2012).

[a569] Smith, R. J. *et al.* Variations in facebook posting patterns across validated patient health conditions: a prospective cohort study. *Journal of medical Internet research* **19**, e7 (2017).

[a570] Gage-Bouchard, E. A., LaValley, S., Mollica, M. & Beaupin, L. K. Communication and exchange of specialized health-related support among people with experiential similarity on facebook. *Health communication* **32**, 1233–1240 (2017).

[a571] Tang, C., Zhou, L., Plasek, J., Rozenblum, R. & Bates, D. Comment topic evolution on a cancer institution's facebook page. *Applied clinical informatics* **8**, 854–865 (2017).

[a572] Ure, C., Galpin, A., Cooper-Ryan, A. M. & Condie, J. Charities' use of twitter: exploring social support for women living with and beyond breast cancer. *Information, Communication & Society* **22**, 1062–1079 (2019).

[a573] Wittmeier, K. *et al.* Analysis of a parent-initiated social media campaign for hirschsprung's disease. *Journal of medical Internet research* **16**, e288 (2014).

[a574] Myrick, J. G., Holton, A. E., Himelboim, I. & Love, B. # stupidcancer: exploring a typology of social support and the role of emotional expression in a social media community. *Health communication* **31**, 596–605 (2016).

[a575] Taylor, J. & Pagliari, C. # deathbedlive: the end-of-life trajectory, reflected in a cancer patient's tweets. *BMC palliative care* **17**, 17 (2018).

[a576] Della, L. J., Griffin, D. B., Eroğlu, D., Bernhardt, J. M. & Wells, R. R. Is there health out there in the afrosphere? an analysis of health-related content posted by black bloggers. *Health marketing quarterly* **30**, 1–18 (2013).

[a577] Cole, J., Ezziane, S. & Watkins, C. Rapid creation of an online discussion space (r/nipah) during a serious disease outbreak: Observational study. *JMIR public health and surveillance* **5**, e13753 (2019).

[a578] Marcon, A. R., Bieber, M. & Azad, M. B. Protecting, promoting, and supporting breastfeeding on instagram. *Maternal & child nutrition* **15**, e12658 (2019).

[a579] Prabhu, V. *et al.* Twitter response to the united states preventive services task force recommendations against screening with prostate-specific antigen. *BJU international* **116**, 65–71 (2015).

[a580] King, D. *et al.* Twitter and the health reforms in the english national health service. *Health policy* **110**, 291–297 (2013).

[a581] Merali, H. S., Lin, L.-Y., Li, Q. & Bhalla, K. Using street imagery and crowdsourcing internet marketplaces to measure motorcycle helmet use in bangkok, thailand. *Injury prevention* **26**, 103–108 (2020).

[a582] Simon, T., Goldberg, A., Aharonson-Daniel, L., Leykin, D. & Adini, B. Twitter in the cross fire—the use of social media in the westgate mall terror attack in kenya. *PloS one* **9**, e104136 (2014).

[a583] Khan, Y. *et al.* Comparing twitter data to routine data sources in public health surveillance for the 2015 pan/parapan american games: an ecological study. *Canadian journal of public health* **109**, 419–426 (2018).

[a584] Yom-Tov, E., Borsa, D., Cox, I. J. & McKendry, R. A. Detecting disease outbreaks in mass gatherings using internet data. *Journal of medical Internet research* **16**, e154 (2014).

[a585] Bousquet, J. *et al.* Google trends terms reporting rhinitis and related topics differ in european countries. *Allergy* **72**, 1261–1266 (2017).

[a586] Wakamiya, S., Matsune, S., Okubo, K. & Aramaki, E. Causal relationships among pollen counts, tweet numbers, and patient numbers for seasonal allergic rhinitis surveillance: retrospective analysis. *Journal of medical Internet research* **21**, e10450 (2019).

[a587] Kaidashev, I., Morokhovets, H., Rodinkova, V. & Bousquet, J. Patterns in google trends terms reporting rhinitis and ragweed pollen season in ukraine. *International archives of allergy and immunology* **178**, 363–369 (2019).

[a588] Bousquet, J. *et al.* Google trends and pollen concentrations in allergy and airway diseases in france. *Allergy* **74**, 1910–1919 (2019).

[a589] Kang, M.-G. *et al.* Google unveils a glimpse of allergic rhinitis in the real world. *Allergy* **70**, 124–128 (2015).

[a590] Rong, J., Michalska, S., Subramani, S., Du, J. & Wang, H. Deep learning for pollen allergy surveillance from twitter in australia. *BMC medical informatics and decision making* **19**, 208 (2019).

[a591] Unsal, A. A. *et al.* Doctor google: Correlating internet search trends for epistaxis with metropolitan climates. *American journal of otolaryngology* **40**, 358–363 (2019).

[a592] Yang, F., Wendorf Muhamad, J. & Yang, Q. Exploring environmental health on weibo: A textual analysis of framing haze-related stories on chinese social media. *International journal of environmental research and public health* **16**, 2374 (2019).

[a593] Hswen, Y., Qin, Q., Brownstein, J. S. & Hawkins, J. B. Feasibility of using social media to monitor outdoor air pollution in london, england. *Preventive medicine* **121**, 86–93 (2019).

[a594] Tao, Z., Kokas, A., Zhang, R., Cohan, D. S. & Wallach, D. Inferring atmospheric particulate matter concentrations from chinese social media data. *PloS one* **11**, e0161389 (2016).

[a595] Wang, S., Paul, M. J. & Dredze, M. Social media as a sensor of air quality and public response in china. *Journal of medical Internet research* **17**, e22 (2015).

[a596] Willson, T. J., Lospinoso, J., Weitzel, E. K. & McMains, K. C. Effect of environmental factors on internet searches related to sinusitis. *The Laryngoscope* **125**, 2447–2450 (2015).

[a597] Cecinati, F., Matthews, T., Natarajan, S., McCullen, N. & Coley, D. Mining social media to identify heat waves. *International journal of environmental research and public health* **16**, 762 (2019).

[a598] Green, H. K. *et al.* Google search patterns monitoring the daily health impact of heatwaves in england: How do the findings compare to established syndromic surveillance systems from 2013 to 2017? *Environmental research* **166**, 707–712 (2018).

[a599] Jung, J., Uejio, C. K., Duclos, C. & Jordan, M. Using web data to improve surveillance for heat sensitive health outcomes. *Environmental Health* **18**, 59 (2019).

[a600] Green, H. K. *et al.* Google search patterns monitoring the daily health impact of heatwaves in england: How do the findings compare to established syndromic surveillance systems from 2013 to 2017? *Environmental research* **166**, 707–712 (2018).

[a601] Jung, J. & Uejio, C. K. Social media responses to heat waves. *International journal of biometeorology* **61**, 1247–1260 (2017).

[a602] Mertz, A. & Allukian, M. Community water fluoridation on the internet and social media. *Journal of the Massachusetts Dental Society* **63**, 32–36 (2014).

[a603] Seymour, B., Getman, R., Saraf, A., Zhang, L. H. & Kalenderian, E. When advocacy obscures accuracy online: digital pandemics of public health misinformation through an antifluoride case study. *American journal of public health* **105**, 517–523 (2015).

[a604] Oh, H., Kim, C. & Jeon, J. Public sense of water fluoridation as reflected on twitter 2009–2017. *Journal of Dental Research* **99**, 11–17 (2020).

[a605] Ekenga, C. C., McElwain, C.-A. & Sprague, N. Examining public perceptions about lead in school drinking water: a mixed-methods analysis of twitter response to an environmental health hazard. *International journal of environmental research and public health* **15**, 162 (2018).

[a606] Sharpe, J. D., Bennett, D. M. *et al.* Use of facebook for civilian-led disaster response after a winter storm: A "snowed out atlanta" case study. *Journal of emergency management* **16**, 255–266 (2018).

[a607] Cates, A. L. *et al.* Impact of dual-polarization radar technology and twitter on the hattiesburg, mississippi tornado. *Disaster medicine and public health preparedness* **7**, 585–592 (2013).

[a608] Aoki, T. *et al.* Analysis of the regionality of the number of tweets related to the 2011 fukushima nuclear power station disaster: Content analysis. *JMIR Public Health and Surveillance* **4**, e70 (2018).

[a609] Saha, K. *et al.* A computational study of mental health awareness campaigns on social media. *Translational behavioral medicine* **9**, 1197–1207 (2019).

[a610] Soreni, N., Cameron, D. H., Streiner, D. L., Rowa, K. & McCabe, R. E. Seasonality patterns of internet searches on mental health: exploratory infodemiology study. *JMIR mental health* **6**, e12974 (2019).

[a611] Ayers, J. W., Althouse, B. M., Allem, J.-P., Rosenquist, J. N. & Ford, D. E. Seasonality in seeking mental health information on google. *American journal of preventive medicine* **44**, 520–525 (2013).

[a612] Ambalavan, A. K., Moulahi, B., Azé, J. & Bringay, S. Unveiling online suicide behavior: what can we learn about mental health from suicide survivors of reddit? In *MedInfo*, 50–54 (2019).

[a613] Niederkrotenthaler, T., Till, B. & Garcia, D. Celebrity suicide on twitter: Activity, content and network analysis related to the death of swedish dj tim bergling alias avicii. *Journal of affective disorders* **245**, 848–855 (2019).

[a614] Sumner, S. A. *et al.* Temporal and geographic patterns of social media posts about an emerging suicide game. *Journal of Adolescent Health* **65**, 94–100 (2019).

[a615] Wang, Z., Yu, G., Tian, X., Tang, J. & Yan, X. A study of users with suicidal ideation on sina weibo. *Telemedicine and e-Health* **24**, 702–709 (2018).

[a616] Chang, S.-S., Kwok, S. S. M., Cheng, Q., Yip, P. S. & Chen, Y.-Y. The association of trends in charcoal-burning suicide with google search and newspaper reporting in taiwan: a time series analysis. *Social psychiatry and psychiatric epidemiology* **50**, 1451–1461 (2015).

[a617] Fond, G., Gaman, A., Brunel, L., Haffen, E. & Llorca, P.-M. Google trends®: Ready for real-time suicide prevention or just a zeta-jones effect? an exploratory study. *Psychiatry Research* **228**, 913–917 (2015).

[a618] Chen, P., Chai, J., Zhang, L. & Wang, D. Development and application of a chinese webpage suicide information mining system (sims). *Journal of medical systems* **38**, 88 (2014).

[a619] Solano, P. *et al.* A google-based approach for monitoring suicide risk. *Psychiatry research* **246**, 581–586 (2016).

[a620] Barros, J. M. *et al.* The validity of google trends search volumes for behavioral forecasting of national suicide rates in ireland. *International journal of environmental research and public health* **16**, 3201 (2019).

[a621] McClellan, C., Ali, M. M., Mutter, R., Kroutil, L. & Landwehr, J. Using social media to monitor mental health discussions- evidence from twitter. *Journal of the American Medical Informatics Association* **24**, 496–502 (2017).

[a622] McCarthy, M. J. Internet monitoring of suicide risk in the population. *Journal of affective disorders* **122**, 277–279 (2010).

[a623] Jashinsky, J. *et al.* Tracking suicide risk factors through twitter in the us. *Crisis* (2014).

[a624] Kavuluru, R. *et al.* Classification of helpful comments on online suicide watch forums. In *Proceedings of the 7th ACM International Conference on Bioinformatics, Computational Biology, and Health Informatics*, 32–40 (2016).

[a625] Luo, J., Du, J., Tao, C., Xu, H. & Zhang, Y. Exploring temporal suicidal behavior patterns on social media: Insight from twitter analytics. *Health informatics journal* 1460458219832043 (2019).

[a626] Bryan, C. J. *et al.* Predictors of emerging suicide death among military personnel on social media networks. *Suicide and Life-Threatening Behavior* **48**, 413–430 (2018).

[a627] Watkins, N. G., Lovell, N. H. & Larsen, M. E. Smct-an innovative tool for mental health analysis of twitter data. In *2018 40th Annual International Conference of the IEEE Engineering in Medicine and Biology Society (EMBC)*, 4114–4117 (IEEE, 2018).

[a628] Salzmann-Erikson, M. & Hiçdurmaz, D. Use of social media among individuals who suffer from post-traumatic stress: A qualitative analysis of narratives. *Qualitative health research* **27**, 285–294 (2017).

[a629] Leis, A., Ronzano, F., Mayer, M. A., Furlong, L. I. & Sanz, F. Detecting signs of depression in tweets in spanish: behavioral and linguistic analysis. *Journal of medical Internet research* **21**, e14199 (2019).

[a630] DeJohn, A. D., Schulz, E. E., Pearson, A. L., Lachmar, E. M. & Wittenborn, A. K. Identifying and understanding communities using twitter to connect about depression: cross-sectional study. *JMIR mental health* **5**, e61 (2018).

[a631] Lachmar, E. M., Wittenborn, A. K., Bogen, K. W. & McCauley, H. L. # mydepressionlookslike: examining public discourse about depression on twitter. *JMIR Mental Health* **4**, e43 (2017).

[a632] Saha, B., Nguyen, T., Phung, D. & Venkatesh, S. A framework for classifying online mental health-related communities with an interest in depression. *IEEE journal of biomedical and health informatics* **20**, 1008–1015 (2016).

[a633] Kotliar, D. M. Depression narratives in blogs: A collaborative quest for coherence. *Qualitative health research* **26**, 1203–1215 (2016).

[a634] Mowery, D. *et al.* Understanding depressive symptoms and psychosocial stressors on twitter: a corpus-based study. *Journal of medical Internet research* **19**, e48 (2017).

[a635] Liu, S., Zhu, M., Yu, D. J., Rasin, A. & Young, S. D. Using real-time social media technologies to monitor levels of perceived stress and emotional state in college students: a web-based questionnaire study. *JMIR mental health* **4**, e2 (2017).

[a636] Yang, A. C., Huang, N. E., Peng, C.-K. & Tsai, S.-J. Do seasons have an influence on the incidence of depression? the use of an internet search engine query data as a proxy of human affect. *PloS one* **5**, e13728 (2010).

[a637] Reavley, N. J. & Pilkington, P. D. Use of twitter to monitor attitudes toward depression and schizophrenia: an exploratory study. *PeerJ* **2**, e647 (2014).

[a638] Doan, S., Ritchart, A., Perry, N., Chaparro, J. D. & Conway, M. How do you# relax when you're# stressed? a content analysis and infodemiology study of stress-related tweets. *JMIR public health and surveillance* **3**, e35 (2017).

[a639] Li, Q., Xue, Y., Zhao, L., Jia, J. & Feng, L. Analyzing and identifying teens' stressful periods and stressor events from a microblog. *IEEE journal of biomedical and health informatics* **21**, 1434–1448 (2016).

[a640] Michalak, E. E. *et al.* Bipolar disorder research 2.0: Web technologies for research capacity and knowledge translation. *Journal of evaluation in clinical practice* **23**, 1144–1152 (2017).

[a641] Budenz, A. *et al.* Mental illness and bipolar disorder on twitter: implications for stigma and social support. *Journal of Mental Health* **29**, 191–199 (2020).

[a642] Guntuku, S. C. *et al.* Studying expressions of loneliness in individuals using twitter: an observational study. *BMJ open* **9** (2019).

[a643] Pereira-Sanchez, V., Alvarez-Mon, M. A., Del Barco, A. A., Alvarez-Mon, M. & Teo, A. Exploring the extent of the hikikomori phenomenon on twitter: Mixed methods study of western language tweets. *Journal of medical Internet research* **21**, e14167 (2019).

[a644] Robinson, P., Turk, D., Jilka, S. & Cella, M. Measuring attitudes towards mental health using social media: investigating stigma and trivialisation. *Social psychiatry and psychiatric epidemiology* **54**, 51–58 (2019).

[a645] Larsen, M. E. *et al.* We feel: mapping emotion on twitter. *IEEE journal of biomedical and health informatics* **19**, 1246–1252 (2015).

[a646] Gruebner, O. *et al.* A novel surveillance approach for disaster mental health. *PLoS one* **12** (2017).

[a647] Gruebner, O. *et al.* Spatio-temporal distribution of negative emotions in new york city after a natural disaster as seen in social media. *International journal of environmental research and public health* **15**, 2275 (2018).

[a648] Jones, N. M., Brymer, M. & Silver, R. C. Using big data to study the impact of mass violence: opportunities for the traumatic stress field. *Journal of traumatic stress* **32**, 653–663 (2019).

[a649] Ofoghi, B., Mann, M. & Verspoor, K. Towards early discovery of salient health threats: A social media emotion classification technique. In *Biocomputing 2016: Proceedings of the Pacific Symposium*, 504–515 (World Scientific, 2016).

[a650] Kilgo, D. K., Yoo, J. & Johnson, T. J. Spreading ebola panic: Newspaper and social media coverage of the 2014 ebola health crisis. *Health communication* (2018).

[a651] Ren, F., Kang, X. & Quan, C. Examining accumulated emotional traits in suicide blogs with an emotion topic model. *IEEE journal of biomedical and health informatics* **20**, 1384–1396 (2015).

[a652] Li, A. *et al.* An analysis of stigma and suicide literacy in responses to suicides broadcast on social media. *Asia-Pacific Psychiatry* **10**, e12314 (2018).

[a653] Park, A., Conway, M. & Chen, A. T. Examining thematic similarity, difference, and membership in three online mental health communities from reddit: a text mining and visualization approach. *Computers in human behavior* **78**, 98–112 (2018).

[a654] Zhao, Y. *et al.* Assessing mental health signals among sexual and gender minorities using twitter data. In *2018 IEEE International Conference on Healthcare Informatics Workshop (ICHI-W)*, 51–52 (IEEE, 2018).

[a655] Arora, V. S., Stuckler, D. & Mckee, M. Tracking search engine queries for suicide in the united kingdom, 2004–2013. *Public health* **137**, 147–153 (2016).

[a656] Emma Hilton, C. Unveiling self-harm behaviour: what can social media site twitter tell us about self-harm? a qualitative exploration. *Journal of clinical nursing* **26**, 1690–1704 (2017).

[a657] Kang, S., Ha, J.-S. & Velasco, T. Attention deficit hyperactivity disorder on youtube: Framing, anchoring, and objectification in social media. *Community mental health journal* **53**, 445–451 (2017).

[a658] Hswen, Y., Gopaluni, A., Brownstein, J. S. & Hawkins, J. B. Using twitter to detect psychological characteristics of self-identified persons with autism spectrum disorder: a feasibility study. *JMIR mHealth and uHealth* **7**, e12264 (2019).

[a659] Saha, K., Weber, I., Birnbaum, M. L. & De Choudhury, M. Characterizing awareness of schizophrenia among facebook users by leveraging facebook advertisement estimates. *Journal of medical Internet research* **19**, e156 (2017).

[a660] Athanasopoulou, C. & Sakellari, E. Facebook and health information: Content analysis of groups related to schizophrenia. In *ICIMTH*, 255–258 (2015).

[a661] Naslund, J. A., Grande, S. W., Aschbrenner, K. A. & Elwyn, G. Naturally occurring peer support through social media: the experiences of individuals with severe mental illness using youtube. *PLOS one* **9**, e110171 (2014).

[a662] Hswen, Y., Naslund, J. A., Brownstein, J. S. & Hawkins, J. B. Online communication about depression and anxiety among twitter users with schizophrenia: preliminary findings to inform a digital phenotype using social media. *Psychiatric Quarterly* **89**, 569–580 (2018).

[a663] Hernandez, M. Y., Hernandez, M., Lopez, D. H., Gamez, D. & Lopez, S. R. What do health providers and patients tweet about schizophrenia? *Early Intervention in Psychiatry* (2019).

[a664] Anderson, J. G., Hundt, E., Dean, M., Keim-Malpass, J. & Lopez, R. P. "the church of online support" examining the use of blogs among family caregivers of persons with dementia. *Journal of Family Nursing* **23**, 34–54 (2017).

[a665] Wang, H.-W., Chen, D.-R., Yu, H.-W. & Chen, Y.-M. Forecasting the incidence of dementia and dementia-related outpatient visits with google trends: evidence from taiwan. *Journal of medical Internet research* **17**, e264 (2015).

[a666] Yoon, S. What can we learn about mental health needs from tweets mentioning dementia on world alzheimer's day? *Journal of the American Psychiatric Nurses Association* **22**, 498–503 (2016).

[a667] Robillard, J. M., Johnson, T. W., Hennessey, C., Beattie, B. L. & Illes, J. Aging 2.0: health information about dementia on twitter. *PLoS One* **8**, e69861 (2013).

[a668] Alvarez-Mon, M. A. *et al.* Areas of interest and stigmatic attitudes of the general public in five relevant medical conditions: Thematic and quantitative analysis using twitter. *Journal of medical Internet research* **21**, e14110 (2019).

[a669] Eli, K. Latin american countries lead in google search volumes for anorexia nervosa and bulimia nervosa: Implications for global mental health research. *International Journal of Eating Disorders* **51**, 1352–1356 (2018).

[a670] Wang, T., Mentzakis, E., Brede, M. & Ianni, A. Estimating determinants of attrition in eating disorder communities on twitter: an instrumental variables approach. *Journal of medical Internet research* **21**, e10942 (2019).

[a671] Novillo-Ortiz, D. & Hernández-Pérez, T. Social media in public health: an analysis of national health authorities and leading causes of death in spanish-speaking latin american and caribbean countries. *BMC medical informatics and decision making* **17**, 16 (2017).

[a672] Speaks, H., Falise, A., Grosgebauer, K., Duncan, D. & Carrico, A. Racial disparities in mortality among american film celebrities: A wikipedia-based retrospective cohort study. *Interactive Journal of Medical Research* **8**, e13871 (2019).

[a673] Meaney, S., Cussen, L., Greene, R. A. & O'Donoghue, K. Reaction on twitter to a cluster of perinatal deaths: a mixed method study. *JMIR public health and surveillance* **2**, e36 (2016).

[a674] Bundy, J. J. *et al.* #stroke. *Journal of NeuroInterventional Surgery* **10**, e33–e33 (2018).

[a675] Jain, M. J. & Mavani, K. J. A comprehensive study of worldwide selfie-related accidental mortality: a growing problem of the modern society. *International Journal of Injury Control and Safety Promotion* **24**, 544–549 (2017).

[a676] Parker, J., Cuthbertson, C., Loveridge, S., Skidmore, M. & Dyar, W. Forecasting state-level premature deaths from alcohol, drugs, and suicides using google trends data. *Journal of affective disorders* **213**, 9–15 (2017).

[a677] Hobbs, W. R., Burke, M., Christakis, N. A. & Fowler, J. H. Online social integration is associated with reduced mortality risk. *Proceedings of the National Academy of Sciences* **113**, 12980–12984 (2016).

[a678] Arnoux-Guenegou, A. *et al.* The adverse drug reactions from patient reports in social media project: Protocol for an evaluation against a gold standard. *JMIR research protocols* **8**, e11448 (2019).

[a679] Jamison, A. M. *et al.* Vaccine-related advertising in the facebook ad archive. *Vaccine* **38**, 512–520 (2020).

[a680] Lutkenhaus, R. O., Jansz, J. & Bouman, M. P. Mapping the dutch vaccination debate on twitter: Identifying communities, narratives, and interactions. *Vaccine: X* **1**, 100019 (2019).

[a681] Kang, G. J. *et al.* Semantic network analysis of vaccine sentiment in online social media. *Vaccine* **35**, 3621–3638 (2017).

[a682] Tafuri, S. *et al.* Communication about vaccinations in italian websites: a quantitative analysis. *Human vaccines & immunotherapeutics* **10**, 1416–1420 (2014).

[a683] Broniatowski, D. A. *et al.* Weaponized health communication: Twitter bots and russian trolls amplify the vaccine debate. *American journal of public health* **108**, 1378–1384 (2018).

[a684] Tomeny, T. S., Vargo, C. J. & El-Toukhy, S. Geographic and demographic correlates of autism-related anti-vaccine beliefs on twitter, 2009-15. *Social science & medicine* **191**, 168–175 (2017).

[a685] Blankenship, E. B. *et al.* Sentiment, contents, and retweets: A study of two vaccine-related twitter datasets. *The Permanente journal* **22** (2018).

[a686] Larson, H. J. *et al.* Measuring vaccine confidence: analysis of data obtained by a media surveillance system used to analyse public concerns about vaccines. *The Lancet infectious diseases* **13**, 606–613 (2013).

[a687] Schmidt, A. L., Zollo, F., Scala, A., Betsch, C. & Quattrociocchi, W. Polarization of the vaccination debate on facebook. *Vaccine* **36**, 3606–3612 (2018).

[a688] Covolo, L., Ceretti, E., Passeri, C., Boletti, M. & Gelatti, U. What arguments on vaccinations run through youtube videos in italy? a content analysis. *Human vaccines & immunotherapeutics* **13**, 1693–1699 (2017).

[a689] Alsyouf, M. *et al.* 'fake news' in urology: evaluating the accuracy of articles shared on social media in genitourinary malignancies. *BJU international* **124**, 701–706 (2019).

[a690] Meleo-Erwin, Z., Basch, C., MacLean, S. A., Scheibner, C. & Cadorett, V. "to each his own": Discussions of vaccine decision-making in top parenting blogs. *Human vaccines & immunotherapeutics* **13**, 1895–1901 (2017).

[a691] Bryan, M. A., Gunningham, H. & Moreno, M. A. Content and accuracy of vaccine information on pediatrician blogs. *Vaccine* **36**, 765–770 (2018).

[a692] Kearney, M. D., Selvan, P., Hauer, M. K., Leader, A. E. & Massey, P. M. Characterizing hpv vaccine sentiments and content on instagram. *Health Education & Behavior* **46**, 37S–48S (2019).

[a693] Du, J., Xu, J., Song, H.-Y. & Tao, C. Leveraging machine learning-based approaches to assess human papillomavirus vaccination sentiment trends with twitter data. *BMC medical informatics and decision making* **17**, 69 (2017).

[a694] Zhou, X. *et al.* Using social connection information to improve opinion mining: Identifying negative sentiment about hpv vaccines on twitter (2015).

[a695] Keelan, J., Pavri, V., Balakrishnan, R. & Wilson, K. An analysis of the human papilloma virus vaccine debate on myspace blogs. *Vaccine* **28**, 1535–1540 (2010).

[a696] Massey, P. M. *et al.* Applying multiple data collection tools to quantify human papillomavirus vaccine communication on twitter. *Journal of medical Internet research* **18**, e318 (2016).

[a697] Dunn, A. G., Leask, J., Zhou, X., Mandl, K. D. & Coiera, E. Associations between exposure to and expression of negative opinions about human papillomavirus vaccines on social media: an observational study. *Journal of medical Internet research* **17**, e144 (2015).

[a698] Surian, D. *et al.* Characterizing twitter discussions about hpv vaccines using topic modeling and community detection. *Journal of medical Internet research* **18**, e232 (2016).

[a699] Eichstaedt, J. C. *et al.* Psychological language on twitter predicts county-level heart disease mortality. *Psychological science* **26**, 159–169 (2015).

[a700] Du, J. *et al.* Leveraging deep learning to understand health beliefs about the human papillomavirus vaccine from social media. *NPJ digital medicine* **2**, 1–4 (2019).

[a701] Zhang, H. *et al.* Mining twitter to assess the determinants of health behavior toward human papillomavirus vaccination in the united states. *Journal of the American Medical Informatics Association* **27**, 225–235 (2020).

[a702] Powell, G. A. *et al.* Media content about vaccines in the united states and canada, 2012–2014: an analysis using data from the vaccine sentimeter. *Vaccine* **34**, 6229–6235 (2016).

[a703] Wiyeh, A. B. *et al.* Social media and hpv vaccination: Unsolicited public comments on a facebook post by the western cape department of health provide insights into determinants of vaccine hesitancy in south africa. *Vaccine* **37**, 6317–6323 (2019).

[a704] Budenz, A. *et al.* Hpv vaccine, twitter, and gay, bisexual and other men who have sex with men. *Health Promotion International* **35**, 290–300 (2020).

[a705] Krittanawong, C., Tunhasiriwet, A., Chirapongsathorn, S. & Kitai, T. Tweeting influenza vaccine to cardiovascular health community. *European Journal of Cardiovascular Nursing* **16**, 704–706 (2017).

[a706] Berlinberg, E. J., Deiner, M. S., Porco, T. C. & Acharya, N. R. Monitoring interest in herpes zoster vaccination: analysis of google search data. *JMIR Public Health and Surveillance* **4**, e10180 (2018).

[a707] Orr, D., Baram-Tsabari, A. & Landsman, K. Social media as a platform for health-related public debates and discussions: the polio vaccine on facebook. *Israel journal of health policy research* **5**, 1–11 (2016).

[a708] Gunaratne, K., Coomes, E. A. & Haghbayan, H. Temporal trends in anti-vaccine discourse on twitter. *Vaccine* **37**, 4867–4871 (2019).

[a709] Wang, J., Zhao, L., Ye, Y. & Zhang, Y. Adverse event detection by integrating twitter data and vaers. *Journal of biomedical semantics* **9**, 19 (2018).

[a710] Suragh, T. A. *et al.* Cluster anxiety-related adverse events following immunization (aefi): an assessment of reports detected in social media and those identified using an online search engine. *Vaccine* **36**, 5949–5954 (2018).

[a711] Dunn, A. G. *et al.* Mapping information exposure on social media to explain differences in hpv vaccine coverage in the united states. *Vaccine* **35**, 3033–3040 (2017).

[a712] Dyda, A. *et al.* Hpv vaccine coverage in australia and associations with hpv vaccine information exposure among australian twitter users. *Human vaccines & immunotherapeutics* **15**, 1488–1495 (2019).

[a713] Pías-Peleteiro, L., Cortés-Bordoy, J. & Martinón-Torres, F. Dr google: What about the human papillomavirus vaccine? *Human vaccines & immunotherapeutics* **9**, 1712–1719 (2013).

[a714] Habel, M. A., Liddon, N. & Stryker, J. E. The hpv vaccine: a content analysis of online news stories. *Journal of women's health* **18**, 401–407 (2009).

[a715] Keim-Malpass, J., Mitchell, E. M., Sun, E. & Kennedy, C. Using twitter to understand public perceptions regarding the# hpv vaccine: opportunities for public health nurses to engage in social marketing. *Public Health Nursing* **34**, 316–323 (2017).

[a716] Lama, Y., Hu, D., Jamison, A., Quinn, S. C. & Broniatowski, D. A. Characterizing trends in human papillomavirus vaccine discourse on reddit (2007-2015): an observational study. *JMIR public health and surveillance* **5**, e12480 (2019).

[a717] Massey, P. M. *et al.* What drives health professionals to tweet about# hpvvaccine? identifying strategies for effective communication. *Preventing chronic disease* **15** (2018).

[a718] Basch, C. H. & MacLean, S. A. A content analysis of hpv related posts on instagram. *Human vaccines & immunotherapeutics* **15**, 1476–1478 (2019).

[a719] Covolo, L. *et al.* How has the flu virus infected the web? 2010 influenza and vaccine information available on the internet. *BMC Public Health* **13**, 83 (2013).

[a720] Shah, M. P. *et al.* Use of internet search data to monitor rotavirus vaccine impact in the united states, united kingdom, and mexico. *Journal of the Pediatric Infectious Diseases Society* **7**, 56–63 (2018).

[a721] Radzikowski, J. *et al.* The measles vaccination narrative in twitter: a quantitative analysis. *JMIR public health and surveillance* **2**, e1 (2016).

[a722] Arif, N. *et al.* Fake news or weak science? visibility and characterization of antivaccine webpages returned by google in different languages and countries. *Frontiers in immunology* **9**, 1215 (2018).

[a723] Han, L., Han, L., Darney, B. & Rodriguez, M. I. Tweeting pp: an analysis of the 2015–2016 planned parenthood controversy on twitter. *Contraception* **96**, 388–394 (2017).

[a724] Baazeem, M. & Abenhaim, H. Google and women's health-related issues: what does the search engine data reveal? *Online journal of public health informatics* **6** (2014).

[a725] Fioretti, B., Reiter, M., Betrán, A. & Torloni, M. Googling caesarean section: a survey on the quality of the information available on the internet. *BJOG: An International Journal of Obstetrics & Gynaecology* **122**, 731–739 (2015).

[a726] Thompson, E. L. *et al.* Rethinking preconception care: a critical, women's health perspective. *Maternal and child health journal* **21**, 1147–1155 (2017).

[a727] Gesualdo, F. *et al.* Does googling for preconception care result in information consistent with international guidelines: a comparison of information found by italian women of childbearing age and health professionals. *BMC Medical Informatics and Decision Making* **13**, 14 (2013).

[a728] D'Ambrosio, A. *et al.* Web-based surveillance of public information needs for informing preconception interventions. *PloS one* **10**, e0122551 (2015).

[a729] Wood, L. N. *et al.* Public awareness of uterine power morcellation through us food and drug administration communications: Analysis of google trends search term patterns. *JMIR public health and surveillance* **4**, e47 (2018).

[a730] Salem, J. *et al.* Online discussion on# kidneystones: a longitudinal assessment of activity, users and content. *PloS one* **11**, e0160863 (2016).

[a731] Dreher, P. C., Tong, C., Ghiraldi, E. & Friedlander, J. I. Use of google trends to track online behavior and interest in kidney stone surgery. *Urology* **121**, 74–78 (2018).

[a732] Ahmed, S. *et al.* User-driven conversations about dialysis through facebook: a qualitative thematic analysis. *Nephrology* **22**, 301–307 (2017).

[a733] Rossignol, L. *et al.* A method to assess seasonality of urinary tract infections based on medication sales and google trends. *PloS one* **8**, e76020 (2013).

[a734] Lotto, M., Aguirre, P. E., Strieder, A. P., Cruvinel, A. F. & Cruvinel, T. Levels of toothache-related interests of google and youtube users from developed and developing countries over time. *PeerJ* **7**, e7706 (2019).

[a735] Cruvinel, T. *et al.* Digital behavior surveillance: monitoring dental caries and toothache interests of google users from developing countries. *Oral diseases* **25**, 339–347 (2019).

[a736] Heaivilin, N., Gerbert, B., Page, J. & Gibbs, J. Public health surveillance of dental pain via twitter. *Journal of dental research* **90**, 1047–1051 (2011).

[a737] Strieder, A. P., Aguirre, P. E. A., Lotto, M., Cruvinel, A. F. P. & Cruvinel, T. Digital behavior surveillance for monitoring the interests of google users in amber necklace in different countries. *International journal of paediatric dentistry* **29**, 603–614 (2019).

[a738] Russo, G. I. *et al.* Consulting "dr google" for sexual dysfunction: a contemporary worldwide trend analysis. *International journal of impotence research* 1–7 (2019).

[a739] Sansone, A. *et al.* The sentiment analysis of tweets as a new tool to measure public perception of male erectile and ejaculatory dysfunctions. *Sexual Medicine* **7**, 464–471 (2019).

[a740] Muellner, U. J. *et al.* Timely reporting and interactive visualization of animal health and slaughterhouse surveillance data in switzerland. *Frontiers in veterinary science* **2**, 47 (2015).

[a741] Abreo, N. A. S., Thompson, K. F., Arabejo, G. F. P. & Superio, M. D. A. Social media as a novel source of data on the impact of marine litter on megafauna: The philippines as a case study. *Marine pollution bulletin* **140**, 51–59 (2019).

[a742] Qiu, R., Hadzikadic, M., Yu, S. & Yao, L. Estimating disease burden using internet data. *Health informatics journal* **25**, 1863–1877 (2019).

[a743] Whitsitt, J., Karimkhani, C., Boyers, L. N., Lott, J. P. & Dellavalle, R. P. Comparing burden of dermatologic disease to search interest on google trends. *Dermatology online journal* **21** (2015).

[a744] Reddy, D. & Colman, E. A comparative toxidrome analysis of human organophosphate and nerve agent poisonings using social media. *Clinical and translational science* **10**, 225–230 (2017).

[a745] Vilela, R. *et al.* social network for the surveillance and prevention of workplace accidents. *Work* **41**, 3123–3129 (2012).

[a746] Bragazzi, N. L., Dini, G., Toletone, A., Brigo, F. & Durando, P. Leveraging big data for exploring occupational diseases-related interest at the level of scientific community, media coverage and novel data streams: the example of silicosis as a pilot study. *PLoS One* **11**, e0166051 (2016).

[a747] Workewych, A. M. *et al.* Twitter and traumatic brain injury: A content and sentiment analysis of tweets pertaining to sport-related brain injury. *SAGE open medicine* **5**, 2050312117720057 (2017).

[a748] Jha, A., Lin, L. & Savoia, E. The use of social media by state health departments in the us: analyzing health communication through facebook. *Journal of community health* **41**, 174–179 (2016).

[a749] Novillo-Ortiz, D., Hernández-Pérez, T. & Saigí-Rubió, F. Availability of information in public health on the internet: An analysis of national health authorities in the spanish-speaking latin american and caribbean countries. *International journal of medical informatics* **100**, 46–55 (2017).

[a750] Alnemer, K. A. *et al.* Are health-related tweets evidence based? review and analysis of health-related tweets on twitter. *Journal of medical Internet research* **17**, e246 (2015).

[a751] Denecke, K. *et al.* How to exploit twitter for public health monitoring? *Methods of information in medicine* **52**, 326–339 (2013).

[a752] Priedhorsky, R. *et al.* Measuring global disease with wikipedia: Success, failure, and a research agenda. In *Proceedings of the 2017 ACM Conference on Computer Supported Cooperative Work and Social Computing*, 1812–1834 (2017).

[a753] Weeg, C. *et al.* Using twitter to measure public discussion of diseases: a case study. *JMIR public health and surveillance* **1**, e6 (2015).

[a754] Huber, J. *et al.* Social media research strategy to understand clinician and public perception of health care messages. *JDR Clinical & Translational Research* **5**, 71–81 (2020).

[a755] Al Khaja, K. A., AlKhaja, A. K. & Sequeira, R. P. Drug information, misinformation, and disinformation on social media: a content analysis study. *Journal of public health policy* **39**, 343–357 (2018).
